# Synthesis, molecular modeling, and biological evaluation of novel imatinib derivatives as anticancer agents

**DOI:** 10.3906/kim-2107-23

**Published:** 2021-09-06

**Authors:** Fulya GÜNAY, Sevcan BALTA, Yuk Yin NG, Özlem ULUCAN, Zuhal TURGUT, Ömer Tahir GÜNKARA

**Affiliations:** 1 Department of Chemistry, Faculty of Arts and Science, Yıldız Technical University, İstanbul, Turkey; 2 Department of Genetics and Bioengineering, Faculty of Engineering and Natural Sciences, İstanbul Bilgi University, İstanbul, Turkey

**Keywords:** Imatinib derivatives, tyrosine kinase inhibitors, BCR-ABL inhibitors, leukemia, anti-cancer agents, molecular docking

## Abstract

Different derivatives of imatinib were synthesized by a 3-step reaction method. The structures of the new compounds were characterized by spectroscopic methods. For quantitative evaluation of the biological activity of the compounds, MTT assays were performed, where four BCR-ABL negative leukemic cell lines (Jurkat, Reh, Nalm-6 and Molt-4), one BCR-ABL positive cell line (K562), and one non-leukemic cell line (Hek293T) were incubated with various concentrations of the derivatives. Although imatinib was specifically designed for the BCR-ABL protein, our results showed that it was also effective on BCR-ABL negative cell lines except for Reh cell line. Compound 9 showed lowest IC_50_ values against Nalm-6 cells as 1.639 μM, also the values of Compound 10 for each cell were very close to imatinib. Molecular docking simulations suggest that except for compound 6, the compounds prefer a DFG-out conformation of the ABL kinase domain. Among them, compound 10 has the highest affinity for ABL kinase domain that is close to the affinity of imatinib. The common rings between compound 10 and imatinib adopt exactly the same conformation and same type of interactions in the ATP binding site with the ABL kinase domain.

## 1. Introduction

**Figure 1:**
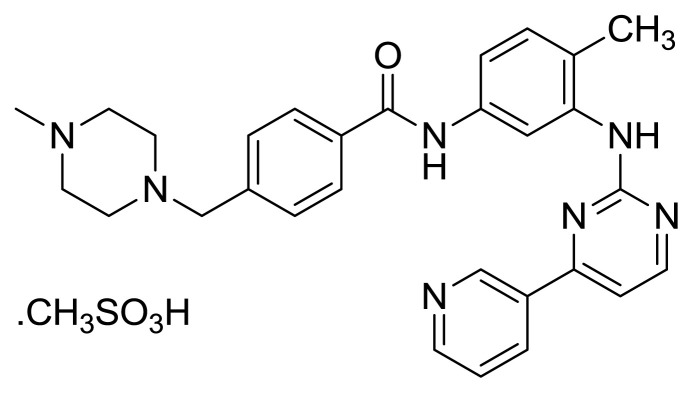
Imatinib mesylate molecular structure.

Imatinib mesylate (Gleevec®, STI-571), (Figure 1) is the first generation of FDA approved protein-tyrosine kinaseinhibitor, especially treatment of CML (Chronic Myelogenous Leukemia) and GIST (Gastrointestinal Stromal Tumors)[1–6]. It contains 2-(phenylamino)pyrimidine heterocycle core that functions for targeting BCR-ABL (the BreakpointCluster Region-the Abelson proto-oncogene) activity leading to decrease tyrosine kinase activity; methyl group onthis core occupies the selectivity to BCR-ABL; aryl piperazine core increases oral bioavailability and pyrimidine coreoccupies cellular activity [7]. There are three different general methods for the synthesis of imatinib mesylate [8–14].There are also some patents [8], [15–19] in which imatinib is obtained by directly reacting the commercially soldaminopyrimidine and the aryl piperazine derivatives. Moreover, there are flow-based [13], [20–22], microwave-asisted[23], copper-catalyzed [24], palladium-catalyzed [25], BrettPhos-catalyzed [26–27] methods for synthesizing imatinib.

Imatinib is specific for the tyrosine kinase domain in ABL and BCR-ABL gene products, it has been reported that treatment with BCR-ABL inhibitors, significantly reduces the application of hematopoietic cell transplantation for treatment of CML [28]. It binds ABL1 kinase in ATP-binding site and stabilizes an inactive conformation of the catalytic domain where the well-known “DFG” triad (Asp-Phe-Gly) is in an out conformation [1].

After prolonged treatment of imatinib, due to mutations at kinase domain site in ATP binding site, the drug activity changes. Over-stimulated BCR-ABL1 fusion protein causes genomic instability in CML stem cells and causes more than 50 hotspot mutations to accumulate in the ABL1 kinase domain. Complexity or having more than one mutation also changes the patient’s outcome against the drug. ABL1 point mutations reduce the accessibility of the drug’s binding site, limiting the enzyme’s flexibility [28–29].

For this reason, PD180970, CGP76030, BMS-354825, AMN 107 or Nilotinib, and, more recently, AP24534 have been developed. There are also novel approaches to imatinib resistance, such as Farnesyltransferase inhibitors (SCH66336) and Proteasome inhibitors (Bortezomib), that have been reported to have growth inhibitory properties on leukemia [30–36].

In this study, it was aimed to synthesize and investigate the biological activity of new imatinib derivatives that potently inhibit the growth of cancer cells. The designed compounds were synthesized, and their structural formula was confirmed by different spectral data. Anti-cancer activities of all the newly synthesized compounds were examined by MTT (3-(4,5-dimethylthiazol-2-yl)-2,5-diphenyltetrazolium bromide) assay.

Additionally, we performed extensive molecular docking simulations to evaluate the binding preference of newly synthesized compounds to different conformations of wild type ABL and BRAF (v-Raf murine sarcoma viral oncogene homolog B) kinases.

## 2. Materials and methods

### 2.1. Chemistry

#### 2.1.1. Chemicals and instruments

All chemicals used were purchased from Merck and Aldrich without further purification. Trimetazidine HCl was obtained from the World Medicine Drug Company (Fisher Scientific), Imatinib was purchased from Sigma Aldrich (SML1027-100MG). Sonication was performed in an Intersonik ultrasound cleaner (model: MIN4) with a frequency of 25 kHz, an US output power of 100 W, a heating 200 W. Heidolph RV Laborata 4000 rotary evaporator was used to evaporate the solvent. A TLC Merck 5554 with silica gel layers with fluorescent indicator and a Camag (254/366 nm) UV lamp were used. The melting points of the pure materials were measured on the Stuart apparatus. FTIR spectra of the starting materials and the obtained products were taken on a “Perkin Elmer Spectrum One” FTIR spectrometer by ATR technique. ^1^H and ^13^C NMR spectra were obtained from a “Bruker 500 MHz” spectrometer in CDCl_3_. Chemical shifts were reported in ppm (parts per million) with respect to internal standard TMS (Tetramethylsilane). LC-MS spectra were obtained by Agilient 6200 series TOF/6500 series TOF/Q-TOF Mass Spectrometer. All crude products were purified with Teledyne Isco CombiFlash Rf 200 system and RediSep Rf Gold Silica Columns.

#### 2.1.2. General procedure for the synthesis of intermediate containing benzamide

1 mmol (186.05 mg) 3-bromo-4-methylaniline (Compound 2) was placed in a two-necked round bottom flask. It was dissolved in dry DCM (dichloromethane) (4 mL) under inert atmosphere at 0°C. 1 mmol (138.205 mg) of overheated K_2_CO_3_ was added the reaction mixture and stirred at the same temperature. After that, 1 mmol (189.04 mg) 4-chloromethylbenzoylchloride (Compound 1) was dissolved with dry DCM (4 mL) and injected dropwise into the reaction medium. While continuing the addition, yellow crystals formed in the reaction medium. The mixture was stirred at 0°C for 1 h. After completion of the reaction, the yellow crystals were filtered, washed with DCM, and dried [13, 20–21].

##### 2.1.2.1. *N*-(3-Bromo-4-methylphenyl)-4-(chloromethyl)benzamide (Compound 3) [13]

Yellow solid, (This compound was crystallized with DCM, yield: 98%, Rf: 0.54 (1:3 Ethylacetate/n-Hexane), m.p. 157°C); IR (ATR) n_max_ 3452 (NH), 3284, 2985, 1641 (C=O), 1577, 1499, 1441, 1384, 1299 cm^−1^; ^1^H NMR (600 MHz, CDCl_3_): δ = 2.38 (s, 3H, CH_3_), 4.63 (s, 2H, CH_2_), 7.21 (d, J=8.2 Hz, 1H, Ar-H), 7.47 (d, J=1.5 Hz, 1H, Ar-H), 7.51 (d, J=8.5 Hz, 2H, Ar-H), 7.84 (brds, 1H, NH), 7.85 (dd, J= 8.5,1.7 Hz, 2H, Ar-H) 7.89 (s, 1H, Ar-H) ppm; ^13^C NMR (150 MHz, CDCl_3_): δ = 22.35 (CH_3_), 45.29 (CH_2_), 119.17 (CAr), 123.95 (CAr), 124.89 (CAr), 127.49 (CAr), 128.99 (2xCAr), 130.91 (2xCAr), 134.21 (CAr), 134.59 (Cq), 136.55 (Cq), 141.42 (Cq), 164.97 (C=O) ppm.

### 2.1.3. General procedure for the synthesis of intermediates containing aryl piperazine (Compound 4a–4b)

0.3 mmol (101.59 mg) *N*-(3-Bromo-4-methylphenyl)-4-(chloromethyl)benzamide (Compound 3) and 0.39 mmol aryl piperazine [2-(piperazin-1-yl)pyrimidine or 1-(2,3,4-trimethoxybenzyl) piperazine dihydrochloride)] were taken into a round bottom flask. The starting materials were dissolved in dry acetone (10 mL) under inert atmosphere. Then, 0.9 mmol (124.38 mg) of overheated K_2_CO_3_ was added to the reaction mixture. The mixture was heated and stirred at 55°C overnight under inert atmosphere. After completion of the reaction, the reaction mixture was filtered and the solvent was evaporated under reduced pressure. The crude products 4a and 4b were purified using flash chromatography.

#### 2.1.3.1. *N*-(3-Bromo-4-methylphenyl)-4-((4-(pyrimidin-2-yl)piperazin-1-yl)methyl)benzamide (Compound 4a)

Brownish gummy solid (This compound was purified by flash column chromatography using ethylacetate/*n*-hexane 5:1 as eluent, Rf: 0.45 (5:1 ethylacetate/*n*-hexane), yield : 60%, m.p. 105°C); IR (ATR) n_max_ 3283 (br) (NH), 3081 (w), 2991 (w), 2947 (w), 1737 (s) (C=O) 1252 (vs), 1017 (vs) cm-^1^; ^1^H NMR (500 MHz, CDCl_3_): δ 2.37 (s, 3H, CH_3_), 2.50 (t, J= 2.4 Hz, 4H, N-CH_2_), 3.60 (s, 2H, CH_2_), 3.83 (t, J= 2.4 Hz, 4H, N-CH_2_), 6.48 (t, J= 4.7 Hz, 1H, Ar-H), 7.19 (d, J= 8.3 Hz, 1H, Ar-H), 7.46 (d, J= 8.4 Hz, 2H, Ar-H), 7.48–7.49 (m, 1H, Ar-H), 7.81 (d, J= 8.2 Hz, 2H, Ar-H), 7.88 (d, J= 2.2 Hz, 1H, Ar-H), 7.94 (brds, 1H, NH), 8.29 (d, J= 4.7 Hz, 2H, Ar-H) ppm; ^13^C NMR (125 MHz, CDCl_3_) δ 22.3 (CH_3_), 43.7 (2x*N*-CH_2_), 53.0 (2x*N*-CH_2_), 62.6 (CH_2_), 109.8 (CAr), 119.2 (CAr), 123.9 (CAr), 124.8 (Cq), 127.1 (2xCAr), 129.4 (2xCAr), 130.8 (CAr), 133.5 (Cq), 133.9 (Cq), 136.8 (Cq), 142.6 (Cq), 157.7 (2xCAr), 165.5 (C=O) ppm; HRMS (ESI^+^) m/z calcd for [C_23_H_24_BrN_5_O]+H^+^ 467.3786, found 468.1215 ([C_23_H_24_BrN_5_O_4_]+H)^+^.

#### 2.1.3.2. *N*-(3-Bromo-4-metylphenyl)-4-((4-(2,3,4-trimethoxybenzyl)piperazin-1-yl)methyl)benzamide (Compound 4b)

Pale yellowish gummy solid (This compound was purified by flash column chromatography using DCM/MeOH 10:1 as eluent, Rf : 0.54 (10:1 DCM/MeOH), yield 46.3%; m.p.135°C); IR (ATR): n_max_ 3290 (br) (NH), 2936 (m), 2819 (m), 1651 (s) (C=O), 1235 (m), 1093 (vs) cm^−1^; ^1^H NMR (500 MHz, CDCl_3_): δ = 2.37 (s, 3H, CH_3_), 2.48 (brds, 8H, N-CH_2_), 3.50 (s, 2H, CH_2_), 3.55 (s, 2H, CH_2_), 3.85 (s, 3H, O-CH_3_), 3.86 (s, 3H, O-CH_3_), 3.88 (s, 3H, O-CH_3_), 5.30, 6.62 (d, J = 8.20, 1.5 Hz, 1H, Ar-H), 6.64 (d, J= 8.60,1H, Ar-H) 6.98 (d, J= 8.60 Hz, 1H, Ar-H), 7.19–7.21 (dd, J=8.2, 0.5 Hz, 1H, Ar-H), 7.27 7.40–7.42 (dd, J=1.5 0.5Hz, 1H) 7.48–7.50 (ddd, J=8.5 1.4, 0.5 Hz, 2H, Ar-H), 7.78–7.88 (ddd, J= 8.5,1.7, 0.5 Hz, 2H, Ar-H) ppm; ^13^C NMR (125 MHz, CDCl_3_): δ = 22.29 (CH_3_), 52.84 (CH_2_), 53.20 (CH_2_), 55.95 (2xCH_2_) 56.00 (2xCH_2_), 60.68 (O-CH_3_), 61.13 (O-CH_3_), 62.51 (O-CH_3_), 106.96 (CAr), 119.10 (CAr), 123.86 (CAr), 124.78 (CAr), 125.16 (CAr), 126.94 (2xCAr), 129.36 (2xCAr), 130.81 (CAr), 133.32 (Cq), 133.87 (Cq), 136.79 (Cq), 142.89 (Cq), 152.63 (Cq),152.90(Cq), 165.46 (C=O) ppm; HRMS (ESI^+^) m/z calcd for [C_29_H_34_BrN_3_O_4_]+H^+^ 569.5099, found 569.5200 ([C_29_H_34_BrN_3_O_4_]+H)^+^.

#### 2.1.4. General procedure for the synthesis of intermediates containing morpholine (Compound 4c)

0.3 mmol (116.79 mg) of N-(3-Bromo-4-methylphenyl)-4-(chloromethyl)benzamide (Compound 3) was taken into a round bottom flask and 2.6 mmol (226.46 mg) morpholine was added onto it. The reaction mixture was sonicated at 55°C for 30 minutes without solvent. After completion of the reaction, the crude product (4c) was recrystallized with ethyl acetate.

##### 2.1.4.1. *N*-(3-Bromo-4-methylphenyl)-4-(morpholinomethyl)benzamide (Compound 4c)

Cream-white, gummy solid; (This compound was purified by crystallization with EtOAc, Rf: 0.7 (7:1 DCM/MeOH), yield 76.7%; m.p.116°C); IR (ATR) n_max_ 3268 (s) (NH), 3023 (w), 2920 (m), 1636 (vs) (C=O), 1260 (w), 1036 (vs) cm^−1^; ^1^H NMR (500 MHz, CDCl_3_): δ = 2.38 (s, 3H, CH_3_), 2.52 (brds, 4H, N-CH_2_), 3.62 (s, 2H, CH_2_), 3.75 (t, J= 2.5 Hz, 4H, O-CH_2_), 7.20 (d, J= 8.4 Hz, 1H, Ar-H), 7.48 (m, 3H, Ar-H), 7.82 (d, J= 8.2 Hz, 2H, Ar-H), 7.92 (d, J= 2.1 Hz, 1H, Ar-H), 7.97 (brds, 1H, NH) ppm; ^13^C NMR (125 MHz, CDCl_3_): δ = 22.3 (CH_3_), 43.3 (CH_2_), 53.5 (CH_2_), 62.7 (CH_2_), 63.8 (CH_2_), 66.6 (CH_2_), 119.2 (CAr), 123.9 (CAr), 124.8 (Cq), 127.2 (2xCAr), 129.7 (2xCAr), 130.8 (CAr), 133.8 (Cq), 134.0 (Cq), 136.7 (Cq), 165.4 (C=O) ppm; HRMS (ESI^+^) m/z calcd for [C_19_H_21_BrN_2_O_2_]+H^+^ 390.2942, found 390.1805 [(C_19_H_21_BrN_2_O_2_)+H]^+^.

#### 2.1.5. General Procedure for the Synthesis of Imatinib Derivatives (Compound 5–10)

In a 10 mL oven dried schlenk tube, equipped with a rubber septum, 0.214 mmol compound 4a, 4b or 4c, 0.214 mmol corresponding aromatic amine [2-amino-4-methylpyrimidine, 2-aminopyrazine or 2-amino-4-methylpyridine] 0.297 mmol (33.4 mg) Potassium tert-butoxide, 0.01712 mmol (9.9 mg) Xphos, 0.00856 mmol (7.8 mg) Tris (dibenzylidenacetone) dipalladium(0) were added. Subsequently, 1 mL dry toluene and 1 mL dry tert-butanol were added into the tube, and the resulting mixture was heated and stirred at 160°C for 24 h under inert atmosphere. The reaction completion was monitored by TLC. Upon completion of the reaction, the mixture was filtered through celite, washed with DCM, then the solvent was evaporated under reduced pressure. The residue was purified by flash chromatography.

##### 2.1.5.1. N-(4-Methyl-3-((4-methylpyrimidin-2-yl)amino)phenyl)-4-((4-(pyrimidin-2-yl)piperazin-1-yl)methyl)benzamide (Compound 5)

Brownish gummy solid; (This compound was purified by flash column chromatography using DCM/MeOH 10:1 as eluent, 0.54 (10:1 DCM/MeOH), yield 40.0%; m.p.89°C); IR (ATR) n_max_ 3280 (br) (NH), 3026 (w), 2850 (w), 1651 (s) (C=O), 1219 (w), 1029 (s) cm^−1^; ^1^H NMR (500 MHz, CDCl_3_): δ = 2.32 (s, 3H, CH_3_), 2.43 (s, 3H, CH_3_), 2.52 (brds, 4H, *N*-CH_2_), 3.49 (brds, 1H, NH), 3.61 (s, 2H, CH_2_), 3.84 (t, J= 2.5 Hz, 4H, N-CH_2_), 6.48 (t, J= Hz, 1H, Ar-H), 6.75 (brds, 1H, NH), 7.16–7.21 (m, 1H, Ar-H), 7.44–7.51 (m, 3H, Ar-H), 7.79–7.88 (m, 3H, Ar-H), 8.28–8.30 (m, 3H, Ar-H), 8.38–8.40 (m, 1H, Ar-H) ppm; ^13^C NMR (125 MHz, CDCl_3_): δ = 19.4 (CH_3_), 22.3 (CH_3_), 43.6 (2xN-CH_2_), 52.9 (2xN-CH_2_), 62.6 (CH_2_), 109.8 (CAr), 119.1 (CAr), 123.8 (CAr), 124.7 (Cq), 127.0 (3xCAr), 129.3 (3xCAr), 130.7 (CAr), 133.3 (2xCq), 133.8 (Cq), 136.7 (2xCq), 142.5 (Cq), 157.6 (2xCAr), 161.6 (Cq), 165.5 (C=O) ppm; HRMS (ESI^+^) m/z calcd for [C_28_H_30_N_8_O]+H^+^ 495.2621, found 495.2607 [(C_28_H_30_N_8_O)+H]^+^.

##### 2.1.5.2. *N*-(4-Methyl-3-((4-methylpyrimidin-2-yl)amino)phenyl)-4-((4-(2,3,4-trimethoxyphenyl)piperazin-1-yl)methyl)benzamide (Compound 6)

Yellowish gummy solid (This compound was purified by flash column chromatography using DCM/MeOH 7:1 as eluent, 0.50 (7:1 DCM/MeOH), yield 58.1%; dcm.p.320°C); IR (ATR) n_max_ 3293 (br) (NH), 2932 (s), 1651 (s) (C=O), 1200 (m), 1093 (vs) cm^−1^ ; ^1^H NMR (500 MHz, CDCl_3_): δ = 2.28 (s, 3H, CH_3_), 2.41 (s, 3H, CH_3_), 2.50 (brds, 8H, N-CH_2_), 3.52 (s, 2H, CH_2_), 3.54 (s, 2H, CH_2_), 3.85 (s, 3H, O-CH_3_), 3.86 (s, 3H, O-CH_3_), 3.88 (s, 3H, O-CH_3_), 6.59 (d, J= 5.0 Hz, 1H, Ar-H), 6.62 (d, J= 8.5 Hz, 1H, Ar-H), 6.92 (brds, 1H, NH), 6.99 (d, J= 8.5 Hz, 1H, Ar-H), 7.16 (d, J= 8.3 Hz, 1H, Ar-H), 7.38 (d, J=8.1 Hz, 1H, Ar-H), 7.44 (d, J= 7.3 Hz, 1H, Ar-H), 7.78 (d, J= 8.1 Hz, 2H, Ar-H), 8.03 (brds, 1H, NH), 8.25 (d, J= 5.0 Hz, 1H, Ar-H), 8.33 (d, J= 1.9 Hz, 1H, Ar-H) ppm; ^13^C NMR (125 MHz, CDCl_3_): δ = 17.6 (CH_3_), 24.2 (CH_3_), 52.7 (2xN-CH_2_), 55.9 (2xN-CH_2_), 61.2 (CH_2_), 106.1 (CAr), 112.3 (CAr), 115.2 (CAr), 123.9 (Cq), 127.0 (CAr), 129.3 (3xCAr), 134.0 (Cq), 136.5 (Cq), 138.0 (Cq), 142.2 (Cq), 152.7 (2xCAr), 161.1 (C=O) ppm; HRMS (ESI^+^) m/z calcd for [C_33_H_38_N_6_O_4_]+H^+^ 597.7271, found 597.7280 ([C_33_H_38_N_6_O_4_]+H)^+^.

##### 2.1.5.3. *N*-(4-Methyl-3-(pyrazin-2-ylamino)phenyl)-4-((4-(pyrimidin-2-yl)piperazin-1-yl)methyl)benzamide (Compound 7)

Yellowish gummy solid (This compound was purified by flash column chromatography using DCM/MeOH 10:1 as eluent, Rf: 0.60 (10:1 DCM/MeOH), yield 50.8%, m.p.143°C; IR (ATR) n_max_ 3291 (br) (NH), 2919 (m), 2812 (w), 1651 (s) (C=O), 1583 (vs), 1546 (s), 1493 (vs), 1445 (s), 1142 (w), 1005 (m) cm^−1^; ^1^H NMR (500 MHz, CDCl_3_): δ = 2.39 (s, 3H, CH_3_), 2.53 (brds, 4H, N-CH_2_), 3.52 (s, 2H, CH_2_), 3.86 (brds, 4H, N-CH_2_), 6.50 (t, J= 4.6 Hz, 1H, Ar-H), 7.21 (d, J=8.2 Hz, 2H, Ar-H), 7.28 (brds, 1H, NH), 7.48 (m, 5H, Ar-H), 7.83 (d, J= 7.7 Hz, 2H, Ar-H), 7.91 (s, 1H, ArH), 7.95 (brds, 1H, NH), 8.31 (d, J= 4.6 Hz, 2H, Ar-H) ppm; ^13^C NMR (125 MHz, CDCl_3_): δ= 22.3 (CH_3_), 43.7 (2xCH_2_), 53.0 (2xCH_2_), 62.6 (CH_2_), 109.9 (CAr), 119.2 (2xCAr), 123.9 (2xCAr), 124.8 (Cq), 127.1 (2xCAr), 129.4 (2xCAr), 130.8 (2xCAr), 133.5 (Cq), 133.9 (2xCq), 136.8 (Cq), 142.5 (Cq), 157.7 (2xCAr), 161.6 (Cq), 165.5 (C=O) ppm; HRMS (ESI^+^) m/z calcd for [C_27_H_28_N_8_O]+H^+^ 481.5722, found 481.3640 ([C_27_H_28_N_8_O]+H)^+^.

##### 2.1.5.4. *N*-(4-Methyl-3-((4-methylpyrimidin-2-yl)amino)phenyl)-4-(morhpolinomethyl)benzamide (Compound 8)

Cream-white gummy solid (This compound was purified by flash column chromatography using DCM/MeOH 10:1 as eluent, Rf: 0.40 (10:1 DCM/MeOH), yield 54.8%, m.p.157°C); IR (ATR): n= 3286 (br) (NH), 3084 (w), 2922 (m), 2854 (m), 1711 (s) (C=O), 1261 (m), 1006 (w) cm^−1^. ^1^H NMR (500 MHz, CDCl_3_): δ = 2.32 (s, 3H, CH_3_), 2,38 (s, 3H, CH_3_), 2.52 (brds, 4H, N-CH_2_), 3.62 (s, 2H, CH_2_), 3.98 (t, J= 2.2 Hz, 4H, O-CH_2_), 4.03 (brds, 1H, NH), 6.93 (d, J= 2.8 Hz, 1H, ArH), 7.20 (d, J= 3.2 Hz, 1H, Ar-H), 7.48–7.53 (m, 3H, Ar-H), 7.84 (d, J= 4.2 Hz, 2H, Ar-H), 7.92 (s, 1H, ArH), 7.97 (brds, 1H, NH), 8.36 (d, J= 2.8 Hz, 1H, ArH) ppm; ^13^C NMR (125 MHz, CDCl_3_): δ = 22.3 (CH_3_), 25.9 (CH_3_), 43.5 (N-CH_2_), 53.6 (N-CH_2_), 62.4 (O-CH_2_), 62.6 (CH_2_), 66.6 (O-CH_2_), 110.2 (CAr), 119.1 (CAr), 123.86 (CAr), 124.8 (Cq), 127.2 (3xCAr), 129.6 (2xCAr), 130.7 (CAr), 133.5 (Cq), 133.9 (Cq), 136.7 (Cq), 156.3 (Cq), 165.3 (Cq), 165.5 (C=O), 168.4 (Cq) ppm; HRMS (ESI+) m/z calcd for C_24_H_27_N_5_O_2_ 418.2243, found 418.1865 [C_24_H_27_N_5_O_2_]+H]^+^.

##### 2.1.5.5. *N*-(4-Methyl-3-((4-methylpyridin-2-yl)amino)phenyl)-4-(morhpolinomethyl)benzamide (Compound 9)

Cream-white gummy solid, (This compound was purified by flash column chromatography using DCM/MeOH 7:1 as eluent, Rf: 0.60 (7:1 DCM/MeOH), yield 49.4%, m.p.155–157°C); IR (ATR) n_max_ 3292 (br) (NH), 2919 (m), 2852 (m), 1652 (s) (C=O), 1605 (vs), 1563 (m), 1524 (m), 1417 (w), 1261 (m), 1112 (vs), 1006 (vs), 865 (vs), 801 (vs) cm^−1^; ^1^H NMR (500 MHz, CDCl_3_): δ = 2.33 (s, 3H, CH_3_), 2,38 (s, 3H, CH_3_), 2.52 (t, J= 2.2 Hz, 4H, N-CH_2_), 3.65 (s, 2H, CH_2_), 3.98 (t, J= 2.2 Hz, 4H, O-CH_2_), 4.00 (brds, 1H, NH), 6.54 (d, J= 4.1 Hz, 1H, ArH), 6.90 (d, J= 2.8 Hz, 1H, ArH), 7.20 (d, J= 3.2 Hz, 1H, Ar-H), 7.48–7.53 (m, 3H, Ar-H), 7.88 (d, J= 4.2 Hz, 2H, Ar-H), 7.90 (s, 1H, ArH), 7.99 (brds, 1H, NH), 8.32 (d, J= 2.8 Hz, 1H, ArH) ppm; ^13^C NMR (125 MHz, CDCl_3_): δ = 22.3 (CH_3_), 25.7 (CH_3_), 42.4 (N-CH_2_), 55.92 (N-CH_2_), 62.4 (O-CH_2_), 63.9 (CH_2_), 68.5 (O-CH_2_), 109.0 (CAr), 110.3 (CAr), 119.4 (CAr), 123.9 (CAr), 125.0 (Cq), 126.9 (3xCAr), 129.5 (2xCAr), 130.8 (CAr), 133.6 (Cq), 133.8 (Cq), 136.5 (Cq), 156.0 (Cq), 165.4 (Cq), 165.5 (C=O), 168.4 (Cq) ppm; HRMS (ESI^+^) m/z calcd for [C_25_H_28_N_4_O_2_]+H^+^ 417.5234, found 417.5270 ([C_25_H_28_N_4_O_2_]+H)^+^.

##### 2.1.5.6. *N*-(4-Methyl-3-((4-methylpyridin-2-yl)amino)phenyl)-4-((4-(2,3,4-trimethoxybenzyl)piperazin-1-yl)methyl)benzamide (Compound 10)

Orange-brownish gummy solid, (This compound was purified by flash column chromatography using DCM/MeOH 7:1 as eluent, Rf: 0.54 (7:1 DCM/MeOH), yield 44.6%, m.p.87°C); IR (ATR) n_max_ 3295 (NH), 2936, 2811, 1652 (C=O), 1140, 1008 cm^−1^; ^1^H NMR (500 MHz, CDCl_3_): δ = 2.24 (s, 3H, CH_3_), 2.41 (s, 3H, CH_3_), 2.53 (brds, 8H, N-CH_2_), 3.45 (s, 2H, CH_2_), 3.54 (s, 2H, CH_2_), 3.85 (s, 3H, O-CH_3_), 3.86 (s, 3H, O-CH_3_), 3.88 (s, 3H, O-CH_3_), 6.49 (d, J= 5.0 Hz, 1H, Ar-H), 6.59 (d, J= 5.0 Hz, 1H, Ar-H), 6.62 (d, J= 8.5 Hz, 1H, Ar-H), 6.92 (brds, 1H, NH), 7.01 (d, J= 8.5 Hz, 1H, Ar-H), 7.16 (d, J=8.3 Hz, 1H Ar-H), 7.40 (d, J=8.1 Hz, 1H, Ar-H), 7.44 (d, J=7.3 Hz, 2H, Ar-H), 7.79 (d, J= 8.1 Hz, 2H, Ar-H), 8.03 (brds, 1H, NH), 8.26 (d, J= 5.0 Hz, 1H, Ar-H), 8.33 (d, J= 1.9 Hz, 1H, Ar-H) ppm; ^13^C NMR (125 MHz, CDCl_3_): δ = 20.6 (CH_3_), 24.1(CH_3_), 53.0 (2xN-CH_2_), 55.9 (OCH_3_), 56.3 (2xCH_2_), 60.3 (OCH_3_), 61.1 (OCH_3_), 62.5 (2xN-CH_2_) 106.9 (CAr), 109.8 (CAr), 112.3 (CAr), 112.7 (CAr), 114.9 (CAr), 123.5 (Cq), 125.3 (CAr), 127.0 (3xCAr), 129.3 (2xCAr), 130.8 (CAr), 134.0 (Cq), 136.5 (Cq), 138.0 (Cq), 142.2 (Cq), 142.4 (Cq), 150.2 (2xCq), 157.4 (CAr), 160.1 (Cq), 165.5 (Cq), 168.3 (C=O) ppm; HRMS (ESI+) m/z calcd for [C_35_H_41_N_5_O_4_]+H^+^ 596.7391, found 596.7520 ([C_35_H_41_N_5_O_4_]+H)^+^.

### 2.2. Cell Culture

#### 2.2.1. Chemicals and Instruments

K562, Jurkat, Molt-4, and Nalm-6 cell lines were cultured in RPMI-1640 medium (ECM2001, Euroclone), Reh cell line was cultured in DMEM medium (LM-D1111-Biosera) Hek293T cell line was cultured in IMDM medium (AL070A - HiMedia) supplemented with 10% heat-inactivated fetal bovine serum (F7524-Merck & Co., USA,) penicillin and streptomycin (P4333-Sigma Aldrich). K562, Jurkat and Molt-4 cell lines were seeded as 4x10^5^/ml, Reh and Nalm-6 cell lines were seeded 5x10^5^/ml, Hek293T cell line was seed as 2x10^5^/ml. All cells were incubated at 5% CO_2_ and 37 °C. All cell lines were kindly provided by Dr. M. Sayitoglu and Dr. Ö. Hatirnaz-Ng from Acibadem University.

##### 2.2.1.1. In vitro toxicity assay

MTT assay [37] was used to evaluate the growth inhibition percentage of the newly synthesized imatinib compounds against different types of leukemia cell lines (K562, Nalm-6, Molt4, Reh and Jurkat) and control cell line non-leukemia cell line (Hek293T).

MTT assay is a 3-day test. On the first day, cell count was performed in Bio Rad TC-20 device with the help of trypan blue, each cell line was prepared by diluting to the specified concentrations and seeded in a 96-well plate as 100μL to each well and incubated for 1 day.

Each compound was solved in DMSO, their stock solutions (33.3, 10, 5, 1 mM, and 300 μM) were prepared by diluting with the same solvent and they were stored under −20°C when they were not used.

On the second day, stock solutions were subsequently diluted to various concentrations (0.3–200 μM) with related medium prior to experiments. Specified concentrations of the compounds were seeded in a 96-well plate as 100μL to each well and incubated for 24 h.

After incubation (on the third day), to precipitate leukemia cells, the cell plate was centrifuged at 1500 rpm for 5 min. Then, 100 μL of supernatant was removed from each well carefully and 10 μl of MTT solution (5 mg/mL) was added to each well and incubated at 37 °C for 4 h. The 50 μl of supernatant was discarded from each well and 100 μl of DMSO was added to dissolve the formazan. Plates were shaken for 45 min to dissolve the dye. Then the optical density of each well was measured by using Thermo VarioSkan Flush Multimode Reader Quantum ST5-1100 at 570 nm wavelength.

Subsequently, viability rate of the cells at each concentration, as percentage, was determined by following formula (1):


$$\begin{array}{l} \text{Viability rate }(\%)=100-[({\text{OD}}_{\left(\text{control}\right)}-{\text{OD}}_{\left(\text{sample}\right)})/{\text{OD}}_{\left(\text{control}\right)}\times 100].\\ \text{OD}=\text{Optical Density} \end{array}$$

##### 2.2.1.2. Statistical Analyses for IC_50_

The IC_50_ values were calculated by using GraphPad Prism. All the data presented as the mean of 6 replicates. Results were analyzed and illustrated with Graph Pad Prism (version 5; GraphPad Software, San Diego, CA, USA). Statistical analysis was performed using dose-response inhibition, log(inhibitor) vs response-variable slope, least squares (ordinary) fit.

### 2.3. ABL1 Expression analysis

Raw data generated using Affymetrix Human Gene 1.0 ST Array were collected from GEO (Gene Expression Omnibus) using the accession numbers GSE139094 (Hek293T), GSE48558 (Jurkat, K562, Nalm-6 and Reh) and GSE26790 (Molt-4). The raw data were processed using the RMA (Robust Multi-array Average) method, which is part of the R package oligo [38] and the batch effect was removed using the limma package [39]. The Affymetrix Probe IDs were mapped to Ensembl IDs with the help of hugene10sttranscriptcluster.db annotation package in R. The normalized and log2-transformed expression values for the gene ABL1 were extracted using the Ensembl ID ENSG0000097007. To assess whether a difference exists in the expression level of ABL1 among the cell lines, we performed one-way ANOVA (analysis of variance) assuming unequal variances.

### 2.4. Molecular Docking

#### 2.4.1. Prediction of Target Protein

Putative targets for the newly synthesized imatinib analogs were predicted using SEA (Similarity Ensemble Approach, https://sea.bkslab.org/) online search tool provided by Shoichet Laboratory in the Department of Pharmaceutical Chemistry at the University of California [40]. For this, SMILES (Simplified Molecular-Input Line-Entry System) representations for the derivatives were generated and used as search keys to the SEA server that uses chemical similarity to find protein targets (see Table-S3).

#### 2.3.2. Crystal Structures and Docking Procedure

In order to assess which conformation of ABL1 and BRAF kinases our newly synthesized compounds bind to, we performed molecular docking simulations using 8 and 7 different crystal structures of kinase domains of wild type ABL1 and BRAF respectively. The properties of the structures used in this study together with the original ligands that are bound to the structures are given in Table S4 and Table S6.

We used the chemical toolbox Open Babel 2.4.1[41] in order to build the initial conformations of the compounds from their SMILES representations. The target protein structures (see Table S5) and compounds were prepared for docking using the AutoDock Tools version 1.5.6 [42]. AutoTors utility of AutoDock Tools was used for definition of the torsions of the compounds. All torsions except for amide and ring torsions were treated as flexible. Gasteiger atomic charges [43] were assigned to both the protein and the compounds. The nonpolar hydrogen atoms were merged while the polar hydrogen atoms were kept explicit.

Extensive docking simulations were performed using the program AutoDock 4.2 [42]. Grid maps were generated with 0.375Å spacing by the AutoGrid program. The grid center was chosen to coincide with the center of the original ligand in the crystal structure. Grid dimensions (70Å x 70Å x 70Å) that span the binding pocket in three dimensions were computed.

Standard Lamarckian genetic algorithm protocol was used with default settings, except for the number of energy evaluations and the number of independent runs, which were increased to obtain more reliable results. We started molecular docking simulations with 25 million energy evaluations for imatinib and its newly synthesized 6 analogs. We assessed the convergence of a docking simulation by performing clustering analysis of the resulting docking conformations where we used a root mean square deviation of 2 Å as cut-off. We assumed that the docking simulation was converged when 20% of the 100 independent runs resulted in the same binding conformation. When this condition was not met, we increased the maximum number of energy evaluations gradually. Due to their relatively high number of torsions (10 for compound 6 and 11 for compound 10), we performed additional docking simulations for the compounds 6 and 10 where we set the maximum number of energy evaluations to 30, 40, and 50 million. For the docking simulations of imatinib, Compound 5, Compound 7, Compound 8 and Compound 9; 25 million of maximum number of energy evaluations sufficed. However, for all reported results, a maximum number of energy evaluations of 40 million was used. The starting point of the ligand was generated randomly, in all docking simulations.

## 3. Results and Discussion

### 3.1. Chemistry

3-step synthesis of novel imatinib derivatives was performed by a substitution reaction (S_N_2) with Compound-3 [20–21] and various cyclic secondary amine compounds and then the obtained bromobenzamide intermediates (Compound 4a–c) were reacted with different hetaryl primary amines in the conditions of Buchwald Hardwing coupling reaction and Compound 5–10 were obtained at 40.0%–58.1% yields. The general procedure was outlined in Scheme (see Table S1). The structures of the target compounds 5–10 were elucidated by FT-IR, ^1^H NMR and ^13^C NMR, and HRMS (ESI^+^) analyses (see Figure S1–S43).

For preparation of compound 4a, 2-(1-piperazinyl)pyrimidine was used as an aryl piperazine group in synthesis of compound 5 and 7; because pyrimidine and its derivatives are found in nucleobases which composed DNA and RNA and they have broad spectrum of biological activities including anticancer activity [44]. For preparation of compound 4b, 1-(2,3,4-trimethoxybenzyl)piperazine dihydrochloride (called also as Trimetazidine dihydrochloride) was used as an aryl piperazine group, in synthesis of compounds 6 and 10. It was selected due to its antineoplastic [45] properties. For preparation of compound 4c, morpholine was used as cyclic secondary amine group, because morpholine moiety also plays critical role in several inhibition activities and used as anticancer agents [46–48].

The FTIR spectrum of compounds 5–10 showed characteristic NH and C=O peaks at 3200 and 1650cm^−1^, respectively. This situation confirms that there was a benzamide structure in the molecules.

When the results of the ^1^H NMR spectra were examined, the peaks of the −CH_3_ (methyl) groups on the pyridine, pyrimidine and benzene rings were observed individually between 2.28–2.39 ppm. The broad peak observed around 2.50 ppm belongs to the -N-CH_2_ that form the piperazine and morpholine rings. However, the peaks observed at 3.50 ppm indicate -N-CH_2_, the part of the piperazine and morpholine rings attached to the benzamide molecule. Also in the same circles indicates −CH_2_ where the trimethoxyphenyl ring is attached to the piperazine (compounds 6 and 10).

The peaks of −OCH_3_, which were substituents of the same compounds, were around 3.80–3.95 ppm. The two NH peaks in the molecules were observed between 3.48–4.02 and 6.75–6.92 ppm. Multiple peaks were appeared in the range 7.00–8.33 ppm due to aromatic hydrogen atoms.

In ^13^C NMR spectra, the peaks belong to aliphatic (−CH_3_, −CH_2_-) parts were observed between 20–65 ppm and the peaks of the aromatic parts were upward 100 ppm. Carbonyl peak of amide was seen around 165 ppm.

### 3.2. Characteristics of Newly Synthesized Compound According to Lipinski’s Rule of Five

Because oral using of pharmaceutical compounds is easier, the new molecules were evaluated by Lipinski Rule of 5, using Medchem Designer program and https://www.molinspiration.com, comparing with imatinib, (see Table 1) Compound-6 and 10 slightly exceed the rules due to MW > 500 g/mole and RB is higher than 10 for compound-10.

Note: Calculation of Molecular Properties and Bioactivity Score [online]. Website https://www.molinspiration.com/cgi-bin/properties. [accessed 10 May 10 2021]

### 3.3. In vitro anti-proliferative activity

**Figure 2:**
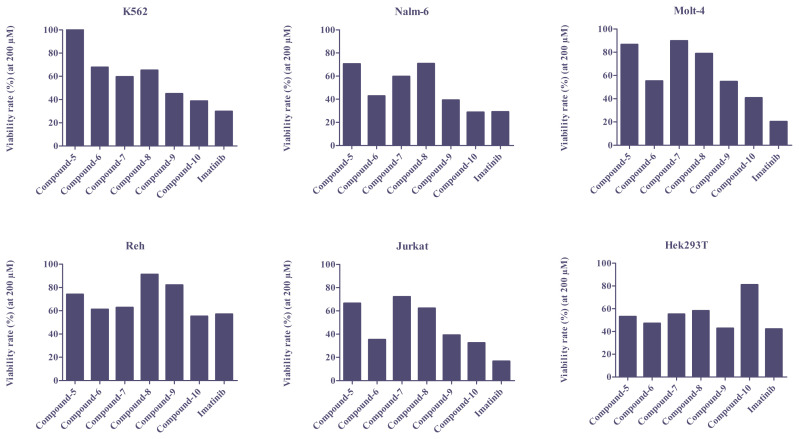
Viability rate of the compound 5–10 and imatinib (at 200 μM) in different cell lines.

To evaluate in-vitro anti-cancer activity of the newly synthesized compounds, they were tested by using the MTT assay in the BCR-ABL positive leukemic cell line (K562), BCR-ABL negative leukemic cell lines (Nalm-6, Molt-4, Reh and Jurkat), and non-leukemic human embryonic kidney tissue (Hek293T) cell line. All cells were incubated with various concentrations (between 0.3 and 200μM) of the derivatives for 24 h, and imatinib was used as the reference compound. The viability rate of each concentration was calculated by the formula stated in material and methods, the viability rate results for each cell were presented in Table S2 and Figure S44. Accordingly, after the test, only the compounds viability rate less than 50% were considered for calculation IC_50._ The corresponding results for IC_50_ values presented in Table 2. And viability rate (at 200 μM) graphics in different cell lines were shown in Figure 2.

In K562 cells, after 24 h incubation with the compounds, the lowest viability rate was observed with imatinib, around 30% (29.7%). Among the newly synthesized derivatives, only compound 10 (38.7%), and in less extent compound 9 (45.0%), were also able to achieve lower viability rate in the K562 cell line. Moreover, the IC_50_ value of compound 10 (35.04 μM) is lower compared to imatinib (78.37 μM) indicating K562 cell line is more sensitive to the newly synthesized derivative than the reference compound. The other derivatives were significant less effective in reducing the number of living cells compared to imatinib.

In BCR-ABL negative cell lines, a decrease in the number of viable cells was also observed with drug administration. In consistent with K562 cells, compound 6, 9, and 10 exhibited anti-proliferative activities in nearly all BCR-ABL negative cell lines. In Nalm-6 and Jurkat cell lines, 50% decrease was observed in the viable cells. Especially for Nalm-6, after incubation with compound-6 and compound-9 percentage of viable cells was calculated as 42.8% and 39.2%. The IC_50_ values were 47.96 μM and 1.639 μM respectively. The observed viability rate values of compound 10 and imatinib were very close to each other, compound-10-administered cells showed viability as 28.7% with IC_50_ of 28.73 μM and imatinib-administered cells showed viability as 29.0% with IC_50_ of 16.09 μM in Nalm6 cells. The effects of imatinib and its derivatives on Jurkat cells were similar to the results obtained with Nalm-6 cells. Again, the best decrease in the number of viable cells was obtained with imatinib (16.6% viability). In Molt-4 the reduction in the number of viable cells was moderate or low for all the compounds. Interestingly, only compound-10, showed less 50% viability on Molt-4 cells. Reh cells appear to be less sensitive to the newly synthesized derivatives. Up to about 60% viability was seen in Reh cells, including imatinib (see Figure S44).

In general, according to MTT assay results, the compounds with 2,3,4-trimethoxybenzyl in aryl piperazine group (R1, Scheme), (compound 6 and 10) exhibited high anticancer activity on all cell lines, except Reh. Whereas, instead of aryl piperazine ring, morpholine substituted analogs (compound 8 and 9) displayed somewhat relatively lower activity, and the analogs with pyrimidine ring in aryl piperazine group (compound 5 and 7) were characterized by very poor activity.

Also, the results show that there was not so much difference between 2-aminopyrimidine and 2-aminopyrazine heterocyclic ring system (R2, Scheme), (compound 5–7).

High growth inhibitory activity was also obtained for pyrimidine-substituted derivatives. And also, the pyridine linked analogs (compound 9–10) exhibited high anticancer activity than pyrimidine and pyrazine group.

### 3.4. ABL1 Expression analysis

Although imatinib was specifically designed as a drug for the BCR-ABL fusion gene product, our results showed that it was also effective in BCR-ABL negative cell lines. One explanation can be given that all the compounds, including imatinib mesylate, are targeted to the kinase domain of ABL protein. The ABL protein, encoded by the ABL1-gene, is a non-receptor tyrosine kinase, which is constitutively expressed in all cells. ABL protein is activated in response to several stimuli such as cell adhesion, cytokines, growth factors, DNA damage, and other signals. Activation of ABL protein will resulted in migration, cell proliferation, differentiation and apoptosis. Although the BCR-ABL negative cell lines do not express the fusion protein, they all expressed the endogenous ABL protein, which can be targeted also by the compounds. To support this, we have compared the gene expression of ABL1 in the cell lines by microarray data available in the literature. Indeed, we observed that ABL1 gene is highly expressed in these cell lines, with the highest in K562 cell line.

Secondly, other tyrosine kinase proteins with similar kinase domain as the ABL1 protein can also be targeted by the compounds as it is predicted with the SEA search tool.

**Figure 3:**
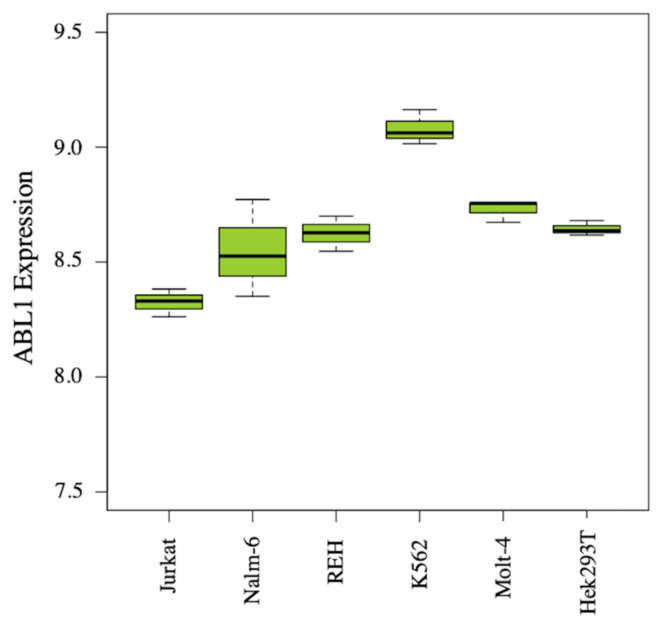
Boxplot of normalized and log2-transformed expression values for Abl1. The figure was generated using graphics package in R version 3.6.3.

Figure 3 depicts the expression level of the gene ABL1 in the cell lines. The mean expression level for ABL1 varies between 8.3 (Jurkat) and 9.1 (K562). Even though the one-way ANOVA results suggest significant difference (p-value < 0.01), the log2 fold change values (< 0.8) we obtained from binary comparisons support no difference in ABL1 expression level among the cell lines.

The comparable expression level of ABL1 in the studied cell lines may explain why the compounds 6, 9, and 10 show no cell line specific inhibitory effect.

### 3.5. Molecular docking

#### 3.5.1. Protein targets for novel imatinib analogs

SEA [40] search tool predicted several proteins as target for the novel imatinib analogs (see Table S4). We picked three most appropriate targets that are common to lists of imatinib and its newly synthesized analogs and decided to further assess them using molecular docking simulations. Among our 3 putative targets, which are Atypical chemokine receptor 3 (P25106), Serine/threonine-protein kinase BRAF (Uniprot ID: P155056) and BCR/ABL p210 fusion protein (A1Z199), Atypical chemokine receptor 3 does not have a crystal structure; hence, we could not perform molecular docking simulations for this protein.

BCR/ABL fusion protein has a constitutively activated ABL tyrosine kinase domain. Imatinib inhibits the catalytic activity of BCR/ABL by binding to an inactive conformation of the ABL kinase domain [49]. Since our newly synthesized compounds are analogs of imatinib, it is not surprising that ABL kinase is one of the putative targets for our compounds. Superposition of various structures reported, and molecular dynamic simulation studies performed show a great conformational flexibility in ABL protein kinase [49–51]. This conformational plasticity is the reason for the differences in inhibitor binding site, which has been exploited for inhibitor selectivity and affinity optimization. The conformation of the DFG motif has long been known for its effect on the binding pocket. Four main conformations have been reported for the highly conserved DFG motif. Those conformations can be listed as the active conformation, the DFG-out conformation, the DFG-flip conformation and the Src-like inactive conformation. We evaluated all crystal structures of wild-type human ABL kinase domain in the Protein Databank [52–53] and selected 8 of them for molecular docking analysis (see Table S5). These selected structures represent inactive DFG-out conformation [2HYY [51], 2E2B [54], 2HZ0 [51], 3CS9 [55] and 3UE4 [56]], Src-like inactive conformation (4CY8 [57]), intermediate DFG-flip conformation (2HZI [51]) and active conformation (2HZ4 [51]).

Table 3 summarizes the results of the docking simulations of imatinib and its 6 newly synthesized analogs to the 8 different conformations of wild-type human ABL kinase domain. We assessed the reliability of docking results using the percentage of independent runs that converge to the same binding conformation. We assumed a docking simulation result reliable when at least 20% of the independent runs resulted in this particular binding conformation (see Table 3).

**Figure 4:**
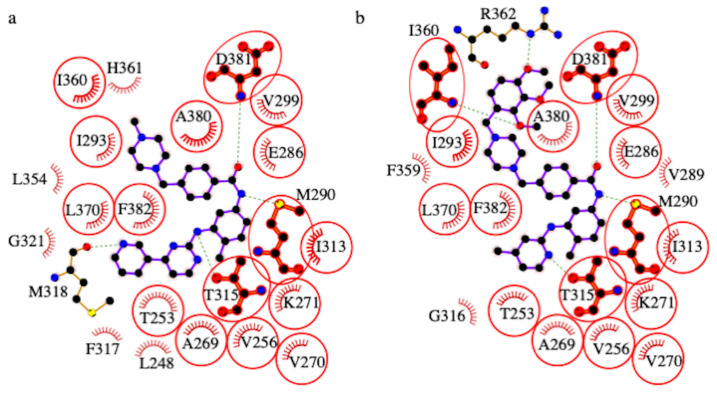
Detailed interactions of ABL1 kinase domain with imatinib (a) and Compound 10 (b). The common contact residues are highlighted with red circles. Hydrogen bonds are indicated by dashed lines, while the hydrophobic interactions are represented by an arc with spokes. The figure was generated using LigPlot+ v.2.2 [58].

This assumption is based on the re-docking calculations that we performed where we docked the original ligands to the protein conformations found in the crystal structures. The sizes of the clusters that we obtained from the re-docking calculations vary between 20 and 100 conformations (see Table S5 and Table S7). Our results presented in Table 3 suggest that imatinib binds to different conformations of ABL however with varying free energies of binding (9.6 – 14.7 kcal/mol). According to the free energies given in the table, imatinib prefers DFG-out conformations of the ABL kinase domain, which is also well reported in the literature [49]. The analog compounds 5, 7, 8, 9 and 10 have a similar tendency with imatinib. However, compound 6 seems to prefer the intermediate conformation where the DFG motif adopts a flipped conformation (see Table 3). Among all the newly synthesized analogs compound 10 has the most favorable free energy of binding (−14.2 kcal/mol) that is closest to the free energy of binding for imatinib (−14.7 kcal/mol). Evaluating molecular docking and MTT assay results together we decided to compare the interactions of imatinib and compound 10 with ABL kinase domain in details. As provided in Figure 4, two molecules adopt overall a similar binding mode despite some local differences. The common rings (the methylbenzene, the benzamide and the N-methylpiperazine rings) between two molecules adopt exactly the same conformation and contribute to the same type of interactions. The overlapping in binding modes is also true for Compound 5, 7, and 8 (see Figure S45, S47 and S48) and partially true for Compound 9 (see Figure S49). However, compared to imatinib, compound 6 adopts a very distinct binding mode (see Figure S46).

Similar to ABL kinase, for BRAF kinase different conformational states reported in literature as well; inactive DFG-out conformation [1UWH[59], 4KSP[60] and 4JVG[61]], Src-like inactive conformation [3C4C[62] and 5CSW[63]] and active conformation [2FB8[64] and 3D4Q[65]]. The results of molecular docking to wild type human BRAF kinase domain is tabulated in Table 4. As seen in the table, the new imatinib analogs have lower affinities for BRAF kinase compared to ABL kinase.

## 4. Conclusion

The anti-proliferative activities in vitro showed that compound 10 gives close results to imatinib. Although imatinib was specifically designed as a drug for the BCR-ABL fusion gene, our results showed that it was also effective for Jurkat and Nalm-6 cells, which are BCR-ABL negative cells. We also found that Compound 6 and 9 were relatively effective in Nalm-6 and Jurkat cells.

Except for compound 6, the newly synthesized imatinib analogs prefer a DGF-out conformation of ABL kinase. Among all newly synthesized analogs, compound 10 has the highest affinity for ABL kinase domain which is comparable to the affinity of imatinib for the ABL kinase domain. Analysis of molecular interactions revealed similarities between binding patterns of compound 10 and imatinib. Despite predicted to be a potential target, the new imatinib analogs have lower affinities for BRAF kinase compared to ABL kinase.

To sum up, this study introduces a novel successful design for imatinib derivatives as the potential antitumor agents. These compounds possess a simple molecular structure and are easy to synthesize, which makes them very attractive for further exploration as kinase inhibitors with application in cancer.

## Supplementary Information

**Table:** 

Table of Contents	Page
Table S1. New imatinib derivatives list	S4–S5
Figure S1. IR spectrum of Compound 3	S6
Figure S2. ^1^H-NMR spectrum of Compound 3	S6
Figure S3. APT spectrum of Compound 3	S7
Figure S4. IR spectrum of Compound 4a	S8
Figure S5. ^1^H-NMR spectrum of Compound 4a	S8
Figure S6. APT spectrum of Compound 4a	S9
Figure S7. HRMS Spectrum of Compound 4a	S9
Figure S8. IR spectrum of Compound 4b	S10
Figure S9. ^1^H-NMR spectrum of Compound 4b	S10
Figure S10. ^1^H-NMR spectrum of Compound 4b	S11
Figure S11. ^1^H-NMR spectrum of Compound 4b	S11
Figure S12. APT spectrum of Compound 4b	S12
Figure S13. HRMS Spectrum of Compound 4b	S12
Figure S14. IR spectrum of Compound 4c	S13
Figure S15. ^1^H-NMR spectrum of Compound 4c	S14
Figure S16. APT spectrum of Compound 4c	S14
Figure S17. HRMS Spectrum of Compound 4c	S15
Figure S18. IR spectrum of Compound 5	S16
Figure S19. ^1^H-NMR spectrum of Compound 5	S16
Figure S20. APT spectrum of Compound 5	S17
Figure S21. HRMS Spectrum of Compound 5	S17
Figure S22. IR spectrum of Compound 6	S18
Figure S23. ^1^H-NMR spectrum of Compound 6	S18
Figure S24. ^1^H-NMR spectrum of Compound 6	S19
Figure S25. ^1^H-NMR spectrum of Compound 6	S19
Figure S26. APT spectrum of Compound 6	S20
Figure S27. HRMS Spectrum of Compound 6	S20
Figure S28. IR spectrum of Compound 7	S21
Figure S29. ^1^H-NMR spectrum of Compound 7	S21
Figure S30. APT spectrum of Compound 7	S22
Figure S31. HRMS Spectrum of Compound 7	S22
Figure S32. IR spectrum of Compound 8	S23
Figure S33. ^1^H-NMR spectrum of Compound 8	S24
Figure S34. APT spectrum of Compound 8	S24
Figure S35. HRMS Spectrum of Compound 8	S25
Figure S36. IR spectrum of Compound 9	S26
Figure S37. ^1^H-NMR spectrum of Compound 9	S26
Figure S38. APT spectrum of Compound 9	S27
Figure S39. HRMS Spectrum of Compound 9	S27
Figure S40. IR spectrum of Compound 10	S28
Figure S41. ^1^H-NMR spectrum of Compound 10	S28
Figure S42. APT spectrum of Compound 10	S29
Figure S43. HRMS Spectrum of Compound 10	S29
Table S2. Concentration (μM)-viability rate ( %) values according to MTT assay in different cell lines.	S30–S32
Figure S44. Comparision between imatinib and compound-6–9–10 in all cell-lines	S33
Table S3. Values of synthesized molecules related to target proteins	S34
Table S4. Cristal structures of ABL1	S35
Table S5. Redocking the original ligands to the crystal structures of wild type ABL1	S35
Table S6. Cristal structures of BRAF	S36
Table S7. Redocking of the original ligands to the crystal structures of BRAF	S37
Figure S45. Comparison of ABL-imatinib (background) and ABL-Compound 5 (foreground) interactions. Imatinib and its contact residues are depicted in gray, while the common contact residues are marked with red circles.	S38
Figure S46. Comparison of ABL-imatinib (background) and ABL-Compound 6 (foreground) interactions. Imatinib and its contact residues are depicted in gray, while the common contact residues are marked with red circles.	S39
Figure S47. Comparison of ABL-imatinib (background) and ABL-Compound 7 (foreground) interactions. Imatinib and its contact residues are depicted in gray, while the common contact residues are marked with red circles.	S40
Figure S48. Comparison of ABL-imatinib (background) and ABL-Compound 8 (foreground) interactions. Imatinib and its contact residues are depicted in gray, while the common contact residues are marked with red circles.	S41
Figure S49. Comparison of ABL-imatinib (background) and ABL-Compound 9 (foreground) interactions. Imatinib and its contact residues are depicted in gray, while the common contact residues are marked with red circles.	S42

**Table S1:** New imatinib derivatives list

Entry	Compound Name	Compound Structure
**3**	*N*-(3-Bromo-4-methylphenyl)-4-(chloromethyl)benzamide	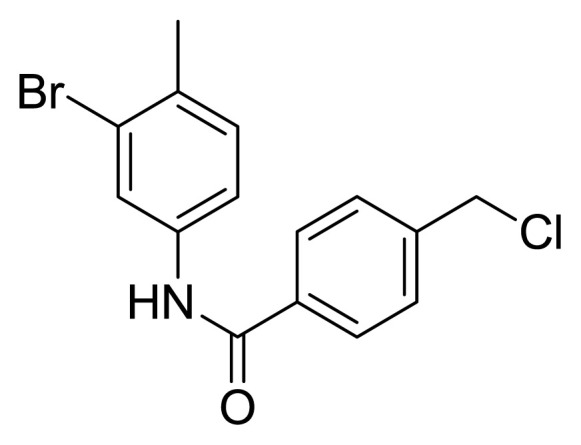
**4a**	*N*-(3-Bromo-4-methylphenyl)-4-((4-(pyrimidin-2-yl)piperazin-1-yl)methyl)benzamide	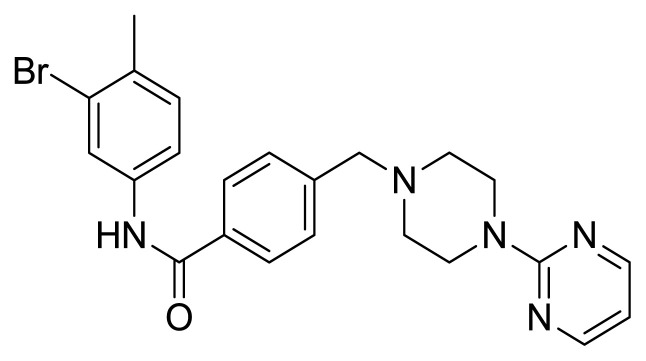
**4b**	*N*-(3-Bromo-4-metylphenyl)-4-((4-(2,3,4-trimethoxybenzyl)piperazin-1-yl)methyl)benzamide	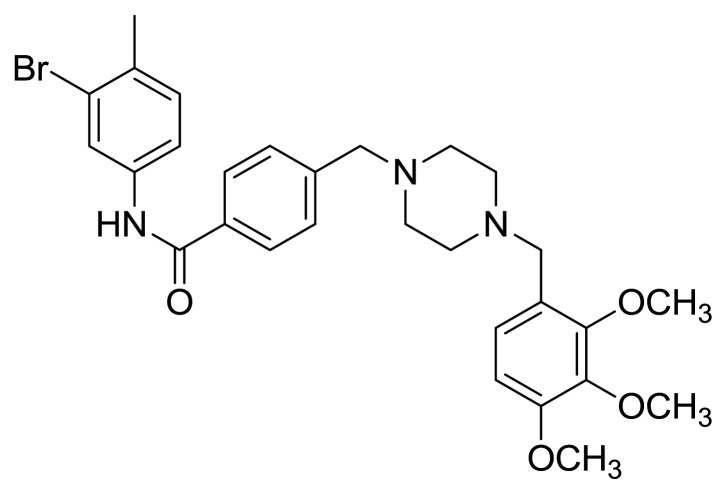
**4c**	*N*-(3-Bromo-4-methylphenyl)-4-(morpholinomethyl)benzamide	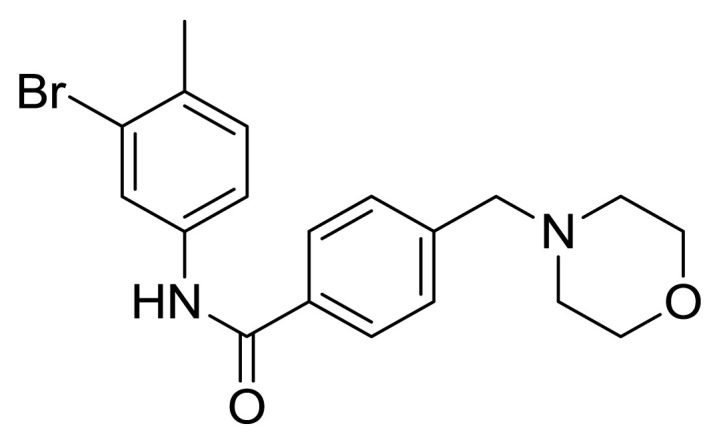
**5**	*N*-(4-Methyl-3-((4-methylpyrimidin-2-yl)amino)phenyl)-4-((4-(pyrimidin-2-yl)piperazin-1-yl)methyl)benzamide	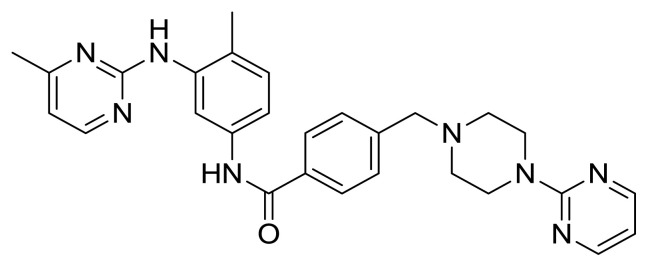
**6**	*N*-(4-Methyl-3-((4-methylpyrimidin-2-yl)amino)phenyl)-4-((4-(2,3,4-trimethoxyphenyl)piperazin-1-yl)methyl)benzamide	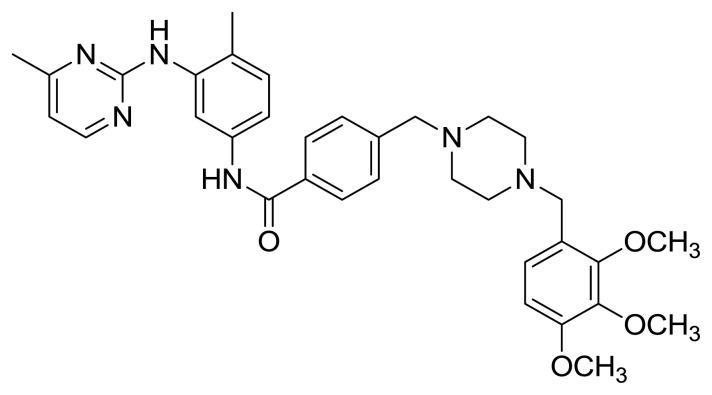
**7**	*N*-(4-Methyl-3-(pyrazin-2-ylamino)phenyl)-4-((4-(pyrimidin-2-yl)piperazin-1-yl)methyl)benzamide	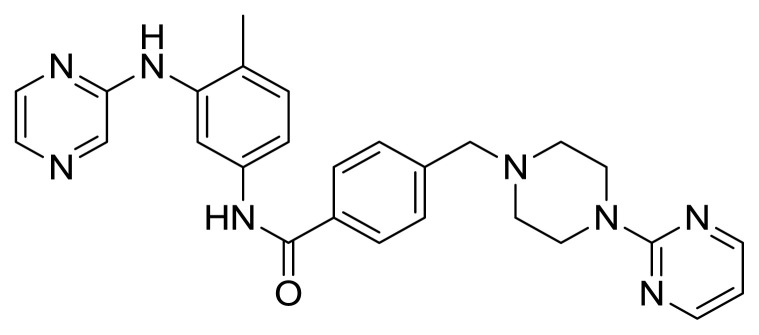
**8**	*N*-(4-Methyl-3-((4-methylpyrimidin-2-yl)amino)phenyl)-4-(morhpolinomethyl)benzamide	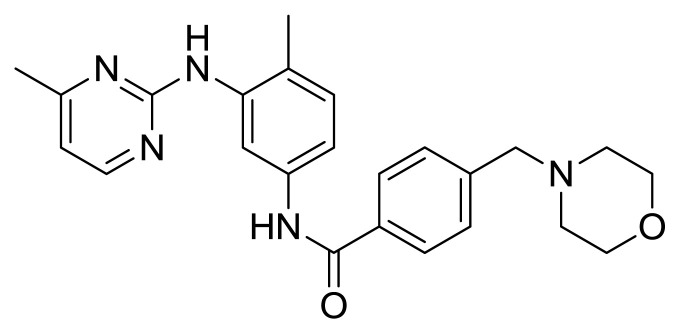
**9**	*N*-(4-Methyl-3-((4-methylpyridin-2-yl)amino)phenyl)-4-(morhpolinomethyl)benzamide	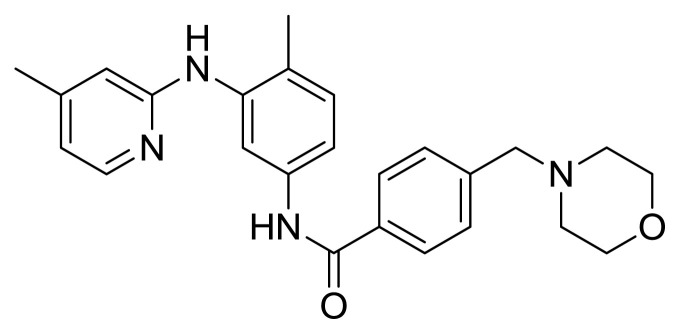
**10**	*N*-(4-Methyl-3-((4-methylpyridin-2-yl)amino)phenyl)-4-((4-(2,3,4-trimethoxybenzyl)piperazin-1-yl)methyl)benzamide	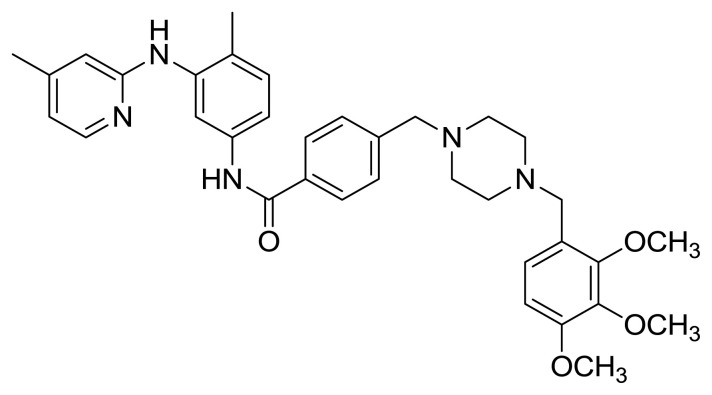

### Compound 3

Figure S1 IR spectrum of Compound 3.

Figure S2 ^1^H-NMR spectrum of Compound 3 (Reference-13).

Figure S3 ^13^C-NMR spectrum of Compound 3 (Reference-13).

### Compound 4a

Figure S4 IR spectrum of Compound 4a.

Figure S5 ^1^H-NMR spectrum of Compound 4a.

Figure S6 APT spectrum of Compound 4a.

Figure S7 HRMS Spectrum of Compound 4a.

### Compound 4b

Figure S8 IR spectrum of Compound 4b.

Figure S9 ^1^H-NMR spectrum of Compound 4b.

Figure S10–S11 1H-NMR spectrum of Compound 4b.

Figure S12 APT spectrum of Compound 4b.

Figure S13 HRMS Spectrum of Compound 4b.

### Compound 4c

Figure S14 IR Spectrum of Compound 4c.

Figure S15 1H-NMR spectrum of Compound 4c.

Figure S16 APT spectrum of Compound 4c.

Figure S17 HRMS Spectrum of Compound 4c.

### Compound 5

Figure S18 IR Spectrum of Compound 5.

Figure S19 1H-NMR spectrum of Compound 5.

Figure S20 APT spectrum of Compound 5.

Figure S21 HRMS Spectrum of Compound 5.

### Compound 6

Figure S22 IR spectrum of Compound 6.

Figure S23–S24–S25 1H-NMR spectrum of Compound 6.

Figure S26 APT spectrum of Compound 6.

Figure S27 HRMS Spectrum of Compound 6.

### Compound 7

Figure S28 IR spectrum of Compound 7.

Figure S29 1H-NMR spectrum of Compound 7.

Figure S30 APT spectrum of Compound 7.

Figure S31 HRMS Spectrum of Compound 7.

### Compound 8

Figure S32 IR spectrum of Compound 8.

Figure S33 1H-NMR spectrum of Compound 8.

Figure S34 APT spectrum of Compound 8.

Figure S35 HRMS Spectrum of Compound 8.

### Compound 9

Figure S36 IR spectrum of Compound 9.

Figure S37 1H-NMR spectrum of Compound 9.

Figure S38 APT spectrum of Compound 9.

Figure S39 HRMS Spectrum of Compound 9.

### Compound 10

Figure S40 IR spectrum of Compound 10.

Figure S41 1H-NMR spectrum of Compound 10.

Figure S42 APT spectrum of Compound 10.

Figure S43 HRMS Spectrum of Compound 10.

**Table S2:** Concentration (μM)-viability rate ( %) values according to MTT assay in different cell lines.

For K562														

Concentration (μM)-Viability rate ( %)														

Compd	0.3	0.5	1	3	5	10	15	20	30	50	70	100	150	200
5	-	-	103.1 ± 0.07	98.7 ± 0.08	99.7 ± 0.06	97.0 ± 0.05	99.5 ± 0.12	106.5 ± 0.04	102.7 ± 0.05	101.6 ± 0.12	100.1± 0.08	104.6 ± 0.10	104.3 ± 0.10	102.1 ±0.14
6	92.5 ± 0.11	91.8 ± 0.06	91.8 ± 0.04	88.7 ± 0.05	87.6 ± 0.05	87.3 ± 0.11	87.3 ± 0.10	86.5 ± 0.07	85.5 ± 0.10	78.1 ± 0.10	68.5 ± 0.05	67.5 ± 0.03	67.3 ± 0.02	67.2 ± 0.03
7	84.6 ± 0.15	84.5 ± 0.08	84.4 ± 0.08	84.5 ± 0.10	82.9 ± 0.12	81.6 ± 0.05	80.1 ± 0.06	64.0 ± 0.07	63.9 ± 0.04	62.9 ± 0.06	62.3 ± 0.07	62.2 ± 0.05	61.6 ± 0.05	59.5 ± 0.07
8	86.2 ± 0.09	85.3 ± 0.03	84.7 ± 0.05	84.7 ± 0.06	76.8 ± 0.11	76.7 ± 0.05	76.7 ± 0.08	76.6 ± 0.04	75.6 ± 0.09	72.5 ± 0.07	72.6 ± 0.08	66.4 ± 0.02	65.3 ± 0.06	65.2 ± 0.06
9	79.9 ± 0.09	78.5 ± 0.03	77.3 ± 0.08	77.0 ± 0.04	75.4 ± 0.09	73.2 ± 0.08	72.0 ± 0.06	69.1 ± 0.03	69.7 ± 0.07	61.2 ± 0.08	58.1 ± 0.10	51.3 ± 0.06	49.7 ± 0.05	45.0 ± 0.08
10	104.1 ± 0.07	103.4 ± 0.06	98.9 ± 0.05	98.7 ± 0.04	98.6 ± 0.06	98.9 ± 0.09	98.6 ± 0.08	79.2 ± 0.06	78.9 ± 0.10	56.1 ± 0.03	38.5 ± 0.02	38.7 ± 0.01	38.9 ± 0.01	38.7 ± 0.01
imatinib	98.0 ± 0.03	97.4 ± 0.06	95.4 ± 0.07	95.3 ± 0.05	95.4 ± 0.09	94.8 ± 0.09	88.5 ± 0.10	87.0 ± 0.04	72.8 ± 0.06	60.8 ± 0.10	60.0 ± 0.07	51.3 ± 0.05	35.1 ± 0.01	29.7 ± 0.01

For Nalm-6														

Concentration (μM)-Viability rate ( %)														

Compd	0.3	0.5	1	3	5	10	15	20	30	50	70	100	150	200
5	-	-	95.8 ± 0.01	95.0 ± 0.02	92.4 ± 0.03	90.1 ± 0.02	84.1 ± 0.01	80.1 ± 0.02	78.6 ± 0.01	74.1 ± 0.01	71.9 ± 0.01	70.3 ± 0.01	70.2 ±0.01	70.5 ±0.01
6	100.7 ± 0.01	100.5 ± 0.02	100.1 ± 0.03	100.0 ± 0.04	98.8 ± 0.04	98.0 ± 0.02	95.6 ± 0.02	82.6 ± 0.02	71.6 ± 0.01	55.2 ± 0.01	43.5 ± 0.01	42.6 ± 0.01	42.9 ± 0.01	42.8 ± 0.02
7	97.9 ± 0.06	95.1 ± 0.03	95.1 ± 0.03	96.0 ± 0.03	95.6 ± 0.02	95.1 ± 0.02	95.9 ± 0.03	96.4 ± 0.03	96.5 ± 0.05	72.3 ± 0.02	71.8 ± 0.02	71.3 ± 0.02	70.2 ± 0.01	59.7 ± 0.02
8	102.8 ± 0.04	101.8 ± 0.04	102.7 ± 0.02	99.6 ± 0.04	102.5 ± 0.02	100.6 ± 0.03	100.0 ± 0.03	94.2 ± 0.07	95.9 ± 0.07	91.9 ± 0.06	87.7 ± 0.02	87.4 ± 0.02	81.3 ± 0.02	70.8 ± 0.01
9	86.4 ± 0.05	85.2 ± 0.05	72.7 ± 0.04	64.5 ± 0.02	62.6 ± 0.04	53.2 ± 0.03	50.1 ± 0.03	49.2 ± 0.01	48.1 ± 0.01	43.9 ± 0.01	42.8 ± 0.01	42.5 ± 0.01	42.5 ± 0.01	39.2 ± 0.01
10	92.7 ± 0.08	88.6 ± 0.06	87.3 ± 0.07	86.5 ± 0.02	85.5 ± 0.06	84.8 ± 0.08	82.2 ± 0.08	71.9 ± 0.07	54.2 ± 0.07	29.8 ± 0.01	28.8 ± 0.01	28.7 ± 0.01	28.5 ± 0.01	28.7 ± 0.01
imatinib	91.0 ± 0.08	90.7 ± 0.06	90.1 ± 0.09	84.7 ± 0.09	84.5 ± 0.05	72.9 ± 0.03	57.1 ± 0.02	52.7 ± 0.01	50.7 ± 0.03	39.7 ± 0.01	32.5 ± 0.05	32.2 ± 0.02	32.5 ± 0.01	29.0 ± 0.02

For Molt-4														

Concentration (μM)-Viability rate ( %)														

Compd	0.3	0.5	1	3	5	10	15	20	30	50	70	100	150	200
5	-	-	95.2 ± 0.08	84.6 ± 0.04	85.1 ± 0.11	84.8 ± 0.03	84.9 ± 0.11	85.8 ± 0.03	85.3 ± 0.06	86.1 ± 0.11	84.6 ± 0.05	83.5 ± 0.12	87.1 ±0.09	86.6 ±0.08
6	84.4 ± 0.14	79.7 ± 0.10	78.7 ± 0.09	79.7 ± 0.18	80.5 ± 0.16	81.8 ± 0.07	79.2 ± 0.15	76.0 ± 0.05	75.4 ± 0.08	73.5 ± 0.04	68.9 ± 0.06	61.7 ± 0.02	59.9 ± 0.05	55.2 ± 0.04
7	93.3 ± 0.16	93.8 ± 0.08	89.8 ± 0.07	89.6 ± 0.12	89.7 ± 0.08	89.5 ± 0.06	90.3 ± 0.04	90.2 ± 0.06	90.9 ± 0.06	92.2 ± 0.05	90.6 ± 0.06	85.5 ± 0.06	90.4 ± 0.04	89.8 ± 0.07
8	98.5 ± 0.09	98.5 ± 0.11	95.4 ± 0.11	89.2 ± 0.07	90.7 ± 0.07	90.4 ± 0.07	90.8 ± 0.05	88.3 ± 0.04	85.8 ± 0.04	83.6 ± 0.02	82.6 ± 0.09	81.5 ± 0.09	78.9 ± 0.05	78.8 ± 0.07
9	93.2 ± 0.17	87.8 ± 0.05	78.4 ± 0.11	72.7 ± 0.04	71.7 ± 0.07	69.3 ± 0.04	68.9 ± 0.05	63.5 ± 0.04	62.0 ± 0.03	61.5 ± 0.07	59.8 ± 0.05	59.5 ± 0.08	58.6 ± 0.06	54.7 ± 0.03
10	87.9 ± 0.03	86.8 ± 0.02	87.7 ± 0.01	86.5 ± 0.02	86.2 ± 0.08	89.1 ± 0.03	88.1 ± 0.04	77.0 ± 0.04	65.3 ± 0.03	52.1 ± 0.03	42.8 ± 0.01	40.4 ± 0.01	40.0 ± 0.01	40.7 ± 0.01
imatinib	75.6 ± 0.18	74.9 ± 0.18	75.1 ± 0.19	73.8 ± 0.17	72.7 ± 0.25	70.7 ± 0.21	70.2 ± 0.10	63.4 ± 0.10	63.3 ± 0.06	55.5 ± 0.05	52.1 ± 0.10	41.2 ± 0.06	29.8 ± 0.04	20.3 ± 0.02

For REH														

Concentration (μM)-Viability rate ( %)														

Compd	0.3	0.5	1	3	5	10	15	20	30	50	70	100	150	200
5	-	-	96.6 ± 0.01	95.8 ± 0.05	95.7 ± 0.03	95.1 ± 0.04	94.9 ± 0.04	90.2 ± 0.03	87.9 ± 0.04	85.5 ± 0.05	81.4± 0.01	79.1 ± 0.01	79.1 ± 0.02	74. ±0.01
6	109.5 ± 0.02	105.2 ± 0.02	106.8 ± 0.01	107.9 ± 0.02	108.2 ± 0.01	106.9 ± 0.02	106.5 ± 0.02	97.7 ± 0.02	88.6 ± 0.03	85.6 ± 0.02	65.3 ± 0.02	62.2 ± 0.01	61.3 ± 0.01	61.1 ± 0.02
7	110.0 ± 0.02	109.8 ± 0.02	108.7 ± 0.02	103.4 ± 0.02	108.8 ± 0.02	106.5 ± 0.05	100.2 ± 0.02	95.5 ± 0.06	85.4 ± 0.02	66.7 ± 0.02	66.2 ± 0.03	67.7 ± 0.03	66.8 ± 0.02	62.8 ± 0.02
8	100.8 ± 0.03	102.4 ± 0.02	102.0 ± 0.01	104.8 ± 0.02	103.5 ± 0.01	103.1 ± 0.01	100.3 ± 0.01	100.6 ± 0.02	102.4 ± 0.04	99.4 ± 0.02	94.2 ± 0.03	91.1 ± 0.01	91.1 ± 0.02	91.2 ± 0.04
9	103.8 ± 0.01	101.3 ± 0.03	100.4 ± 0.01	100.5 ± 0.02	101.8 ± 0.03	103.4 ± 0.02	100.2 ± 0.02	99.0 ± 0.02	91.4 ± 0.01	88.2 ± 0.02	84.7 ± 0.03	87.1 ± 0.02	85.0 ± 0.01	82.0 ± 0.02
10	93.4 ± 0.02	92.4 ± 0.02	92.4 ± 0.01	92.6 ± 0.02	92.8 ± 0.02	92.3 ± 0.03	92.6 ± 0.02	91.8 ± 0.01	65.6 ± 0.02	59.5 ± 0.03	59.1 ± 0.02	59.6 ± 0.03	57.4 ± 0.03	55.2 ± 0.03
imatinib	97.6 ± 0.03	96.8 ± 0.03	96.4 ± 0.01	95.5 ± 0.02	94.5 ± 0.03	91.3 ± 0.05	82.4 ± 0.02	78.6 ± 0.02	70.9 ± 0.03	63.5 ± 0.04	62.9 ± 0.03	61.2 ± 0.01	60.3 ± 0.03	57.0 ± 0.02

For Jurkat														

Concentration (μM)-Viability rate ( %)														

Compd	0.3	0.5	1	3	5	10	15	20	30	50	70	100	150	200
5	-	-	90.7.1 ± 0.10	90.1 ± 0.11	89.7 ± 0.09	89.6 ± 0.15	88.8 ± 0.09	88.8 ± 0.08	88.1 ± 0.10	83.4 ± 0.12	82.5± 0.07	80.8 ± 0.13	78.7 ± 0.12	66.5 ±0.05
6	-	-	89.6.1 ± 0.02	79.1 ± 0.02	69.8 ± 0.02	69.7 ± 0.05	69.2 ± 0.02	68.9 ± 0.01	68.8 ± 0.03	59.1 ± 0.03	47.8 ± 0.01	44.0 ± 0.01	36.4 ± 0.01	35.3 ± 0.01
7	77.4 ± 0.09	77.3 ± 0.07	75.7 ± 0.03	75.5 ± 0.05	75.6 ± 0.03	74.6 ± 0.06	74.2 ± 0.05	74.0 ± 0.06	73.2 ± 0.07	72.8 ± 0.03	72.8 ± 0.03	72.7 ± 0.04	72.4 ± 0.09	72.1 ± 0.06
8	81.7 ± 0.08	78.7 ± 0.03	75.8 ± 0.04	76.8 ± 0.04	78.8 ± 0.02	78.5 ± 0.05	77.6 ± 0.05	77.6 ± 0.04	66.1 ± 0.07	65.4 ± 0.03	65.3 ± 0.04	63.0 ± 0.02	62.4 ± 0.08	62.3 ± 0.05
9	82.3 ± 0.05	82.0 ± 0.06	82.0 ± 0.05	81.7 ± 0.05	83.0 ± 0.04	81.9 ± 0.02	80.4 ± 0.02	79.0 ± 0.04	72.9 ± 0.07	68.1 ± 0.01	65.1 ± 0.02	49.6 ± 0.07	46.9 ± 0.05	39.1 ± 0.04
10	73.0 ± 0.10	71.7 ± 0.10	70.9 ± 0.08	69.4 ± 0.03	68.7 ± 0.05	68.1 ± 0.09	67.5 ± 0.07	64.1 ± 0.15	61.7 ± 0.07	61.4 ± 0.05	50.5 ± 0.02	38.1 ± 0.20	35.9 ± 0.12	32.5 ± 0.03
imatinib	68.4 ± 0.12	63.9 ± 0.03	61.5 ± 0.07	61.3 ± 0.05	60.7 ± 0.08	60.6 ± 0.14	57.9 ± 0.05	46.8 ± 0.05	34.5 ± 0.04	26.2 ± 0.05	17.8 ± 0.02	17.5 ± 0.01	17.3 ± 0.01	16.6 ± 0.01

For Hek293T														

Concentration (μM)-Viability rate ( %)														

Compd	0.3	0.5	1	3	5	10	15	20	30	50	70	100	150	200
5	-	-	68.7.1 ± 0.04	66.5 ± 0.11	61.0 ± 0.13	59.4 ± 0.08	56.6 ± 0.10	56.2 ± 0.03	55.8 ± 0.05	54.1 ± 0.04	52.7± 0.03	50.5 ± 0.02	50.4 ± 0.02	50.0 ±0.04
6	91.1 ± 0.15	90.8 ± 0.14	87.0± 0.15	81.4 ± 0.09	78.5 ± 0.14	78.2 ± 0.02	70.6 ± 0.04	65.5 ± 0.10	57.4 ± 0.09	50.7 ± 0.05	48.8 ± 0.02	47.9 ± 0.01	47.7 ± 0.02	47.0 ± 0.02
7	87.7 ± 0.06	87.6 ± 0.07	87.0 ± 0.08	84.6 ± 0.05	81.0 ± 0.09	80.5 ± 0.06	80.3 ± 0.04	76.0 ± 0.03	72.1 ± 0.02	64.5 ± 0.02	64.2 ± 0.02	63.5 ± 0.07	60.8 ± 0.03	55.1 ± 0.02
8	95.7 ± 0.03	94.8 ± 0.07	94.3 ± 0.06	92.9 ± 0.04	91.2 ± 0.09	90.8 ± 0.05	90.1 ± 0.07	90.0 ± 0.04	84.9 ± 0.04	81.3 ± 0.02	66.9 ± 0.02	66.4 ± 0.03	59.4 ± 0.01	58.2 ± 0.01
9	91.7 ± 0.07	89.8 ± 0.07	89.5 ± 0.05	87.4 ± 0.07	86.2 ± 0.03	79.3 ± 0.05	78.3 ± 0.03	76.5 ± 0.05	68.9 ± 0.03	62.2 ± 0.02	56.2 ± 0.04	54.8 ± 0.02	46.9 ± 0.02	42.8 ± 0.02
10	82.8 ± 0.02	78.2 ± 0.01	78.1 ± 0.01	80.40± 0.02	79.1 ± 0.01	80.2 ± 0.02	81.4 ± 0.01	80.8 ± 0.01	82.2 ± 0.03	79.2 ± 0.03	80.2 ± 0.02	80.7 ± 0.01	80.0 ± 0.01	81.0 ± 0.02
imatinib	83.4 ± 0.02	78.6 ± 0.04	77.7 ± 0.03	73.1 ± 0.03	71.5 ± 0.02	67.9 ± 0.01	59.6 ± 0.01	55.7 ± 0.03	53.5 ± 0.01	48.5 ± 0.05	42.9 ± 0.03	42.7 ± 0.03	42.5 ± 0.02	42.1 ± 0.01

Six replicates were performed for all concentrations.

Figure S44 Comparision between imatinib and compound-6–9–10 in all cell-lines.

**Table S3:** Values of synthesized molecules related to target proteins.

COMPD	TARGET NAME	DESCRIPTION	P-VALUE	MAX TC
**5**	ACKR3	Atypical chemokine receptor 3	2.746e-153	0.38
	BRAF		6.849e-151	0.64
	TCF7L2	Serine/threonine-protein kinase B-raf	1.228e-97	0.44
	BCR/ABL fusion	Transcription factor 7-like 2	2.331e-96	0.64
	CTNNB1	BCR/ABL p210 fusion protein	3.714e-90	0.44
		Catenin beta-1		
**6**	BCR/ABL fusion	BCR/ABL p210 fusion protein	3.407e-103	0.57
	ACKR3	Atypical chemokine receptor 3	3.763e-98	0.38
	CPT1B	Carnitine O-palmitoyltransferase 1, muscle isoform	2.844e-97	0.37
	BRAF	Serine/threonine-protein kinase B-raf	1.618e-83	0.57
	CPT1A	Carnitine O-palmitoyltransferase 1, liver isoform	4.802e-70	0.37
**7**	BRAF	Serine/threonine-protein kinase B-raf	2.562e-159	0.58
	ACKR3	Atypical chemokine receptor 3	8.059e-152	0.39
	BCR/ABL fusion	BCR/ABL p210 fusion protein	3.446e-103	0.58
	SLC6A7	Sodium-dependent proline transporter	3.49e-81	0.36
	TCF7L2	Transcription factor 7-like 2	4.549e-59	0.42
**8**	BRAF	Serine/threonine-protein kinase B-raf	1.588e-158	0.63
	ACKR3	Atypical chemokine receptor 3	6.265e-147	0.38
	BCR/ABL fusion	BCR/ABL p210 fusion protein	6.853e-82	0.63
	RAF1	RAF proto-oncogene serine/threonine-protein kinase	7.135e-80	0.63
	TCF7L2	Transcription factor 7-like 2	4e-78	0.37
**9**	BRAF	Serine/threonine-protein kinase B-raf	2.463e-152	0.53
	ACKR3	Atypical chemokine receptor 3	5.483e-142	0.40
	BCR/ABL fusion	BCR/ABL p210 fusion protein	1.381e-90	0.53
	TCF7L2	Transcription factor 7-like 2	1.671e-89	0.39
	CTNNB1	Catenin beta-1	1.201e-82	0.39
**10**	BCR/ABL fusion	BCR/ABL p210 fusion protein	1.97e-102	0.49
	ACKR3	Atypical chemokine receptor 3	5.763e-97	0.40
	CPT1B	Carnitine O-palmitoyltransferase 1, muscle isoform	6.867e-81	0.38
	BRAF	Serine/threonine-protein kinase B-raf	8.972e-74	0.49
	CPT2	Carnitine O-palmitoyltransferase 2, mitochondrial	6.733e-55	0.38
**imatinib**	BRAF	Serine/threonine-protein kinase B-raf	1.19e-141	1.00
	BCR/ABL fusion	BCR/ABL p210 fusion protein	8.174e-117	1.00
	SYK	Tyrosine-protein kinase SYK	1.176e-88	1.00
	ABL1	Tyrosine-protein kinase ABL1	6.475e-74	1.00
	KIT	Mast/stem cell growth factor receptor Kit	9.151e-68	1.00

This values were obtained from the from http://sea.bkslab.org/website (Reference-40)

**Table S4:** Cristal structures of ABL1

PDB ID: Chain ID	Resolution	Positions	Ligand	Details
2HYY: A [a]	2.40	228–500	Imatinib	Inactive (DFG-out)
2E2B: A [b]	2.20	229–515	Bafetinib	Inactive (DFG-out)
2HZ0: A [c]	2.10	228–497	NVP-AEG082	Inactive (DFG-out)
3CS9: A [d]	2.21	229–500	Nilotinib	Inactive (DFG-out)
3UE4: A [e]	2.42	229–512	Bosutinib	Inactive (DFG-out)
4YC8: A [f]	2.90	229–512	Dasatinib Analog	Inactive (Src-like)
2HZI: A [g]	1.70	229–500	PD180970	Intermediate (DFG-flip)
2HZ4: B [h]	2.80	228–500	NVP-AFN941	Active

[a] Human Abl kinase domain in complex with imatinib (STI571, Glivec),

[b] Crystal structure of the c-Abl kinase domain in complex with INNO-406,

[c] Abl kinase domain in complex with NVP-AEG082,

[d] Human ABL kinase in complex with nilotinib,

[e] Structural and spectroscopic analysis of the kinase inhibitor bosutinib binding to the Abl tyrosine kinase domain,

[f] C-Helix-Out Binding of Dasatinib Analog to c-Abl Kinase,

[g] Abl kinase domain in complex with PD180970,

[h] Abl kinase domain unligated and in complex with tetrahydrostaurosporine.

**Table S5:** Redocking the original ligands to the crystal structures of wild type ABL1

PDB ID: Chain ID	Ligand	Number of Torsions	Δ *G*	Size of the cluster	RMSD to reference
2HYY: A [a]	Imatinib	7	−14.5	38	1.1
2E2B: A [b]	Bafetinib	8	−14.9	32	1.3
2HZ0: A [c]	NVP-AEG082	8	−12.9	95	0.5
3CS9: A [d]	Nilotinib	7	−13.0	34	0.6
3UE4: A [e]	Bosutinib	9	−7.9	9	1.9
4YC8: A [f]	Dasatinib Analog	7	−11.0	32	1.2
2HZI: A [g]	PD180970	3	−11.2	100	0.6
2HZ4: B [h]	NVP-AFN941	4	−10.5	100	1.6

[a] Human Abl kinase domain in complex with imatinib (STI571, Glivec),

[b] Crystal structure of the c-Abl kinase domain in complex with INNO-406,

[c] Abl kinase domain in complex with NVP-AEG082,

[d] Human ABL kinase in complex with nilotinib,

[e] Structural and spectroscopic analysis of the kinase inhibitor bosutinib binding to the Abl tyrosine kinase domain,

[f] C-Helix-Out Binding of Dasatinib Analog to c-Abl Kinase,

[g] Abl kinase domain in complex with PD180970,

[h] Abl kinase domain unligated and in complex with tetrahydrostaurosporine.

**Table S6:** Cristal structures of BRAF.

PDB ID: Chain ID	Resolution	Positions	Ligand	Details
1UWH: A [a]	2.95	448–723	BAX	Inactive (DFG-out/αC-in)
4KSP: A [b]	2.93	445–723	TAK-632	Inactive (DFG-out/αC-in)
4JVG:A [c]	3.09	444–723	BIRB-796	Inactive (DFG-out/αC-in)
3C4C: A [d]	2.57	444–721	PLX-4720	Inactive (DFG-in/αC-out)
5CSW: A [e]	2.66	442–721	Dabrafenib	Inactive (DFG-in/αC-out)
2FB8: A [f]	2.90	445–723	SB-590885	Active (DFG-in/αC-in)
3D4Q: A [g]	2.57	433–726	SM5	Active (DFG-in/αC-in)

[a] The complex of wild type B-RAF and BAY439006,

[b] Crystal Structure of Human B-raf bound to a DFG-out Inhibitor TAK-632,

[c] B-Raf Kinase in Complex with Birb796,

[d] B-Raf Kinase in Complex with PLX4720,

[e] B-RAF in complex with Dabrafenib,

[f] Structure of the B-Raf kinase domain bound to SB-590885,

[g] Pyrazole-based inhibitors of B-Raf kinase.

**Table S7:** Redocking of the original ligands to the crystal structures of BRAF.

PDB ID: Chain ID	Ligand	Number of Torsions	Δ *G*	Size of cluster	RMSD to reference
1UWH: A [a]	BAX	6	−11.4	96	0.5
4KSP: A [b]	TAK-632	8	−13.3	20	1.3
4JVG:A [c]	BIRB-796	7	−14.4	62	0.7
3C4C: A [d]	PLX-4720	6	−9.7	23	1.3
5CSW: A [e]	Dabrafenib	6	−12.0	20	1.3
2FB8: A [f]	SB-590885	8	−8.9	62	1.9
3D4Q: A [g]	SM5	8	−8.7	19	1.9

[a] The complex of wild type B-RAF and BAY439006,

[b] Crystal Structure of Human B-raf bound to a DFG-out Inhibitor TAK-632,

[c] B-Raf Kinase in Complex with Birb796,

[d] B-Raf Kinase in Complex with PLX4720,

[e] B-RAF in complex with Dabrafenib,

[f] Structure of the B-Raf kinase domain bound to SB-590885,

[g] Pyrazole-based inhibitors of B-Raf kinase.

Figure S45 Comparison of ABL-imatinib (background) and ABL-Compound 5 (foreground) interactions. Imatinib and its contact residues are depicted in gray, while the common contact residues are marked with red circles.

Figure S46 Comparison of ABL-imatinib (background) and ABL-Compound 6 (foreground) interactions. Imatinib and its contact residues are depicted in gray, while the common contact residues are marked with red circles.

Figure S47 Comparison of ABL-imatinib (background) and ABL-Compound 7 (foreground) interactions. Imatinib and its contact residues are depicted in gray, while the common contact residues are marked with red circles.

Figure S48 Comparison of ABL-imatinib (background) and ABL-Compound 8 (foreground) interactions. Imatinib and its contact residues are depicted in gray, while the common contact residues are marked with red circles.

Figure S49 Comparison of ABL-imatinib (background) and ABL-Compound 9 (foreground) interactions. Imatinib and its contact residues are depicted in gray, while the common contact residues are marked with red circles.

## Figures and Tables

**Scheme:**
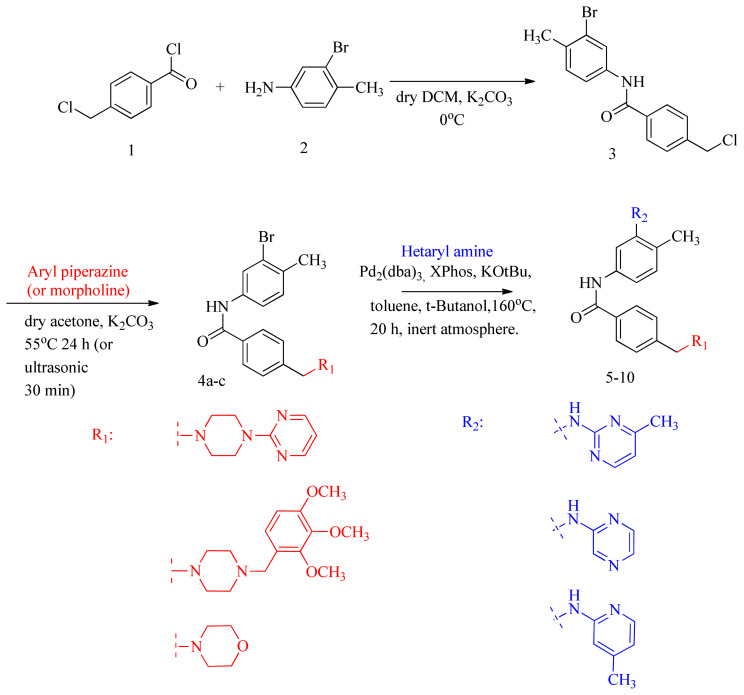
Synthesis route of novel imatinib derivatives (compound 5–10).

**Table 1:** Values of synthesized compounds found according to Lipinski rules.

Compound	Molecular weigth (g/mole)	MlogP(a)	HBA(b)	HBD(c)	T_PSA (A°)(d)	RB(e)
4a	466.388	3.726	6	1	61.36	5
4b	568.52	2.939	7	1	63.27	9
4c	389.22	3.166	4	1	41.57	4
5	494.603	3.191	9	2	99.17	7
6	596.734	2.377	10	2	101.08	10
7	480.576	2.18	9	2	99.17	7
8	417.514	2.862	7	2	79.38	6
9	416.526	3.017	6	2	66.49	6
10	595.746	2.477	9	2	88.19	11
imatinib	493.615	2.907	8	2	86.28	7

(a) Moruguchi octanol: water partition coefficient,

(b) hydrogen bond acceptor,

(c) hydrogen bond donor,

(d) topological polar surface area,

(e) rotable bonds.

**Table 2:**
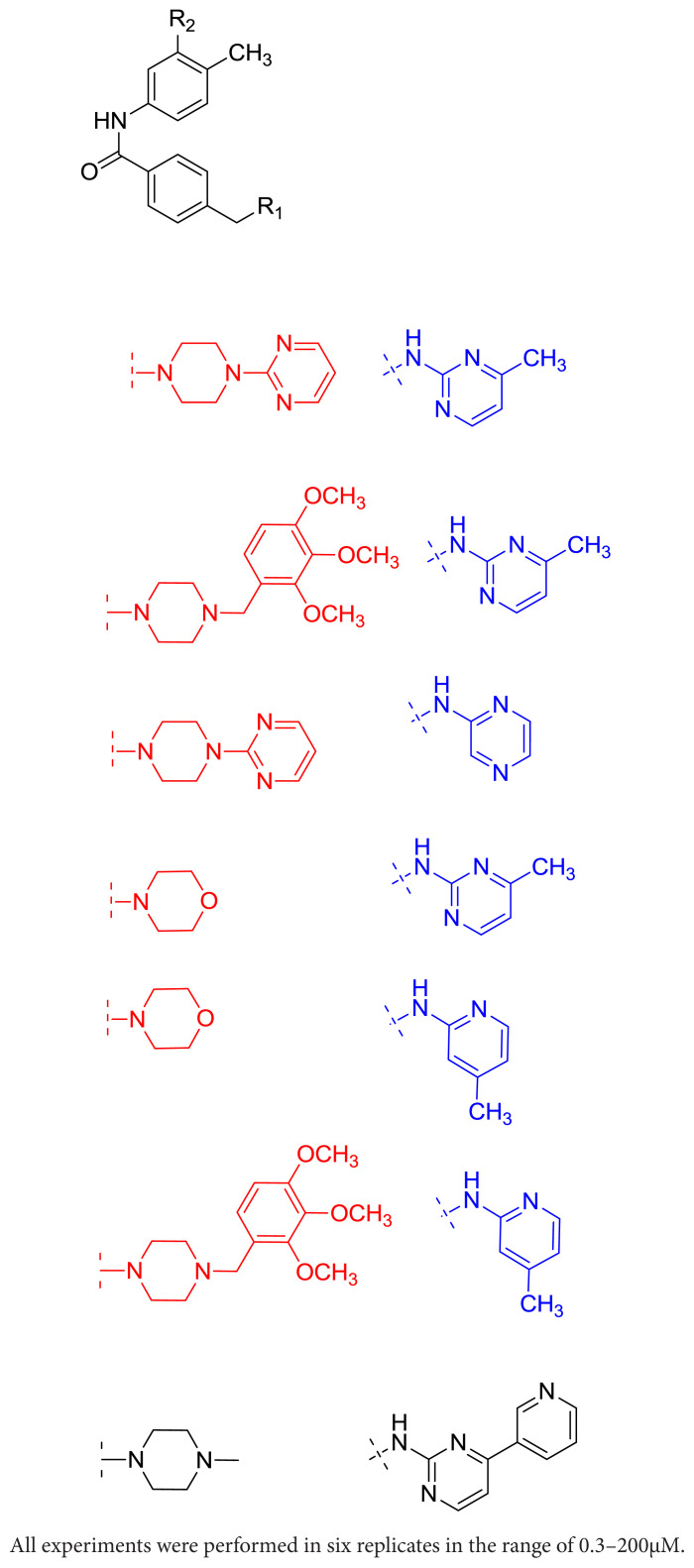
The chemical structures and IC_50_ values (μM) of the compounds 5–10 against K562, Nalm6, Molt4, Reh, Jurkat, Hek293T.

**Table 3:** The results of molecular docking to wild type human ABL1 kinase domain. Binding free energies (ΔG) correspond to the highest-ranking conformation of the largest cluster. The energy values are in kcal/mol. Number in parenthesis shows the percentage of independent runs that resulted in the same docked conformation. Values depicted in gray correspond to the docking simulations that did not meet our convergence criterion.

Structures	ΔG for compounds (kcal/mol)						
	5	6	7	8	9	10	Imatinib
2HYY[a]	−13.0 (35%)	−10.1 (15%)	−13.0 (92%)	−12.0 (62%)	−12.1 (98%)	−14.2 (42%)	−14.7 (34%)
2E2B[b]	−13.0 (49%)	−10.5 (11%)	−12.8 (90%)	−11.9 (81%)	12.1 (96%)	−13.9 (45%)	−14.5 (57%)
2HZ0[c]	−13.6 (43%)	−9.5 (6%)	−13.3 (75%)	−12.4 (64%)	−12.6 (67%)	−11.6 (9%)	−14.6 (78%)
3CS9[d]	−9.4 (33%)	−9.6 (12%)	−12.9 (49%)	−10.9 (59%)	−11.3 (79%)	−12.8 (30%)	−13.1 (38%)
3UE4[e]	−9.8 (20%)	−10.5 (8%)	−8.2 (13%)	−9.1 (42%)	−8.9 (47%)	−11.2 (21%)	−10.1 (34%)
4YC8[f]	−10.1 (15%)	−10.2 (8%)	−9.7 (36%)	−9.0 (35%)	−8.9 (46%)	−9.9 (22%)	−9.6 (25%)
2HZI[g]	−11.3 (23%)	−11.8 (20%)	−11.2 (22%)	−11.2 (91%)	−10.1 (38%)	−11.1 (21%)	−12.1 (51%)
2HZ4[h]	−8.7 (15%)	−11. 0 (12%)	−9.2 (29%)	−9.3 (66%)	−8.8 (39%)	−9.7 (26%)	−9.9 (56%)

[a] Human Abl kinase domain in complex with imatinib,

[b] Crystal structure of the c-Abl kinase domain in complex with INNO-406,

[c] Abl kinase domain in complex with NVP-AEG082,

[d] Human ABL kinase in complex with nilotinib,

[e] Structural and spectroscopic analysis of the kinase inhibitor bosutinib binding to the Abl tyrosine kinase domain,

[f] C-Helix-Out Binding of Dasatinib Analog to c-Abl Kinase,

[g] Abl kinase domain in complex with PD180970,

[h] Abl kinase domain unligated and in complex with tetrahydrostaurosporine.

**Table 4:** The results of molecular docking to wild type human BRAF kinase domain. Binding free energies (ΔG) correspond to the highest-ranking conformation of the largest cluster. The energy values are in kcal/mol. Number in parenthesis shows the percentage of independent runs that resulted in the same docked conformation. Values depicted in gray correspond to the docking simulations that did not meet our convergence criterion.

ΔG for compounds (kcal/mol)						
Structures	5	6	7	8	9	10
1UHW[a]	−11.7 (39%)	−10.7 (11%)	−12.0 (32%)	−10.5 (56%)	−11.2 (76%)	−11.3 (10%)
4KSP[b]	−11.1 (10%)	−9.8 (16%)	−10.1 (52%)	−10.0 (45%)	10.7 (52%)	−11.5 (20%)
4JVG[c]	−10.8 (56%)	−8.7 (9%)	−11.0 (55%)	−10.2 (27%)	−9.8 (54%)	−10.3 (14%)
3C4C[d]	−8.9 (22%)	−10.4 (15%)	−10.2 (20%)	−7.6 (23%)	−9.5 (43%)	−9.5 (24%)
5C5W[e]	−8.7 (29%)	−10.8 (16%)	−11.3 (6%)	−8.5 (52%)	−9.6 (86%)	−11.5 (45%)
2FB8[f]	−9.7 (35%)	−8.9 (11%)	−9.0 (15%)	−8.7 (42%)	−8.8 (50%)	−9.5 (8%)
3D4Q[g]	−9.3 (24%)	−9.8 (16%)	−9.5 (13%)	−9.6 (37%)	−8.6 (30%)	−8.4 (13%)

[a] Solution structure of the DEP domain of mouse pleckstrin,

[b] Crystal Structure of Human B-raf bound to a DFG-out Inhibitor TAK-632,

[c] B-Raf Kinase in Complex with Birb796,

[d] B-Raf Kinase in Complex with PLX4720,

[e] 1.25 A resolution structure of an RNA 20-mer,

[f] Structure of the B-Raf kinase domain bound to SB-590885,

[g] Pyrazole-based inhibitors of B-Raf kinase.
