# Supplementary figures and images for: Synthesis, molecular modeling, and biological evaluation of novel imatinib derivatives as anticancer agents
Source: Turk J Chem. 2021 Sep 6;46(1):86–102. doi: 10.3906/kim-2107-23 (PMC10734772; doi:10.3906/kim-2107-23)

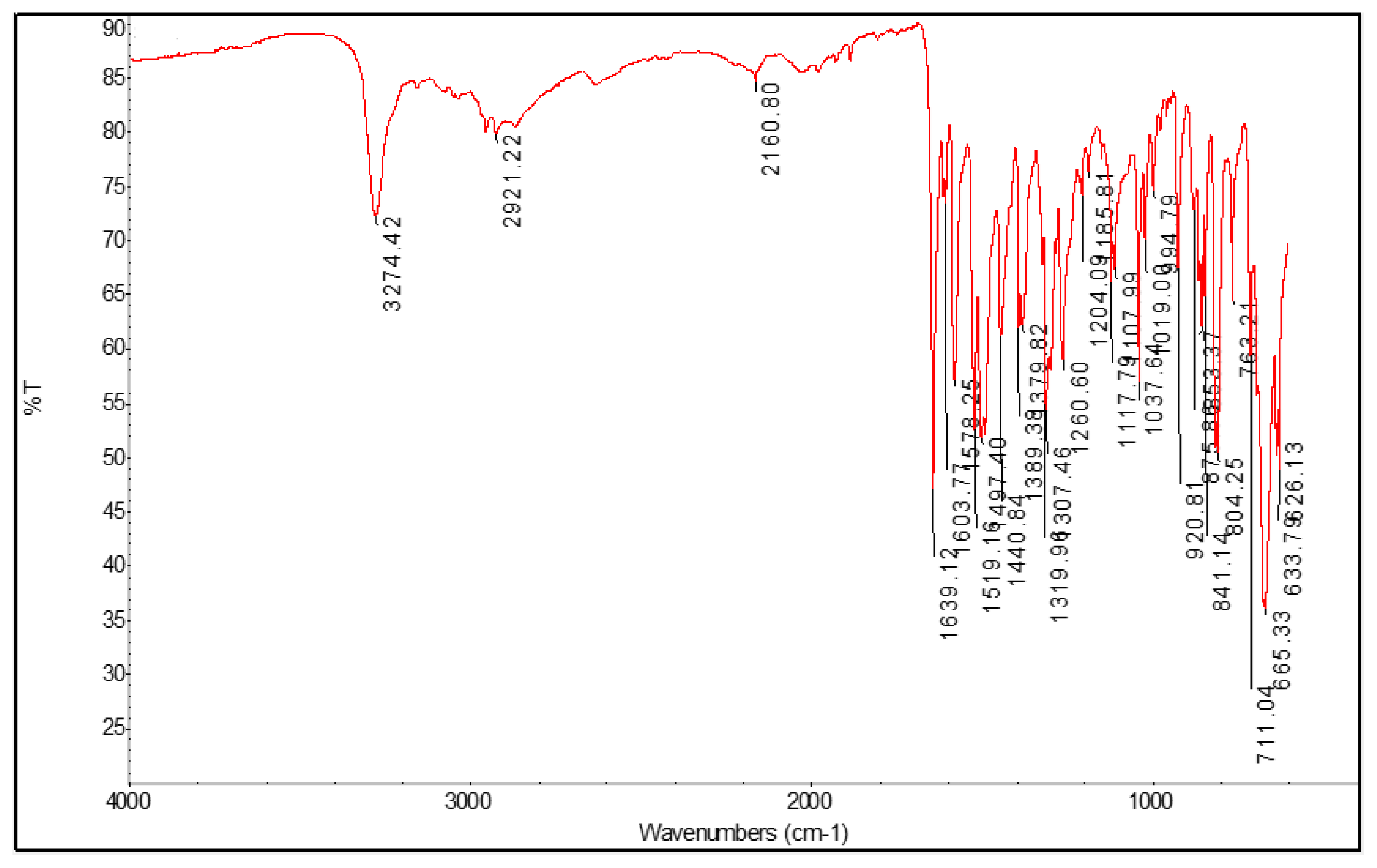

Supplement: Figure S1 — IR spectrum of Compound 3. [file turkjchem-46-1-86s1.tif]

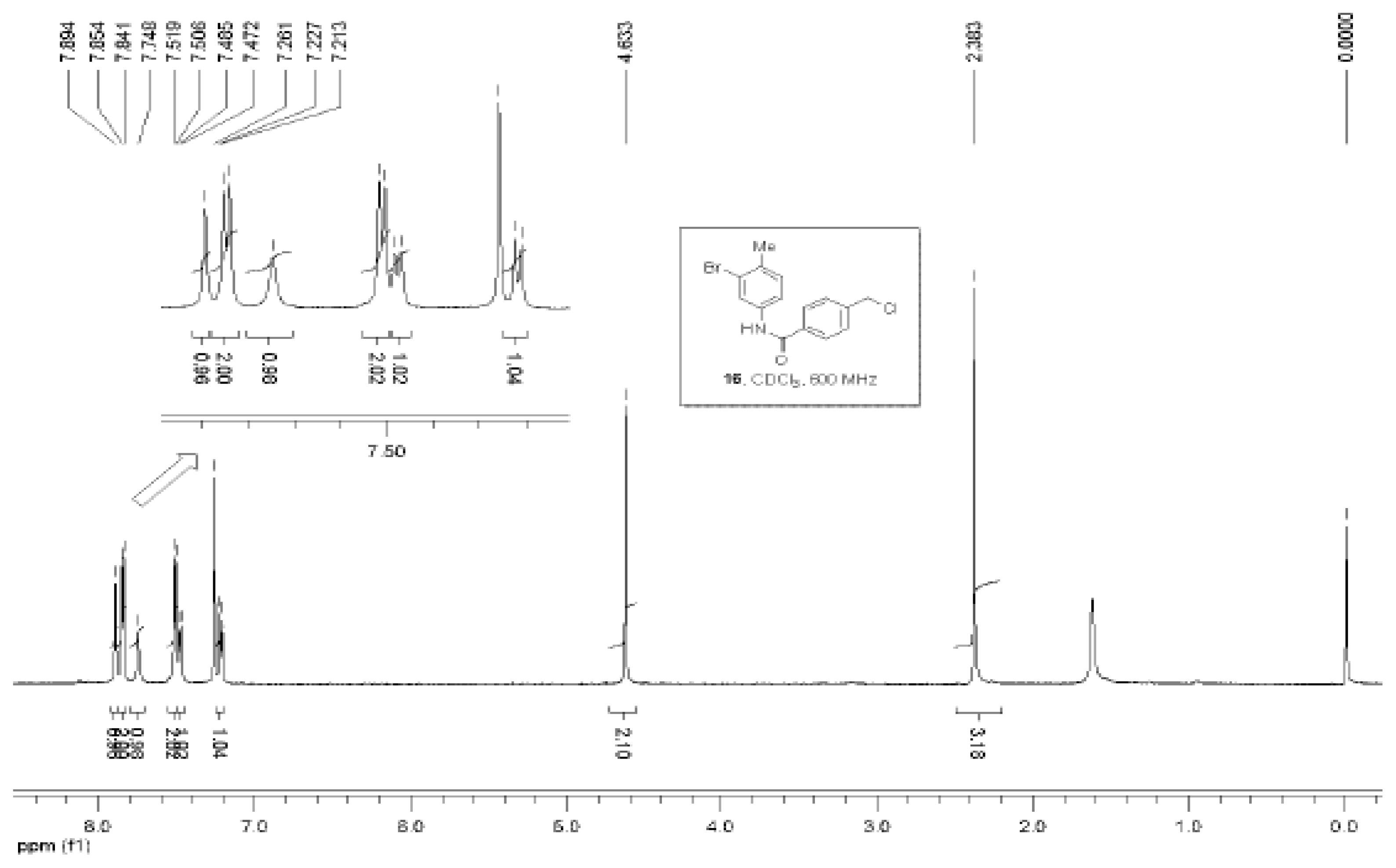

Supplement: Figure S2 — 1H-NMR spectrum of Compound 3 (Reference-13). [file turkjchem-46-1-86s2.tif]

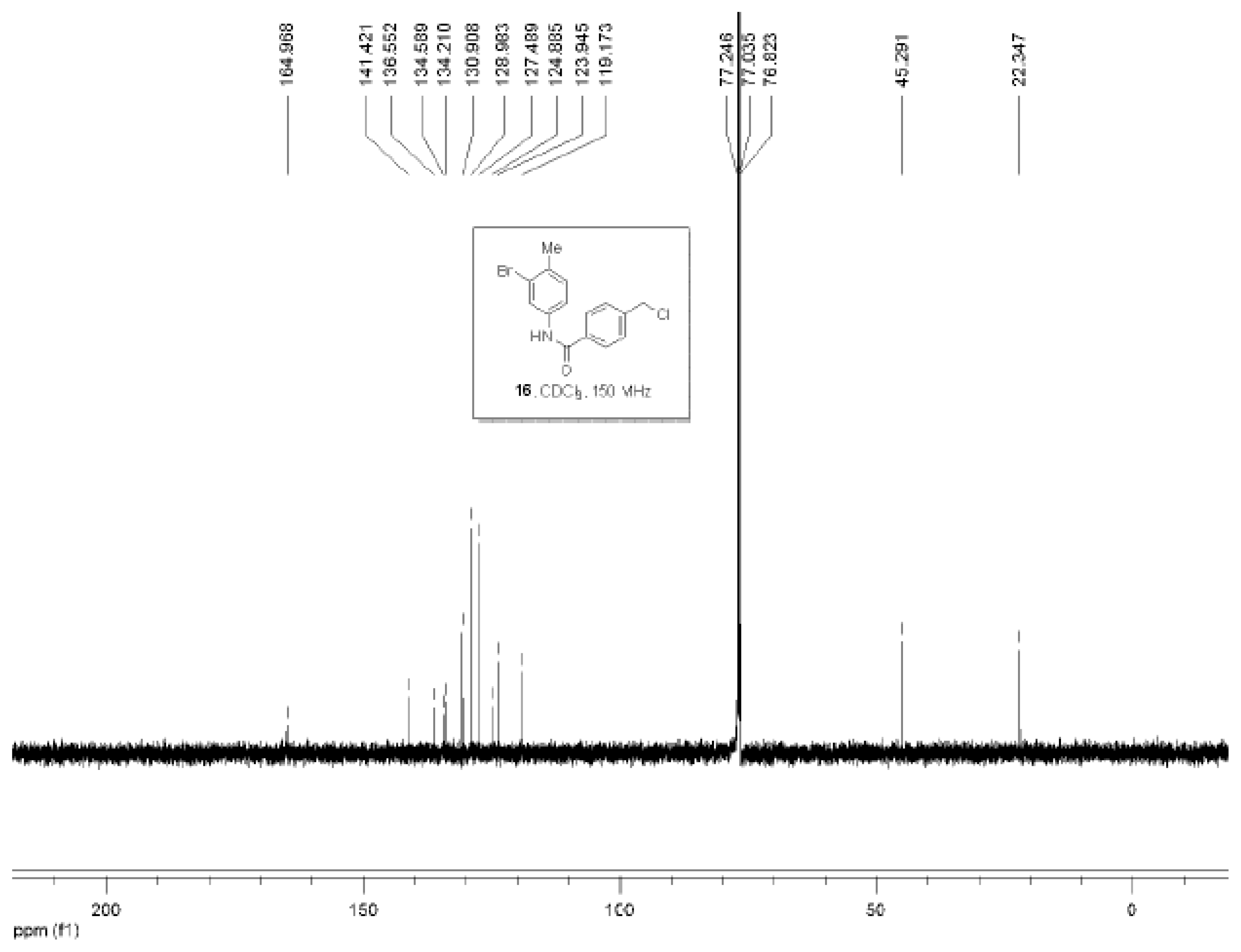

Supplement: Figure S3 — 13C-NMR spectrum of Compound 3 (Reference-13). [file turkjchem-46-1-86s3.tif]

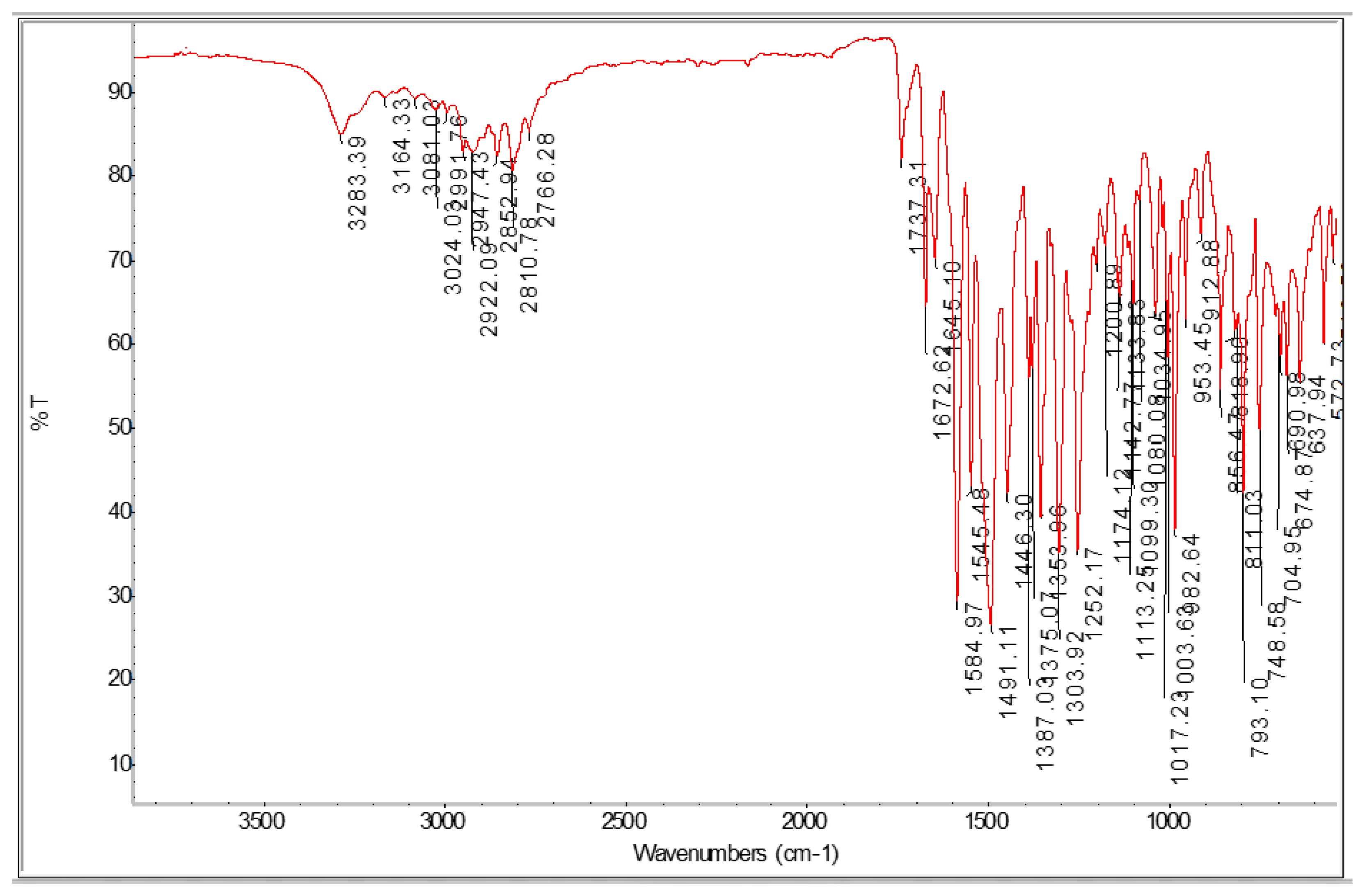

Supplement: Figure S4 — IR spectrum of Compound 4a. [file turkjchem-46-1-86s4.tif]

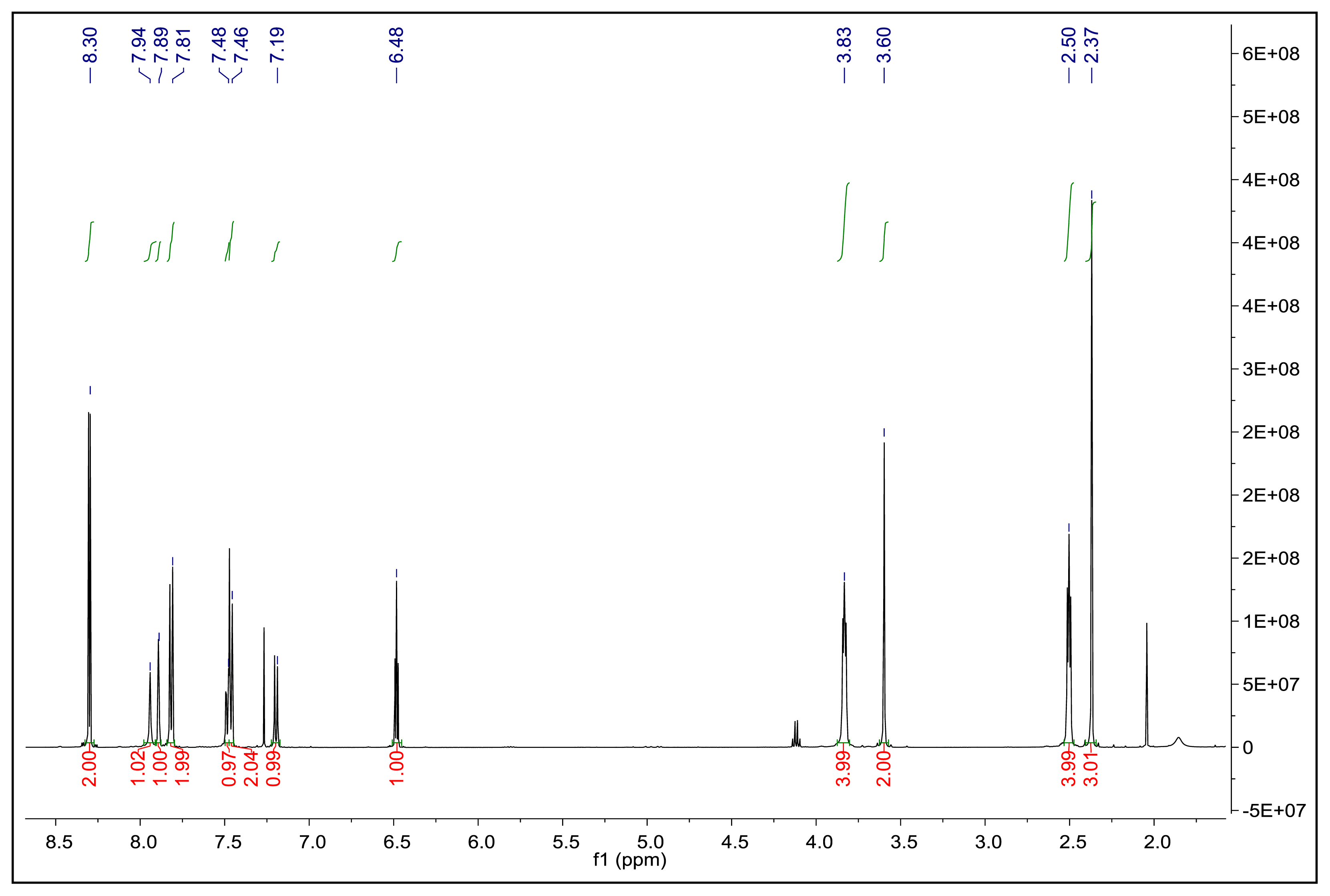

Supplement: Figure S5 — 1H-NMR spectrum of Compound 4a. [file turkjchem-46-1-86s5.tif]

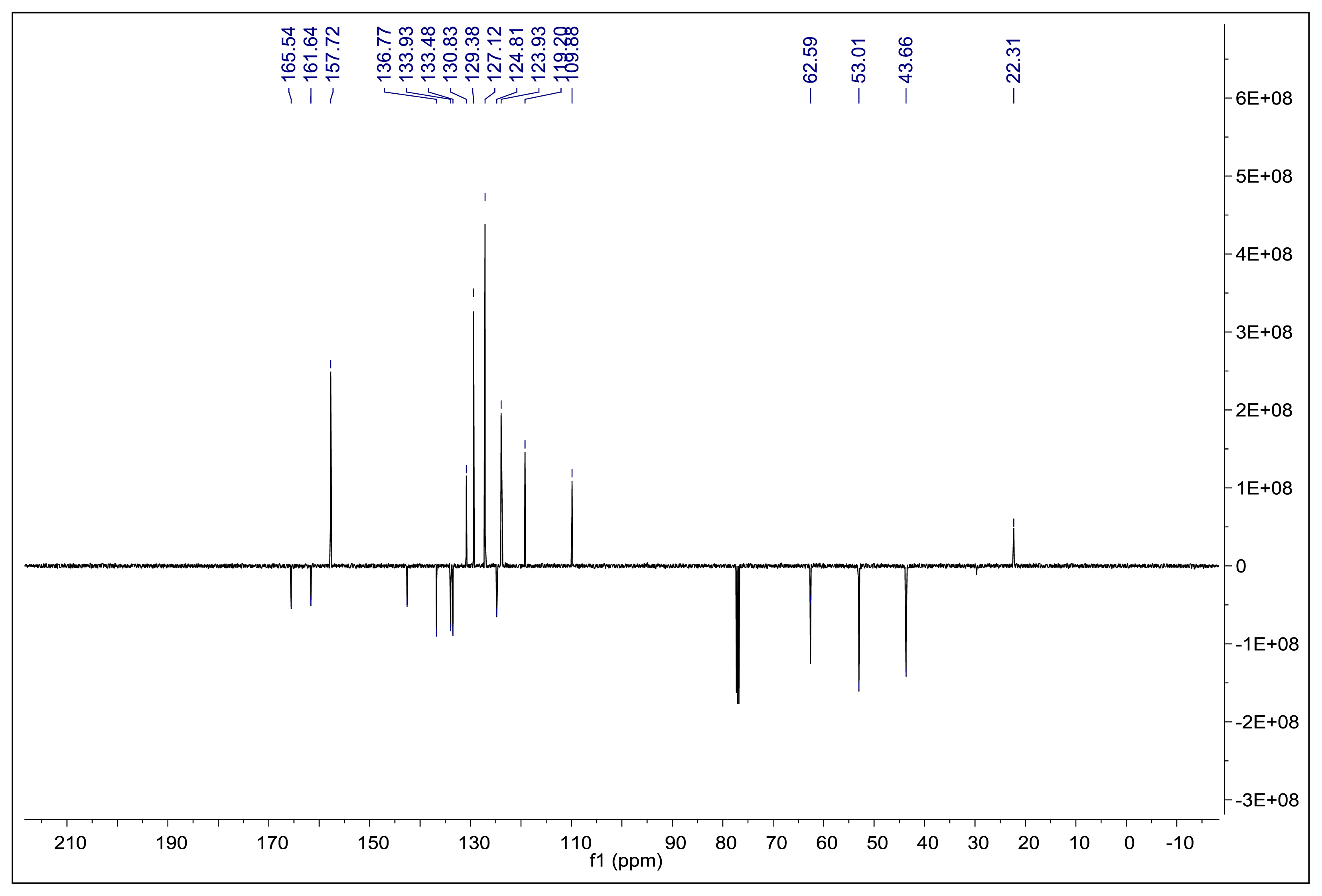

Supplement: Figure S6 — APT spectrum of Compound 4a. [file turkjchem-46-1-86s6.tif]

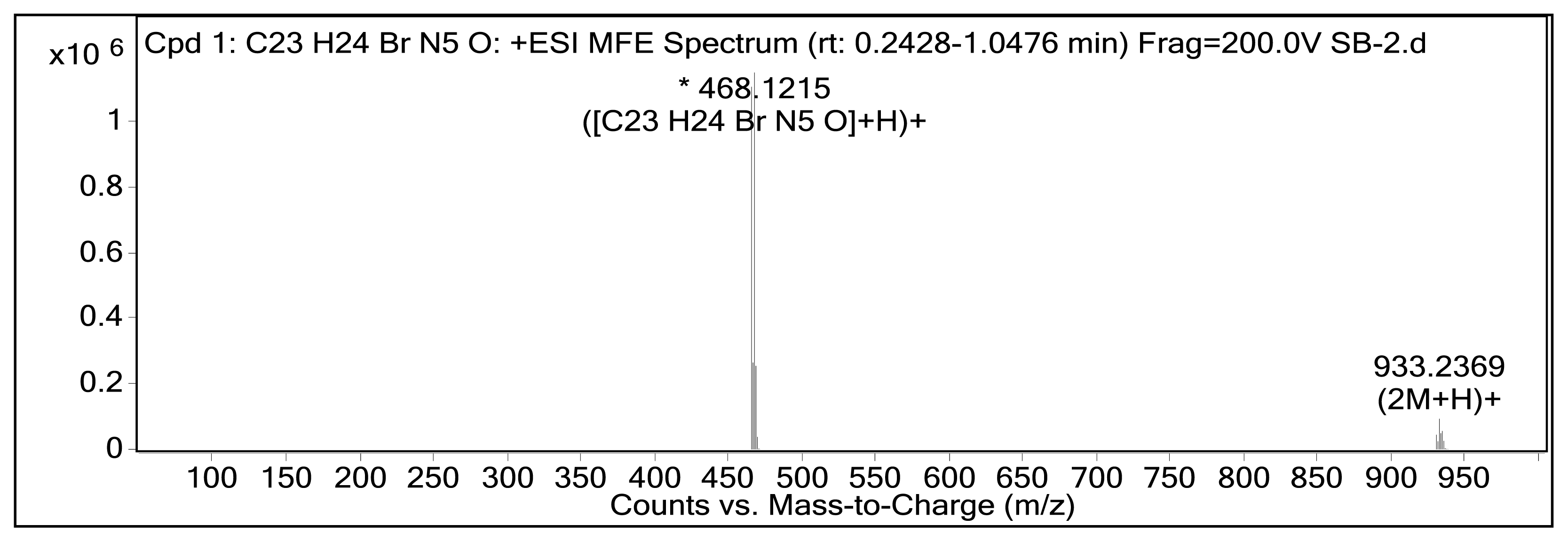

Supplement: Figure S7 — HRMS Spectrum of Compound 4a. [file turkjchem-46-1-86s7.tif]

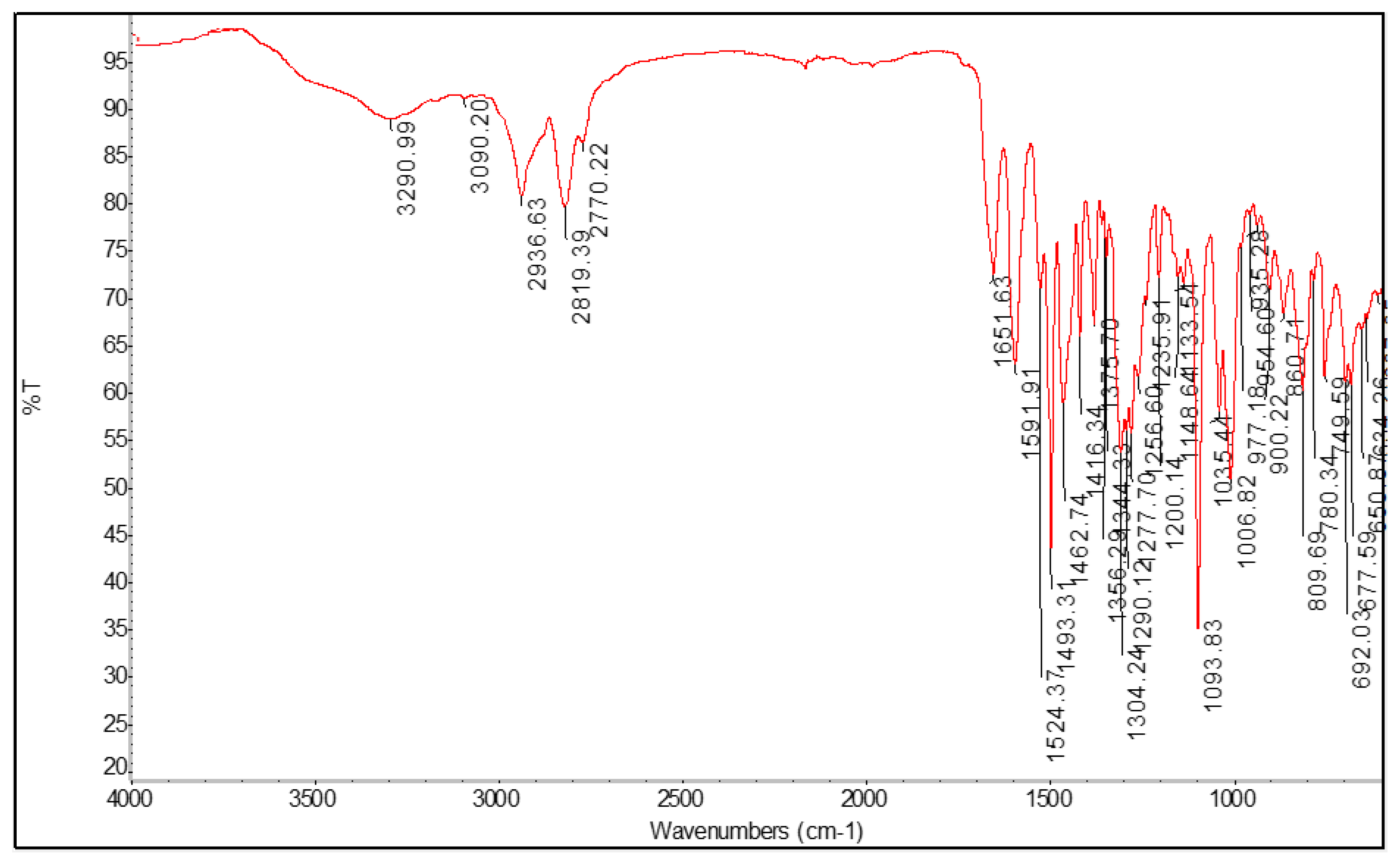

Supplement: Figure S8 — IR spectrum of Compound 4b. [file turkjchem-46-1-86s8.tif]

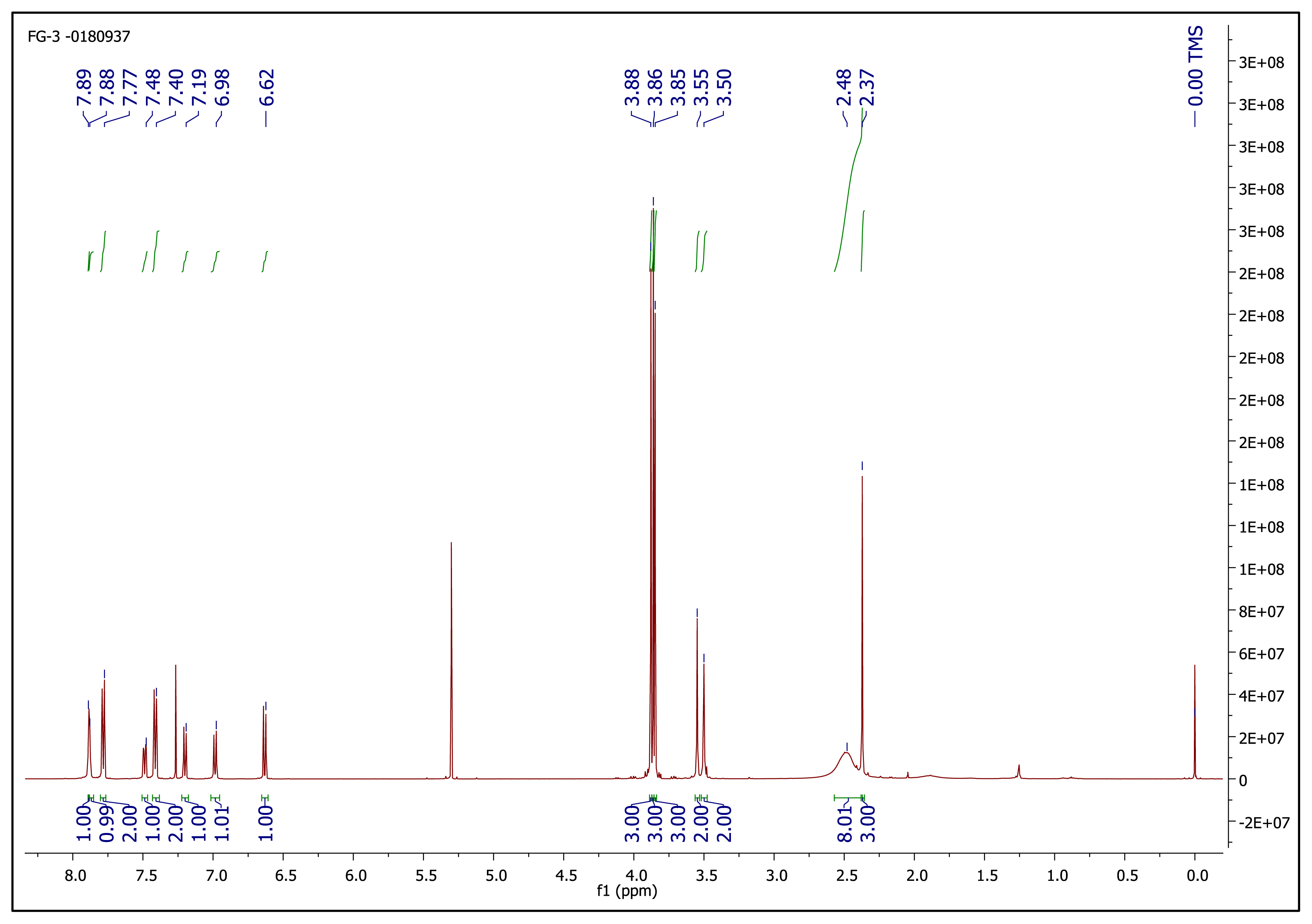

Supplement: Figure S9 — 1H-NMR spectrum of Compound 4b. [file turkjchem-46-1-86s9.tif]

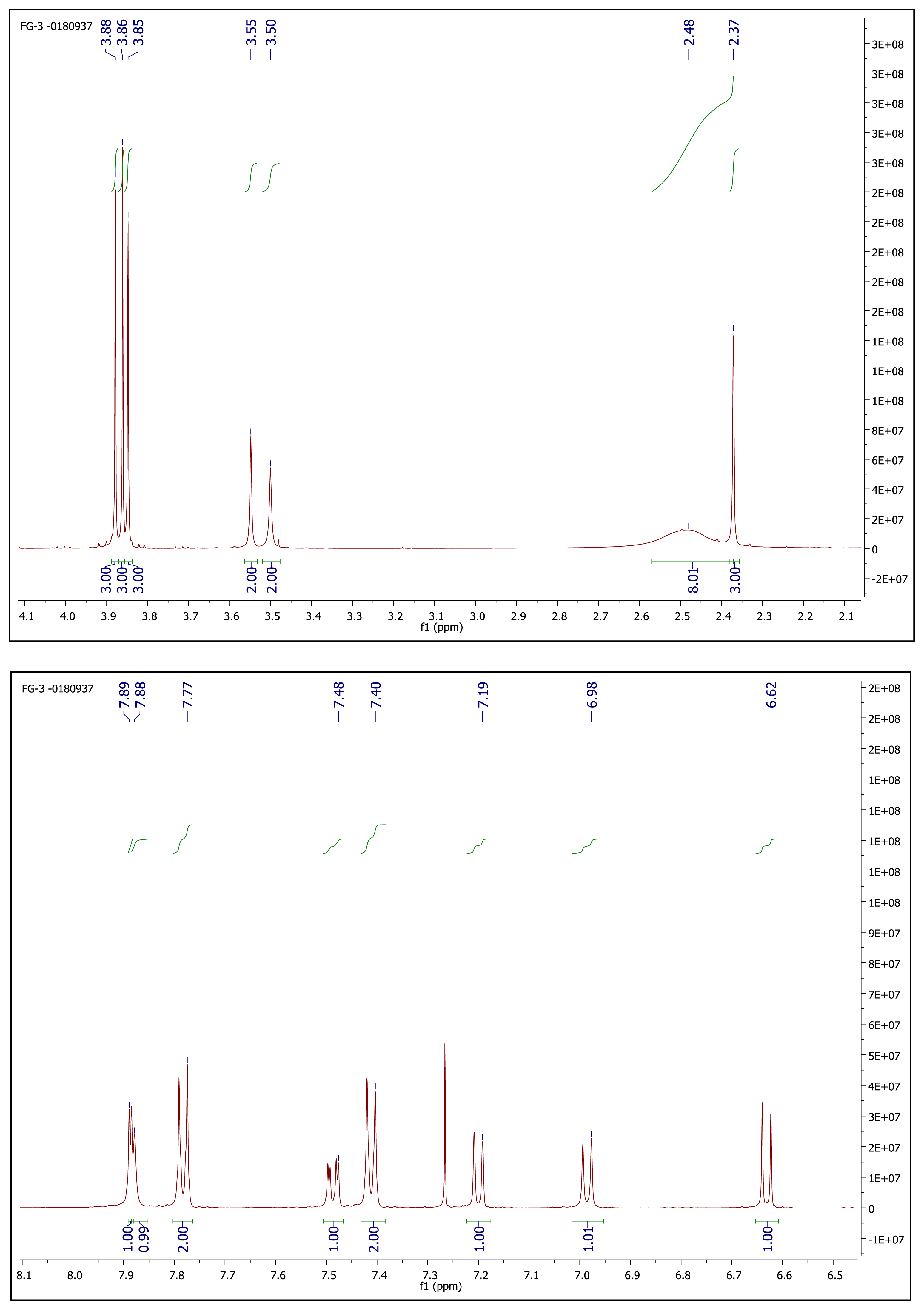

Supplement: Figure S10–S11 — 1H-NMR spectrum of Compound 4b. [file turkjchem-46-1-86s10.tif]

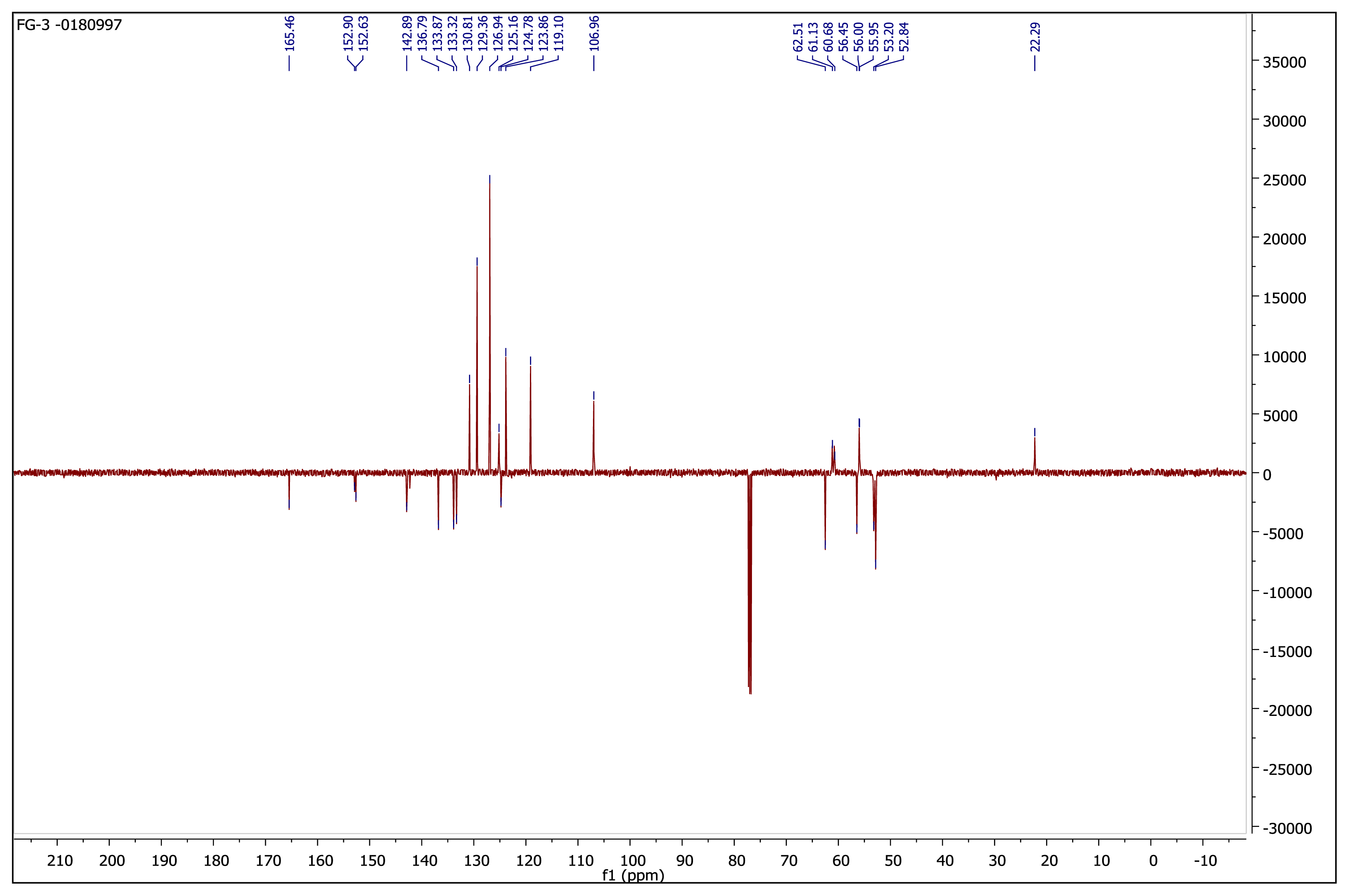

Supplement: Figure S12 — APT spectrum of Compound 4b. [file turkjchem-46-1-86s11.tif]

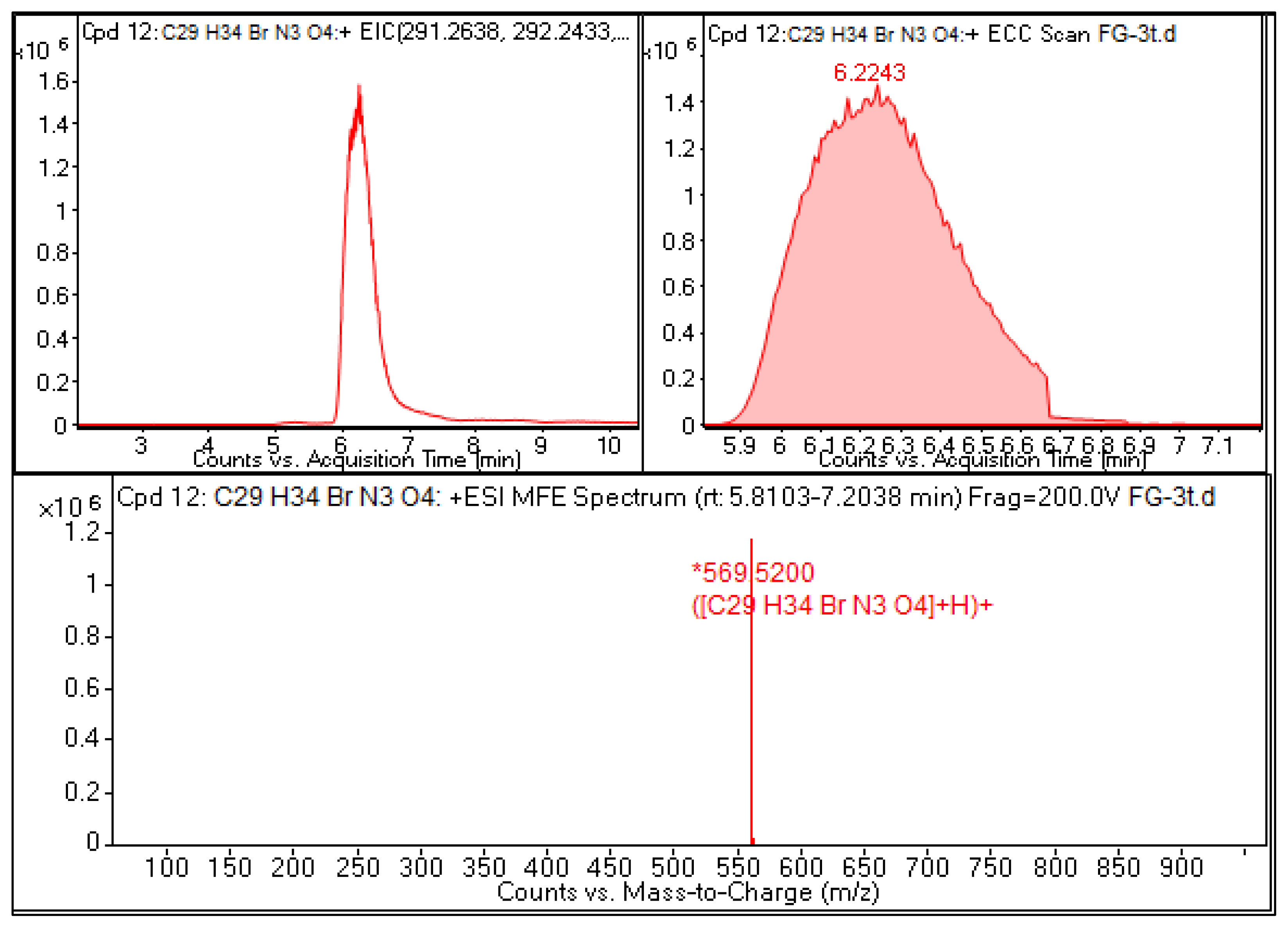

Supplement: Figure S13 — HRMS Spectrum of Compound 4b. [file turkjchem-46-1-86s12.tif]

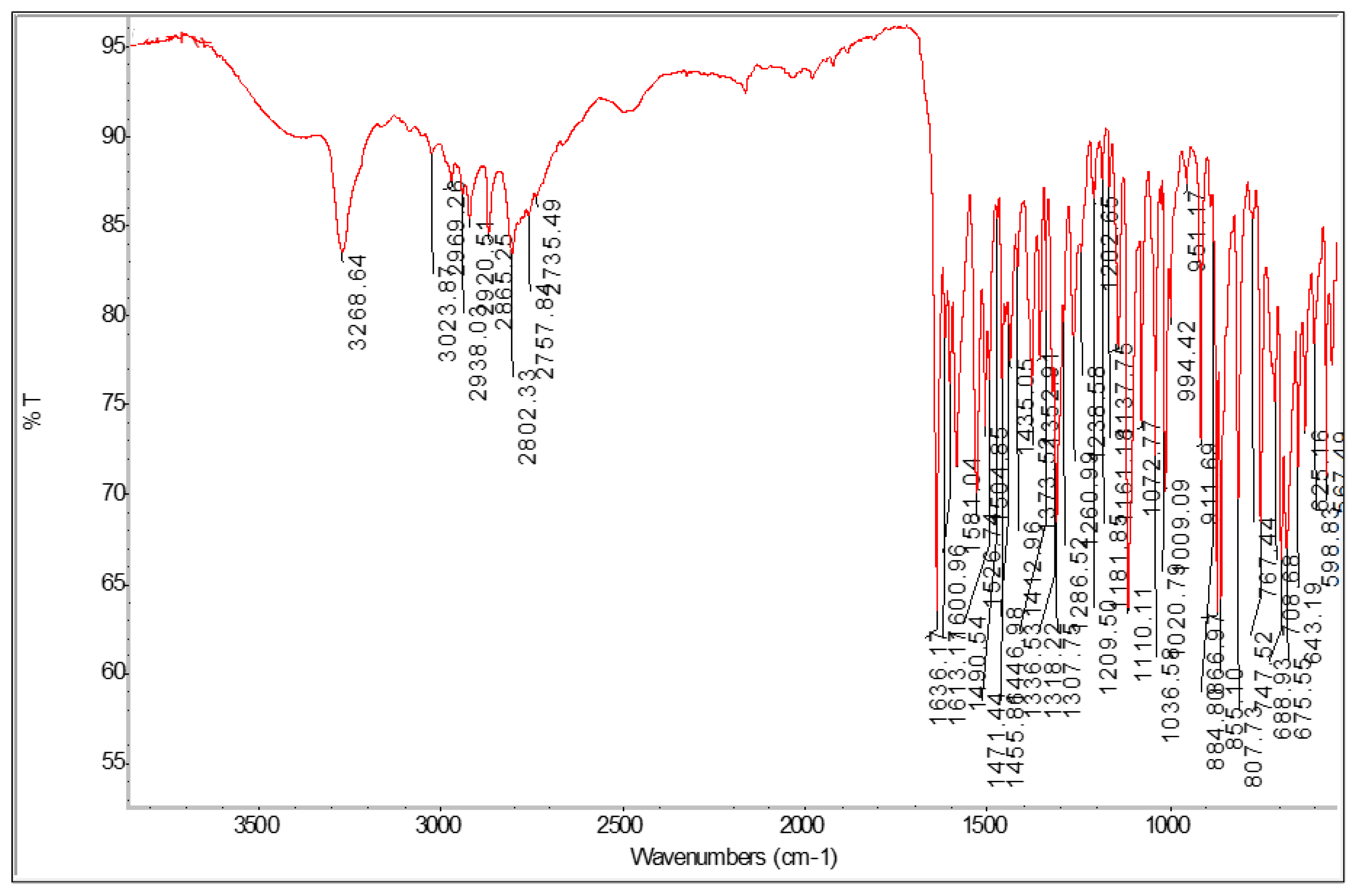

Supplement: Figure S14 — IR Spectrum of Compound 4c. [file turkjchem-46-1-86s13.tif]

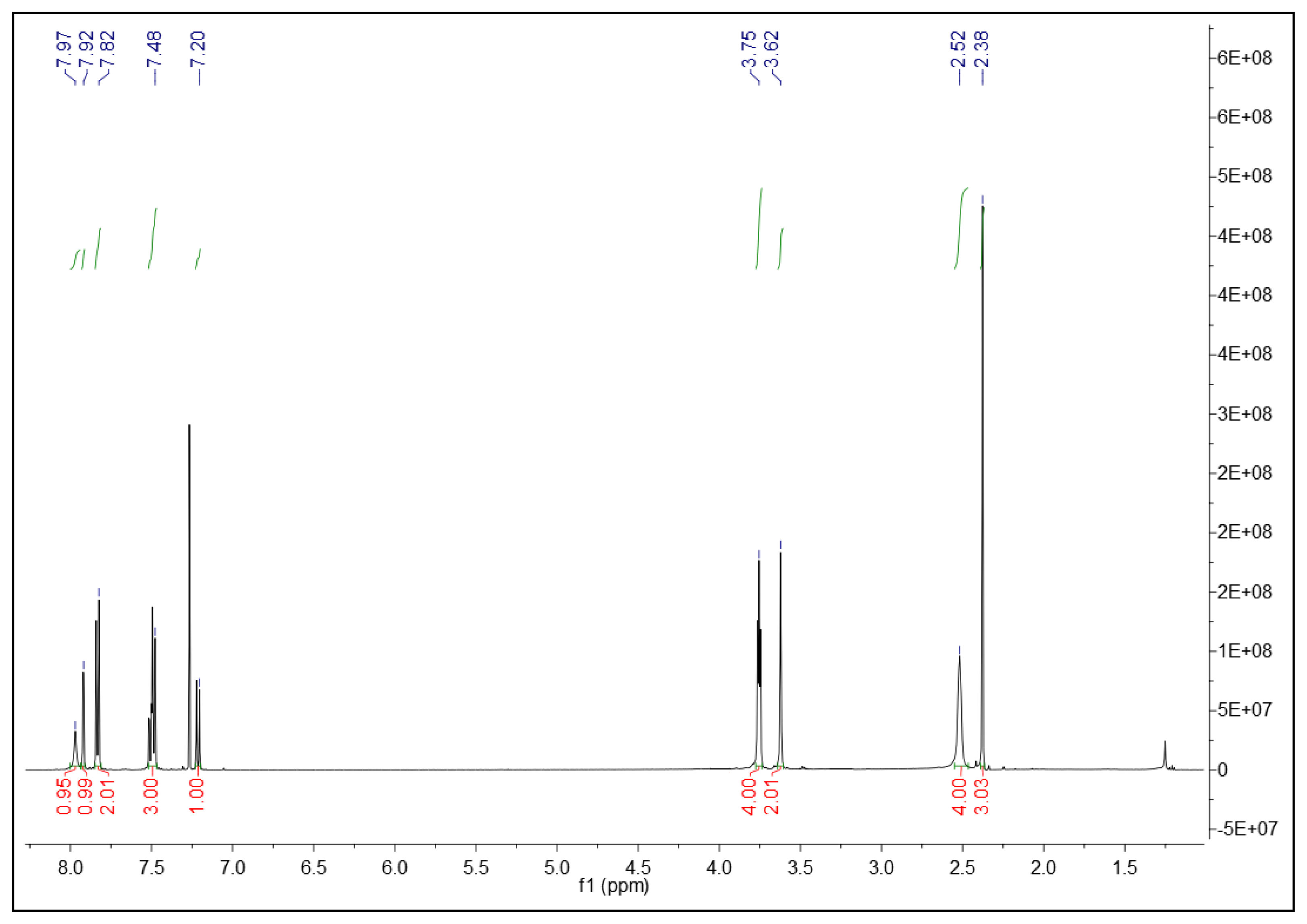

Supplement: Figure S15 — 1H-NMR spectrum of Compound 4c. [file turkjchem-46-1-86s14.tif]

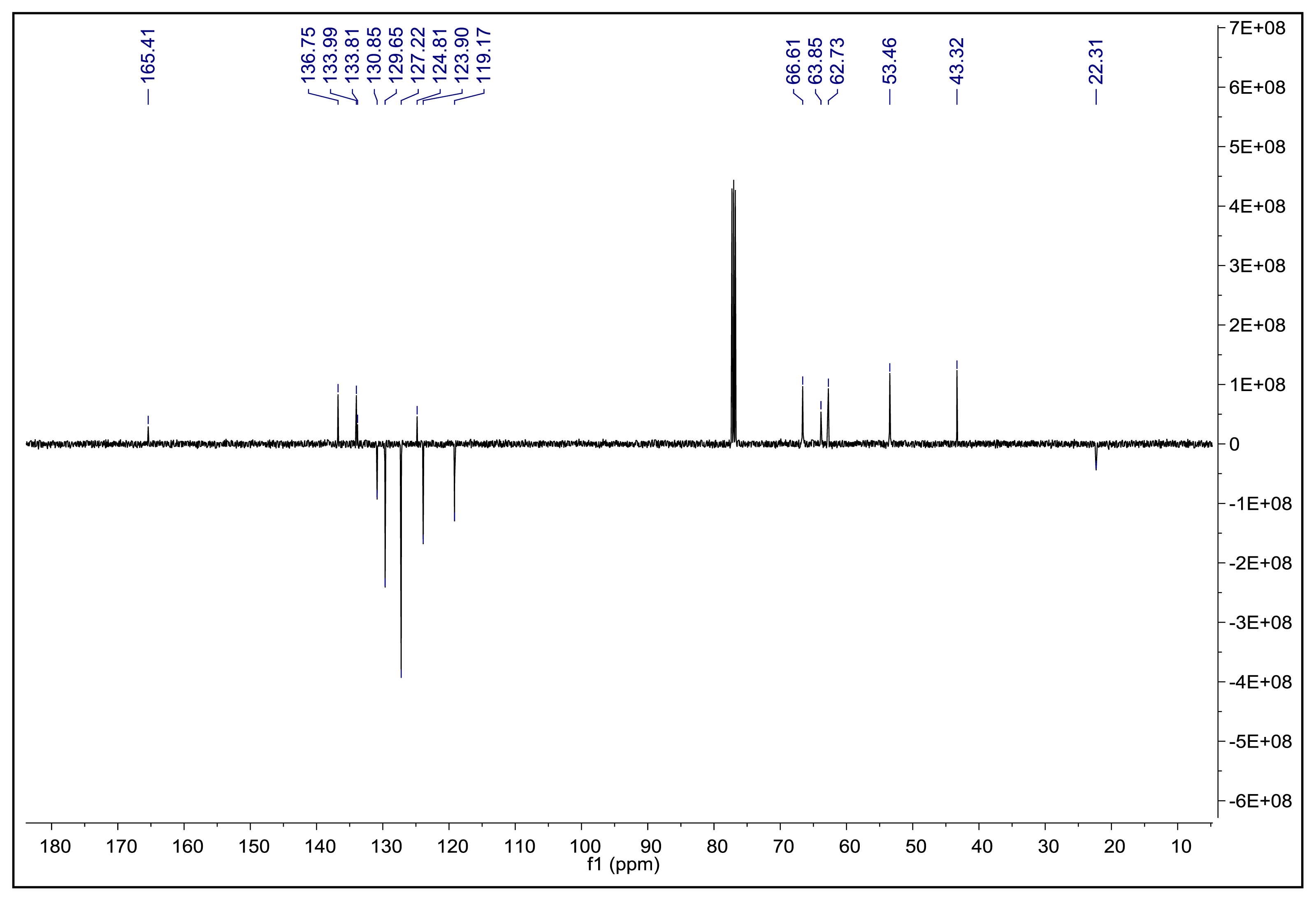

Supplement: Figure S16 — APT spectrum of Compound 4c. [file turkjchem-46-1-86s15.tif]

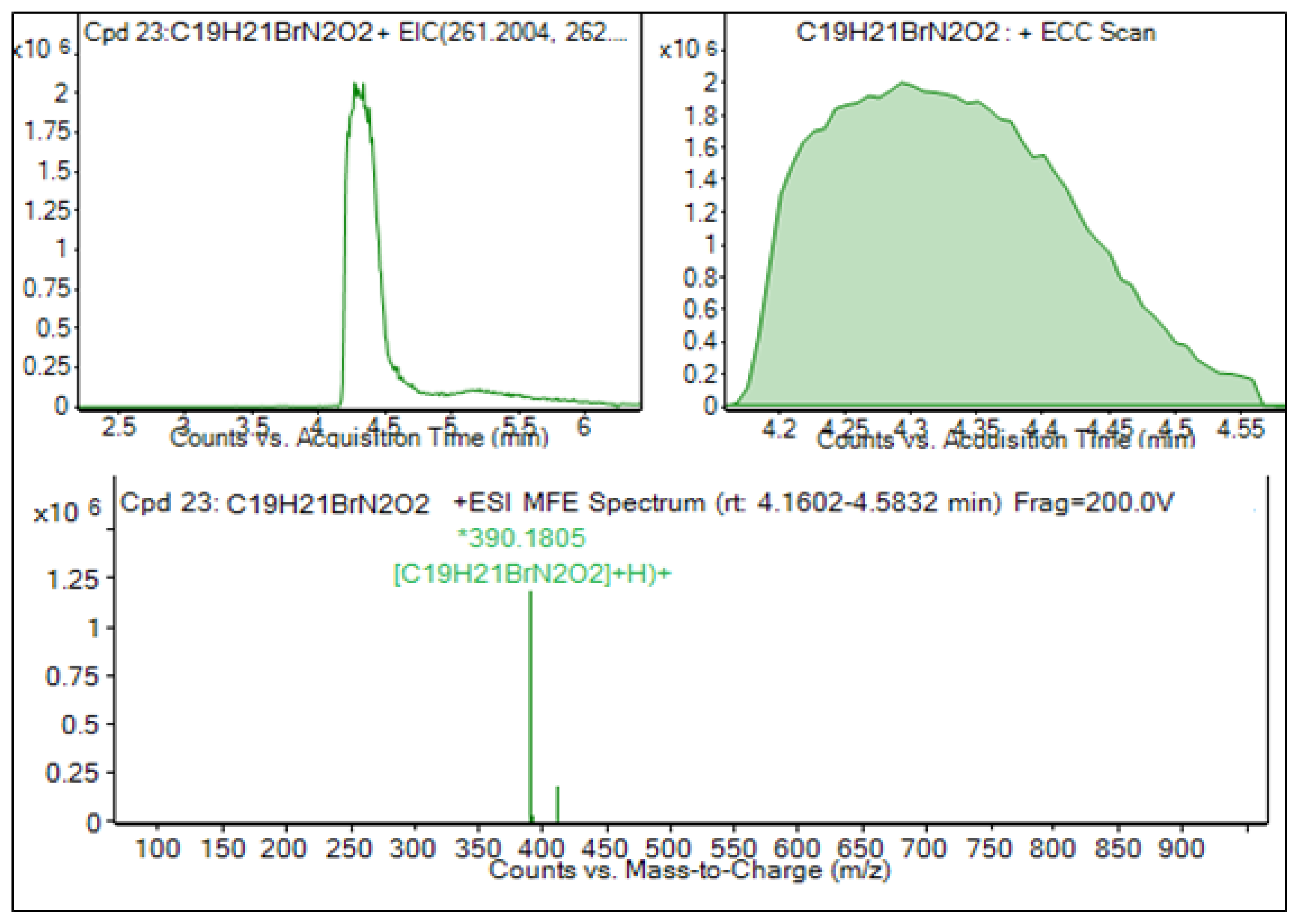

Supplement: Figure S17 — HRMS Spectrum of Compound 4c. [file turkjchem-46-1-86s16.tif]

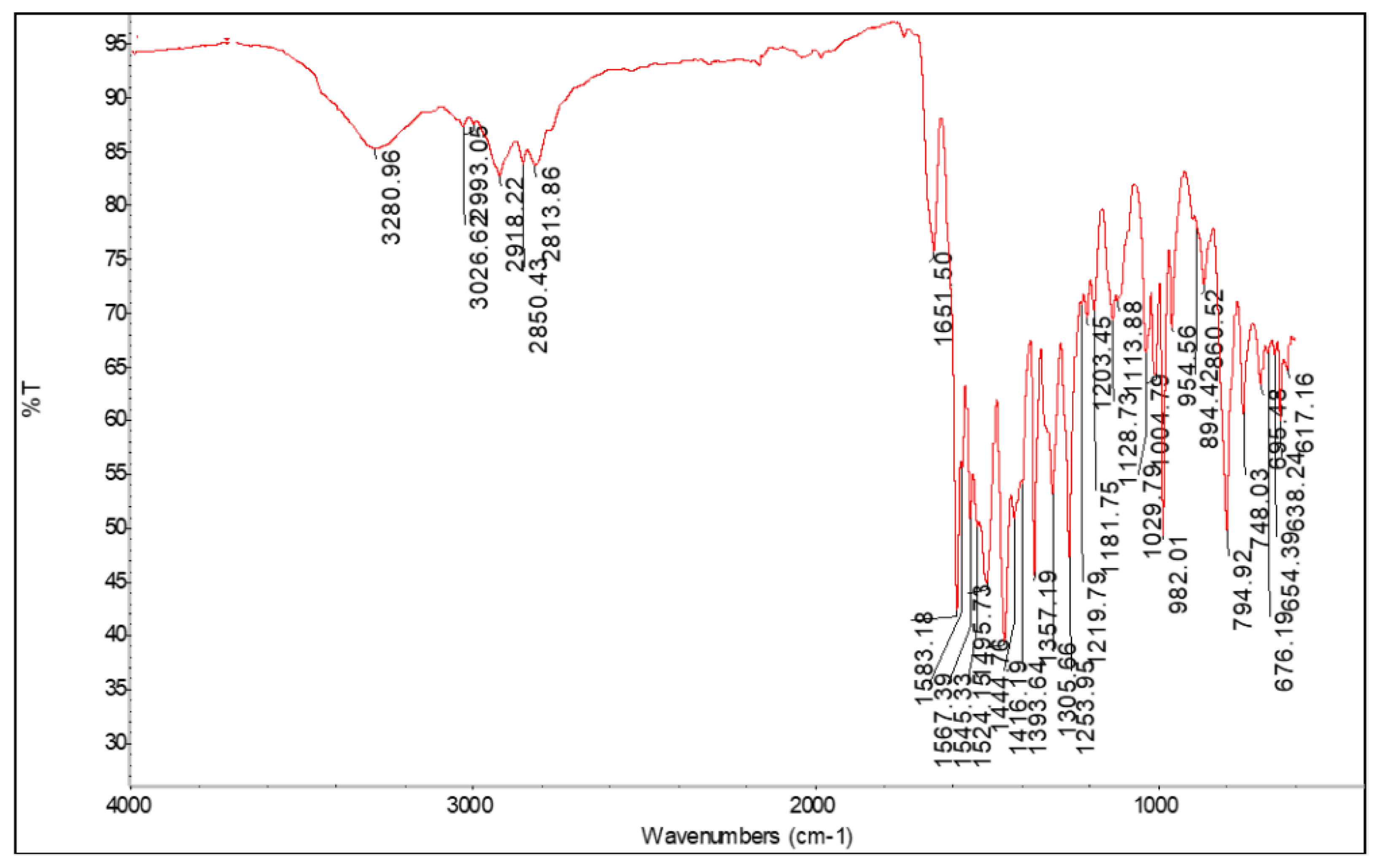

Supplement: Figure S18 — IR Spectrum of Compound 5. [file turkjchem-46-1-86s17.tif]

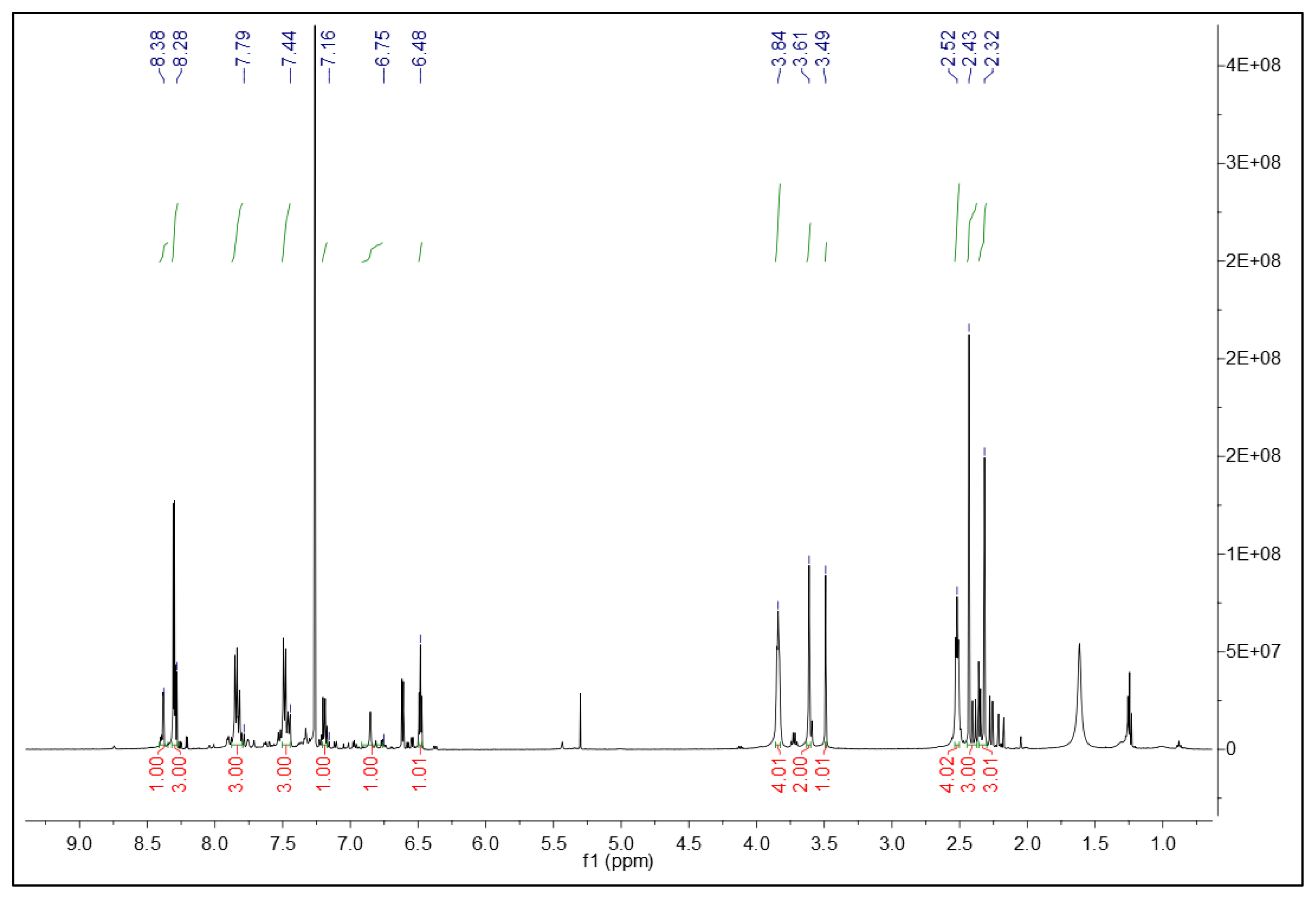

Supplement: Figure S19 — 1H-NMR spectrum of Compound 5. [file turkjchem-46-1-86s18.tif]

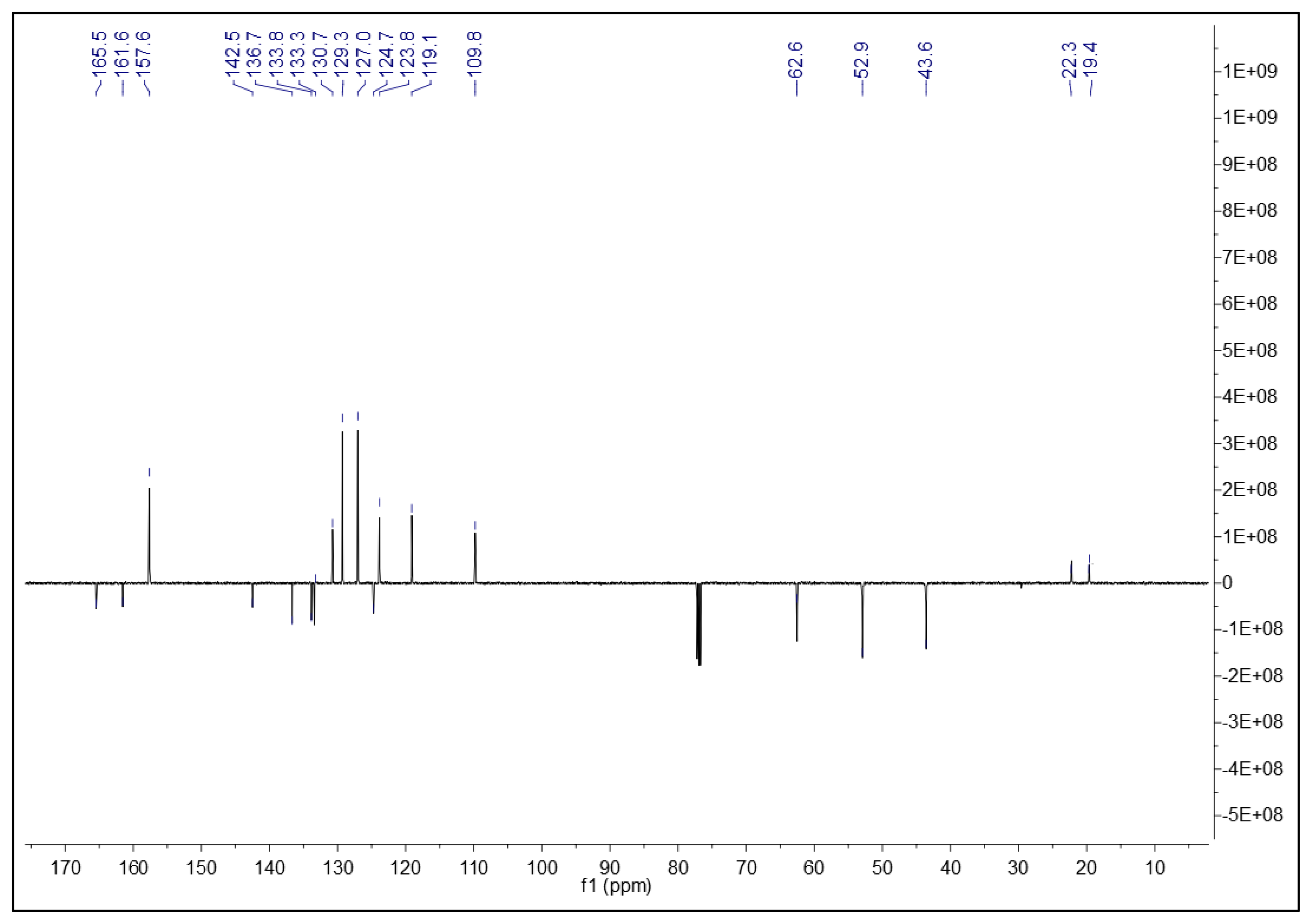

Supplement: Figure S20 — APT spectrum of Compound 5. [file turkjchem-46-1-86s19.tif]

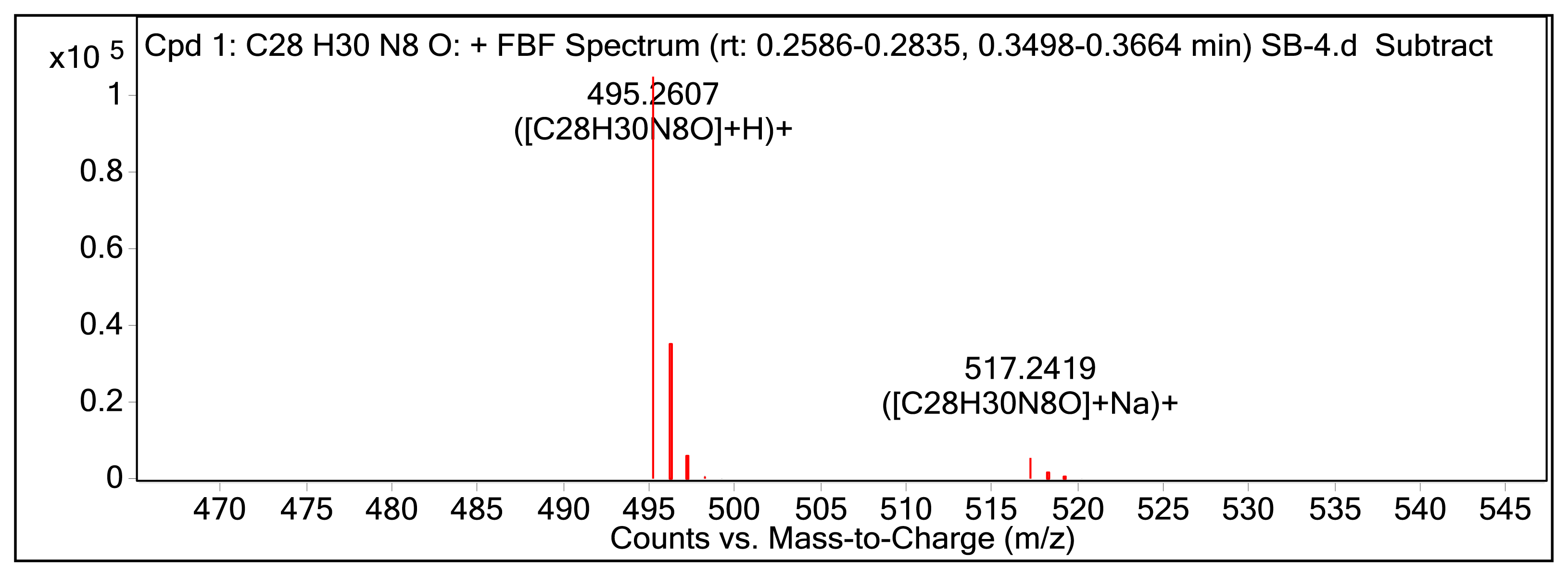

Supplement: Figure S21 — HRMS Spectrum of Compound 5. [file turkjchem-46-1-86s20.tif]

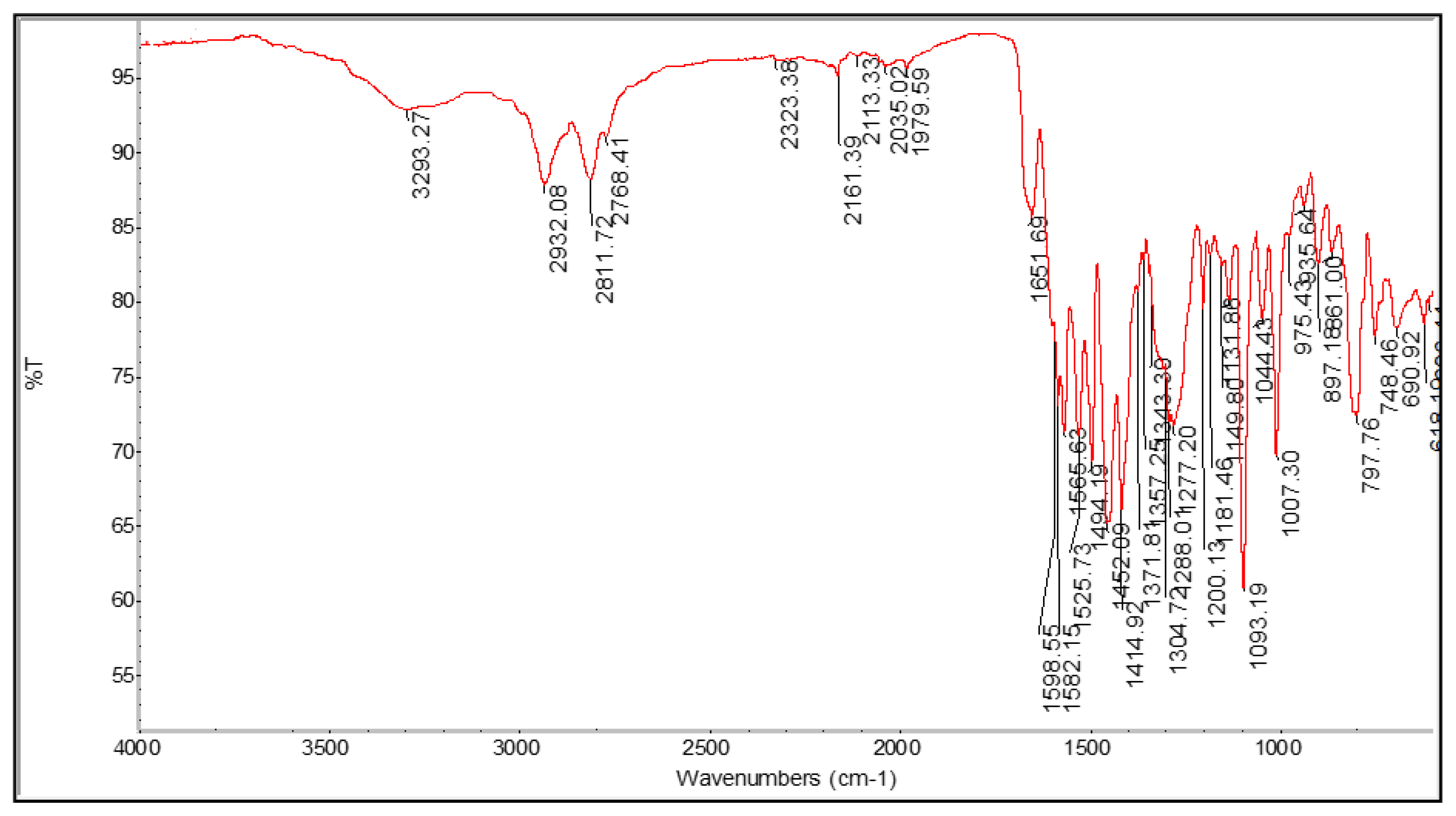

Supplement: Figure S22 — IR spectrum of Compound 6. [file turkjchem-46-1-86s21.tif]

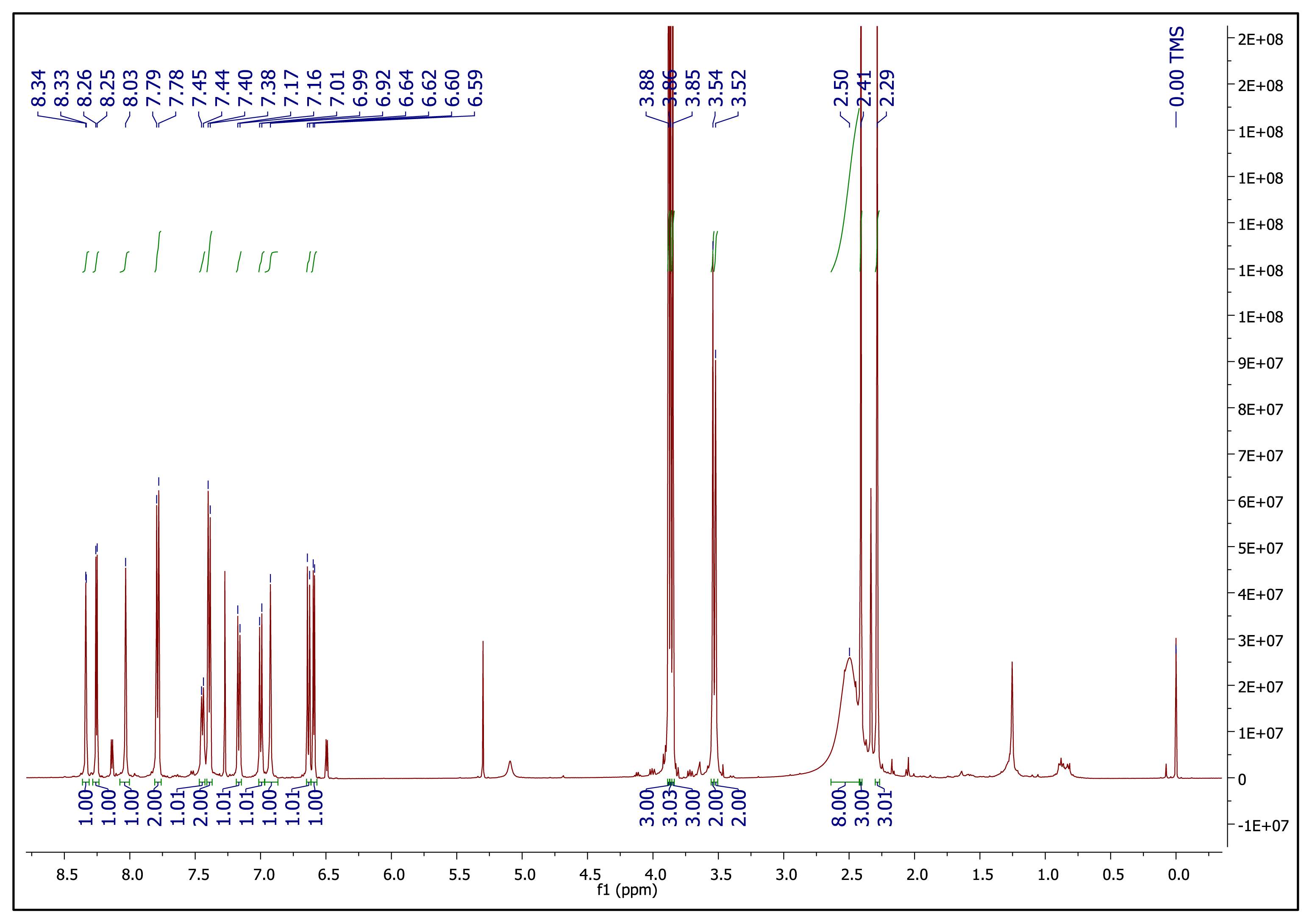

Supplement: Figure S23–S24–S25 — 1H-NMR spectrum of Compound 6. [file turkjchem-46-1-86s22a.tif]

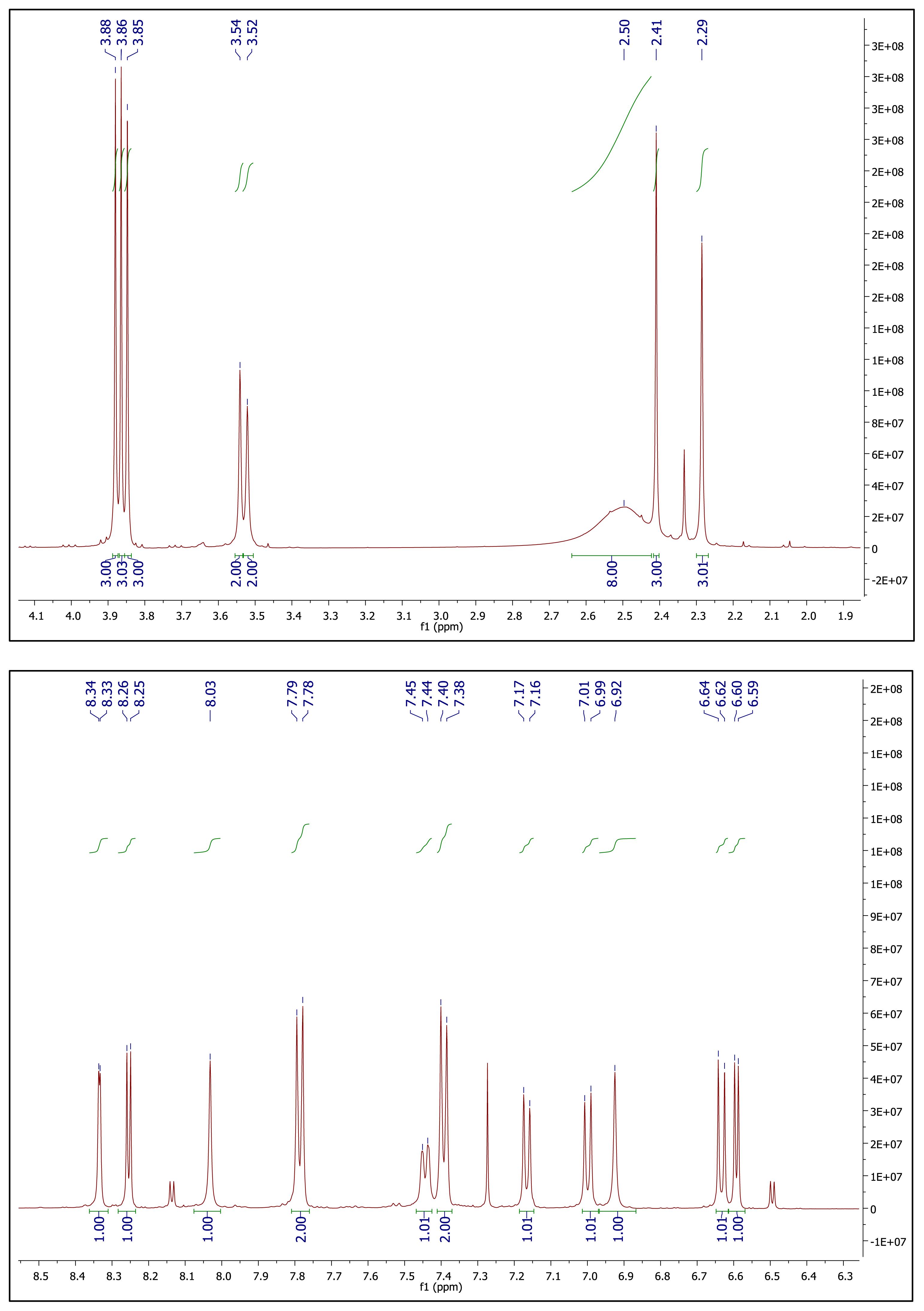

Supplement: Figure S23–S24–S25 — 1H-NMR spectrum of Compound 6. [file turkjchem-46-1-86s22b.tif]

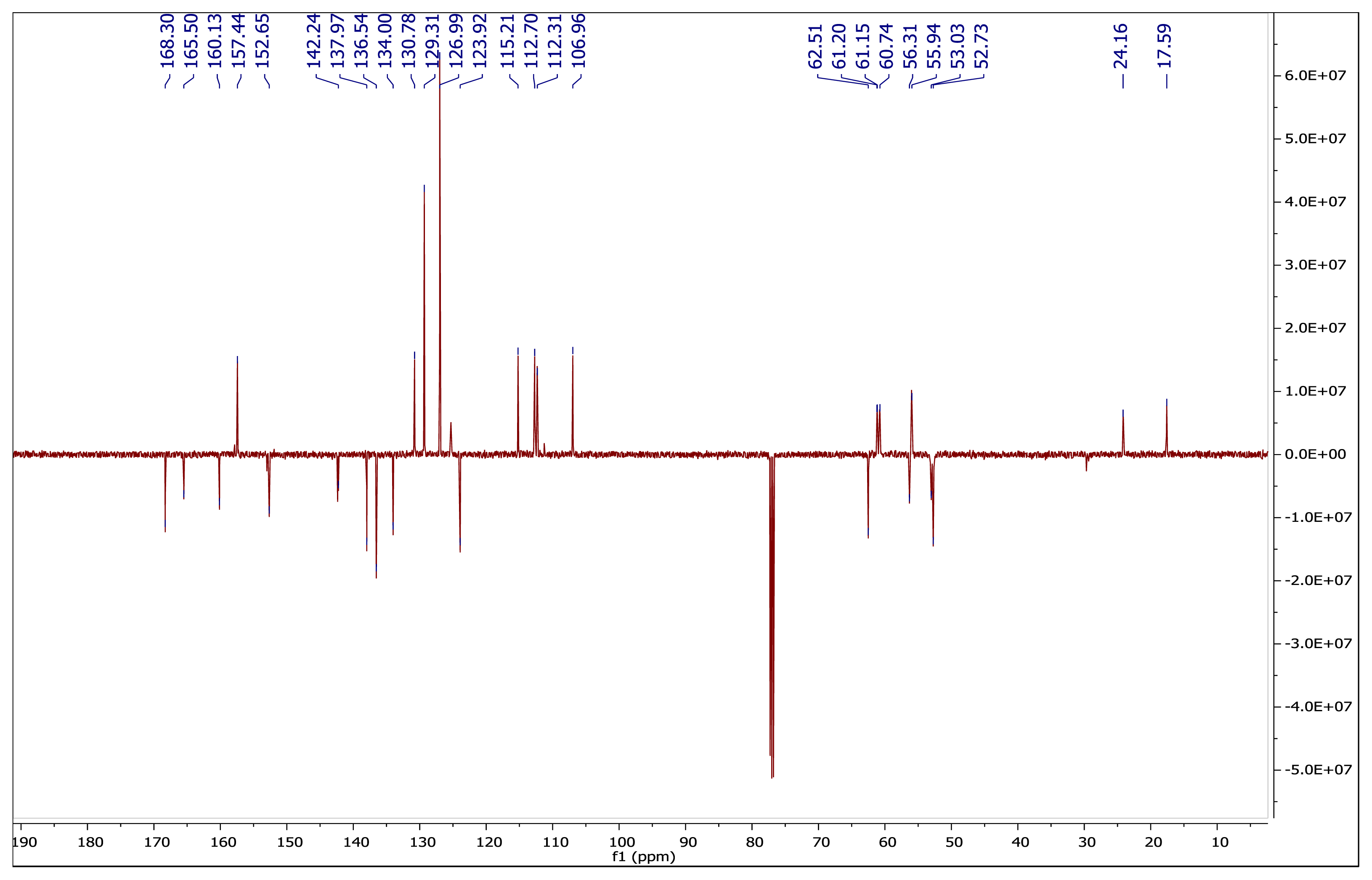

Supplement: Figure S26 — APT spectrum of Compound 6. [file turkjchem-46-1-86s23.tif]

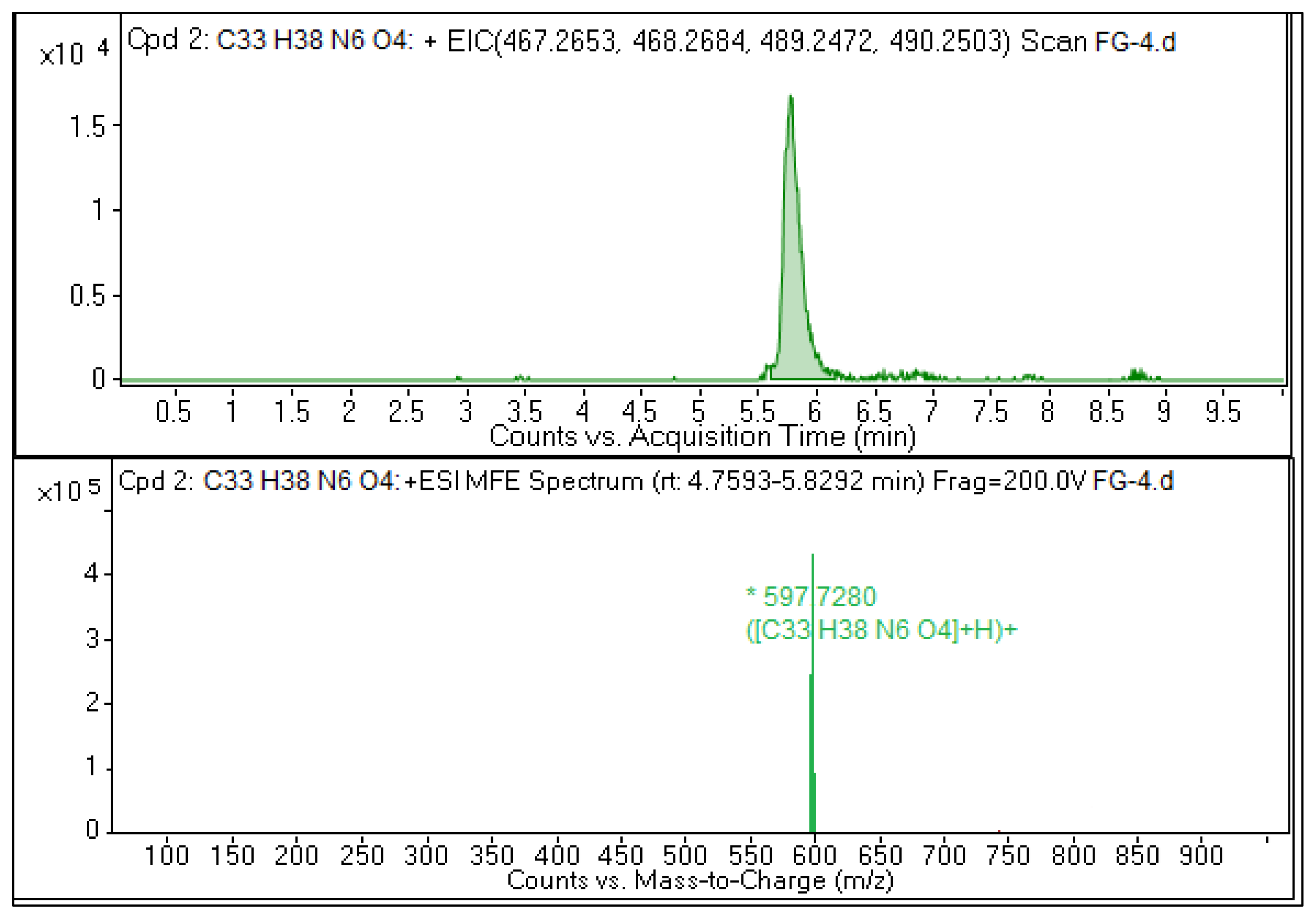

Supplement: Figure S27 — HRMS Spectrum of Compound 6. [file turkjchem-46-1-86s24.tif]

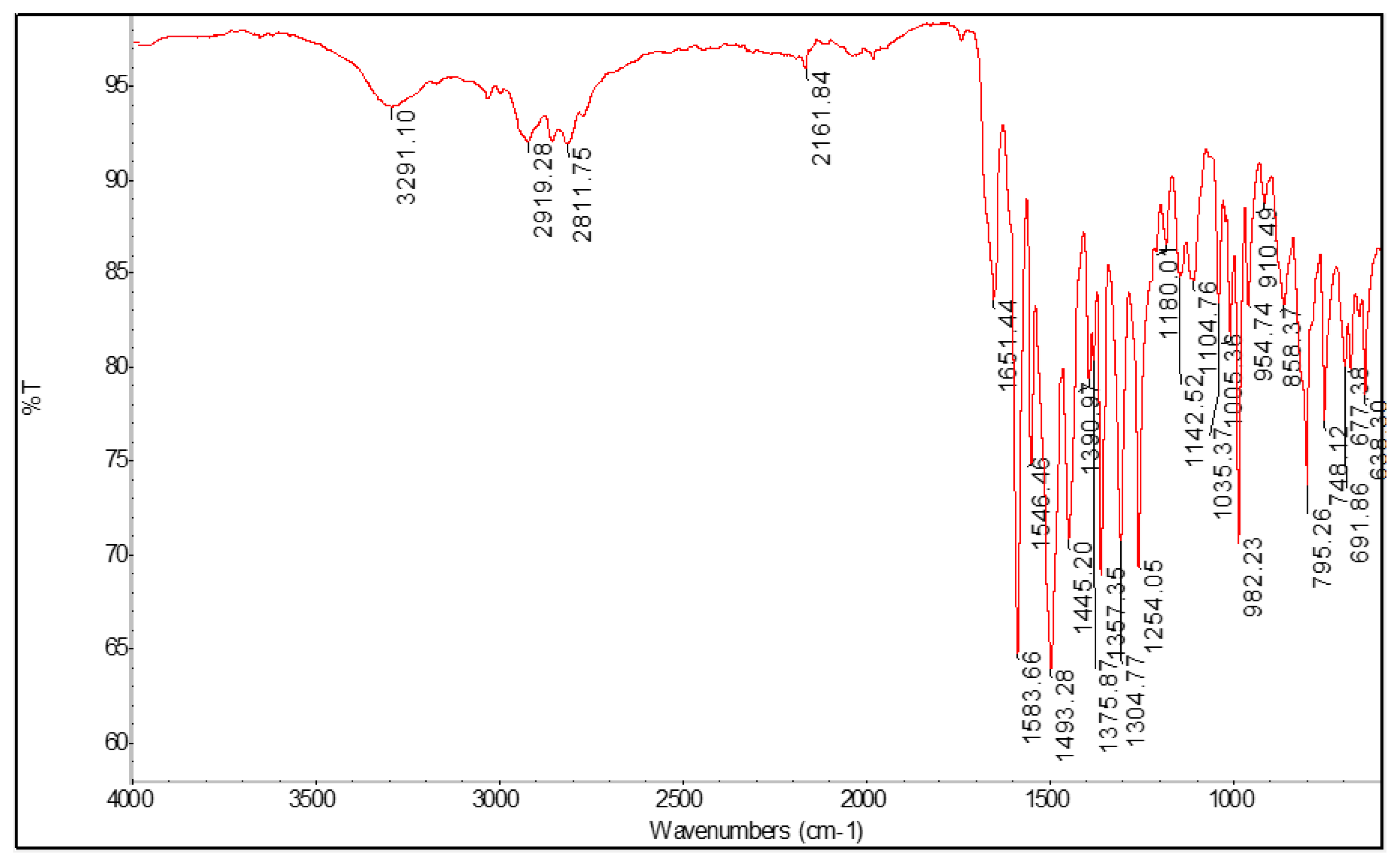

Supplement: Figure S28 — IR spectrum of Compound 7. [file turkjchem-46-1-86s25.tif]

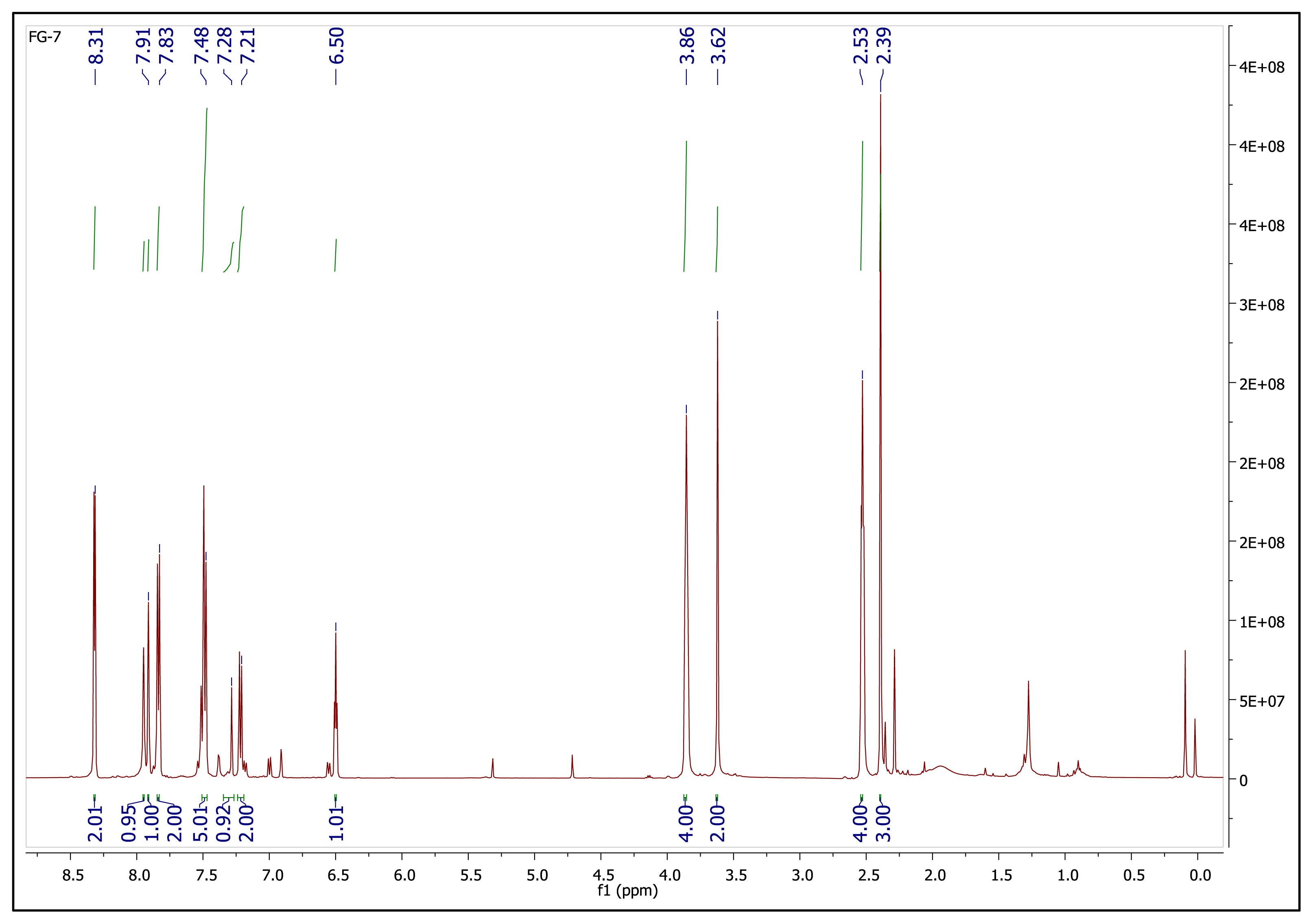

Supplement: Figure S29 — 1H-NMR spectrum of Compound 7. [file turkjchem-46-1-86s26.tif]

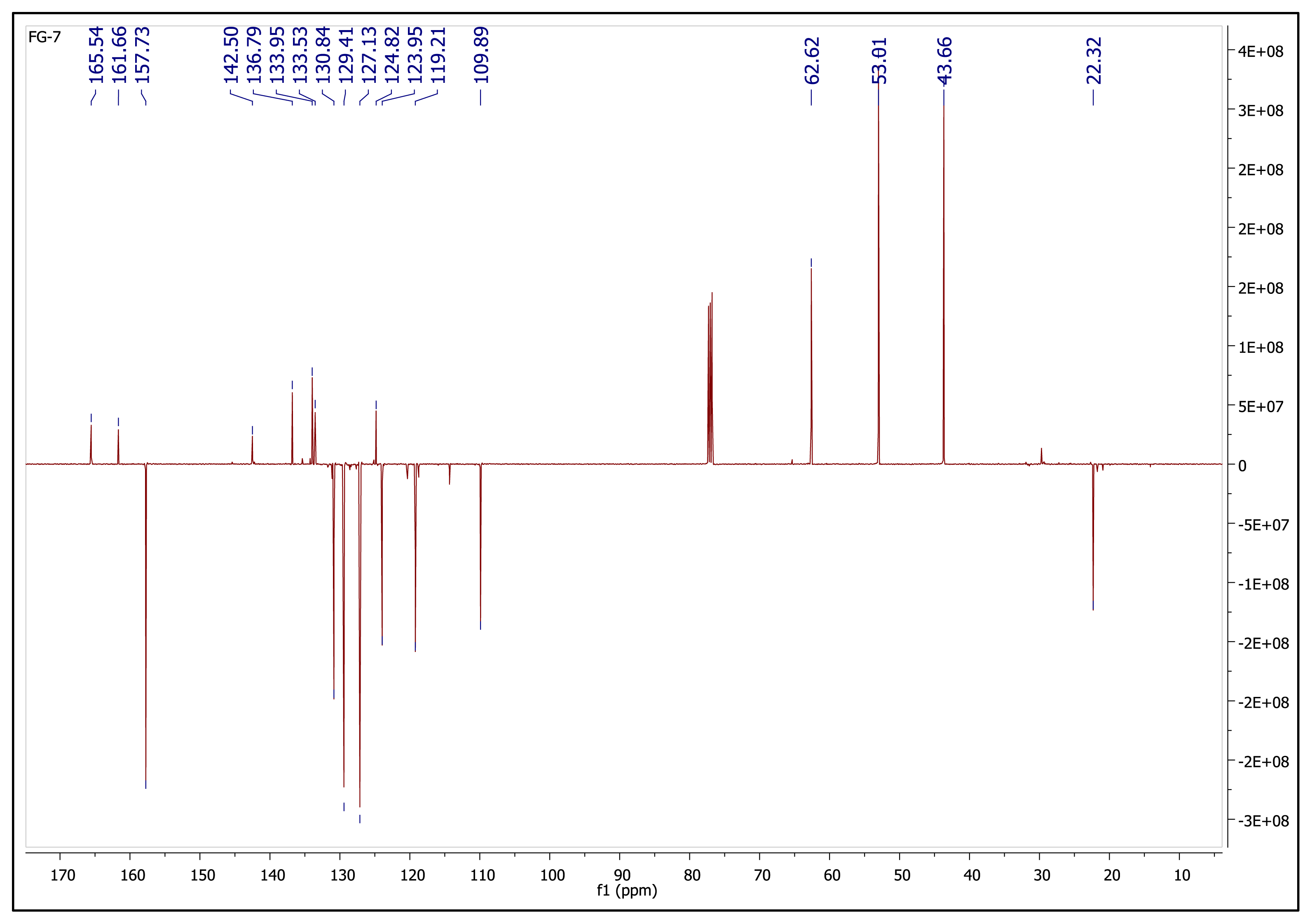

Supplement: Figure S30 — APT spectrum of Compound 7. [file turkjchem-46-1-86s27.tif]

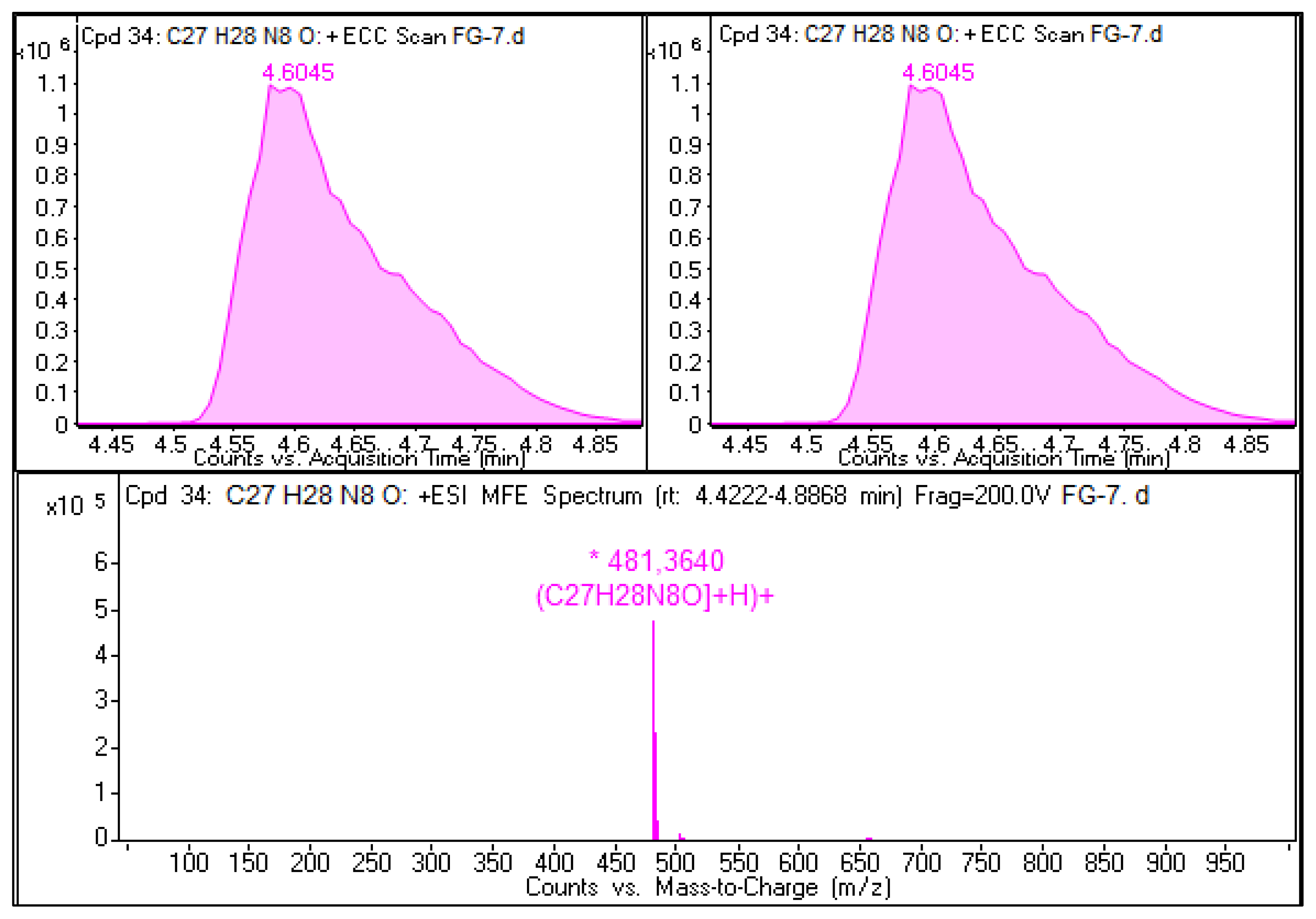

Supplement: Figure S31 — HRMS Spectrum of Compound 7. [file turkjchem-46-1-86s28.tif]

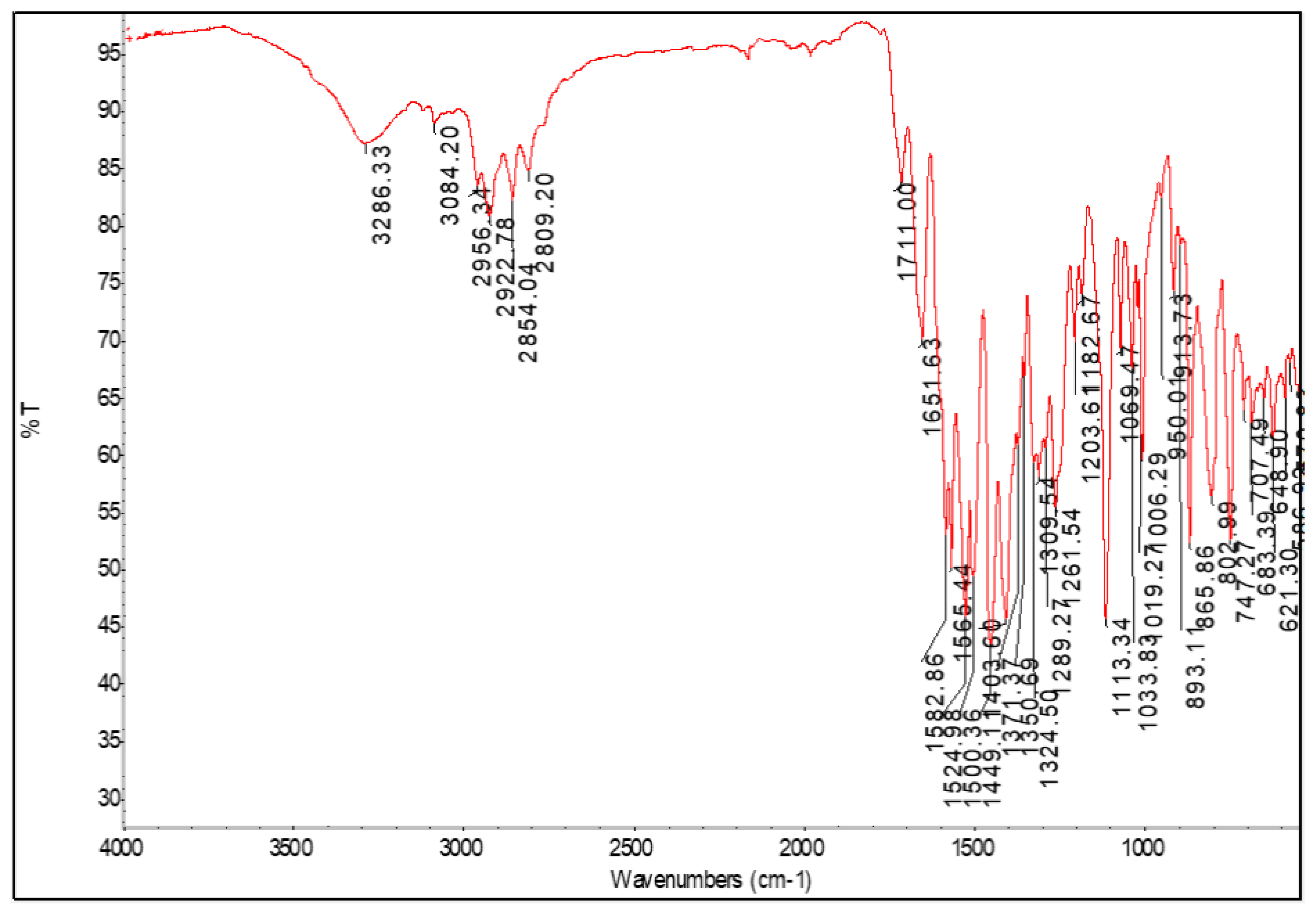

Supplement: Figure S32 — IR spectrum of Compound 8. [file turkjchem-46-1-86s29.tif]

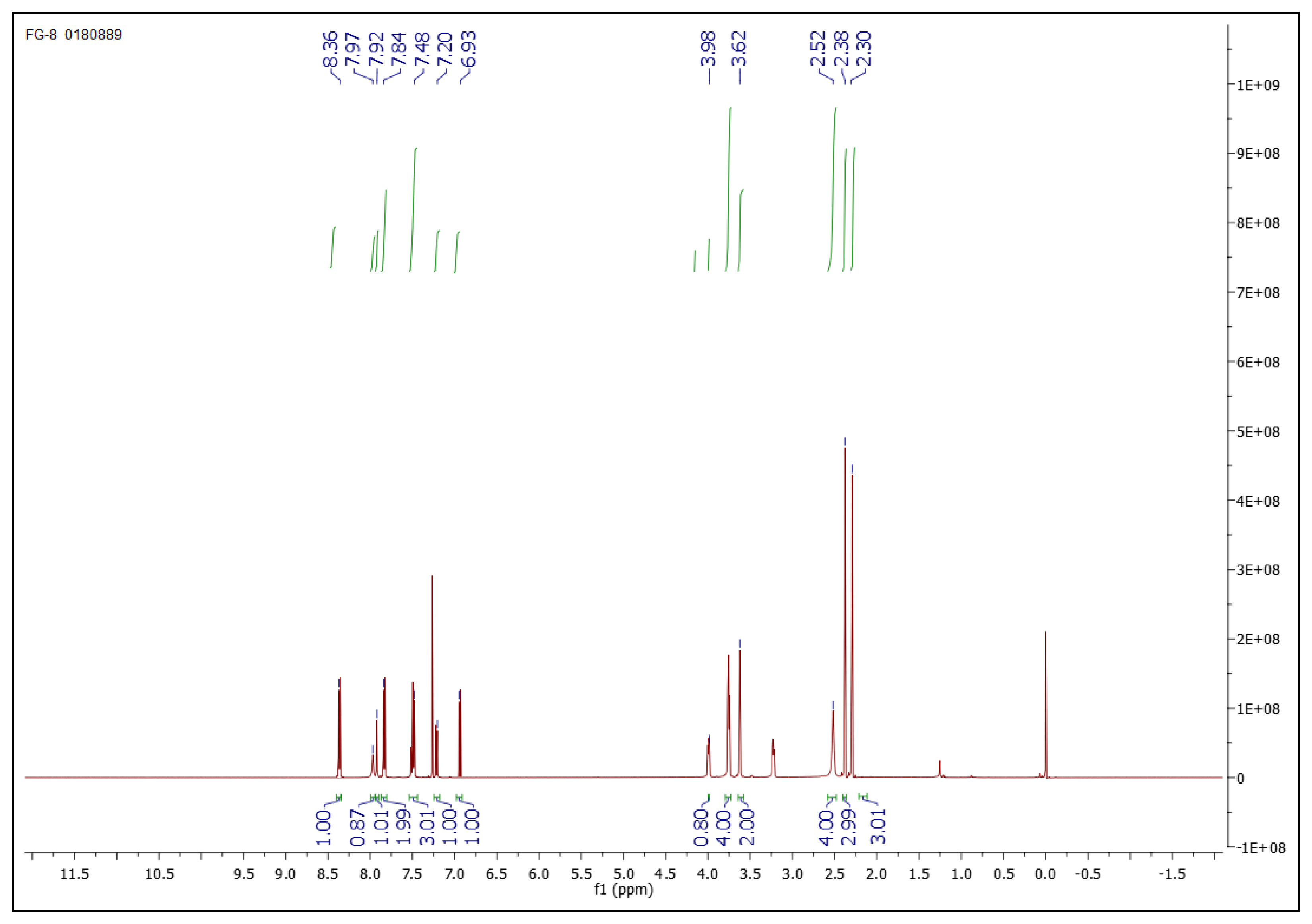

Supplement: Figure S33 — 1H-NMR spectrum of Compound 8. [file turkjchem-46-1-86s30.tif]

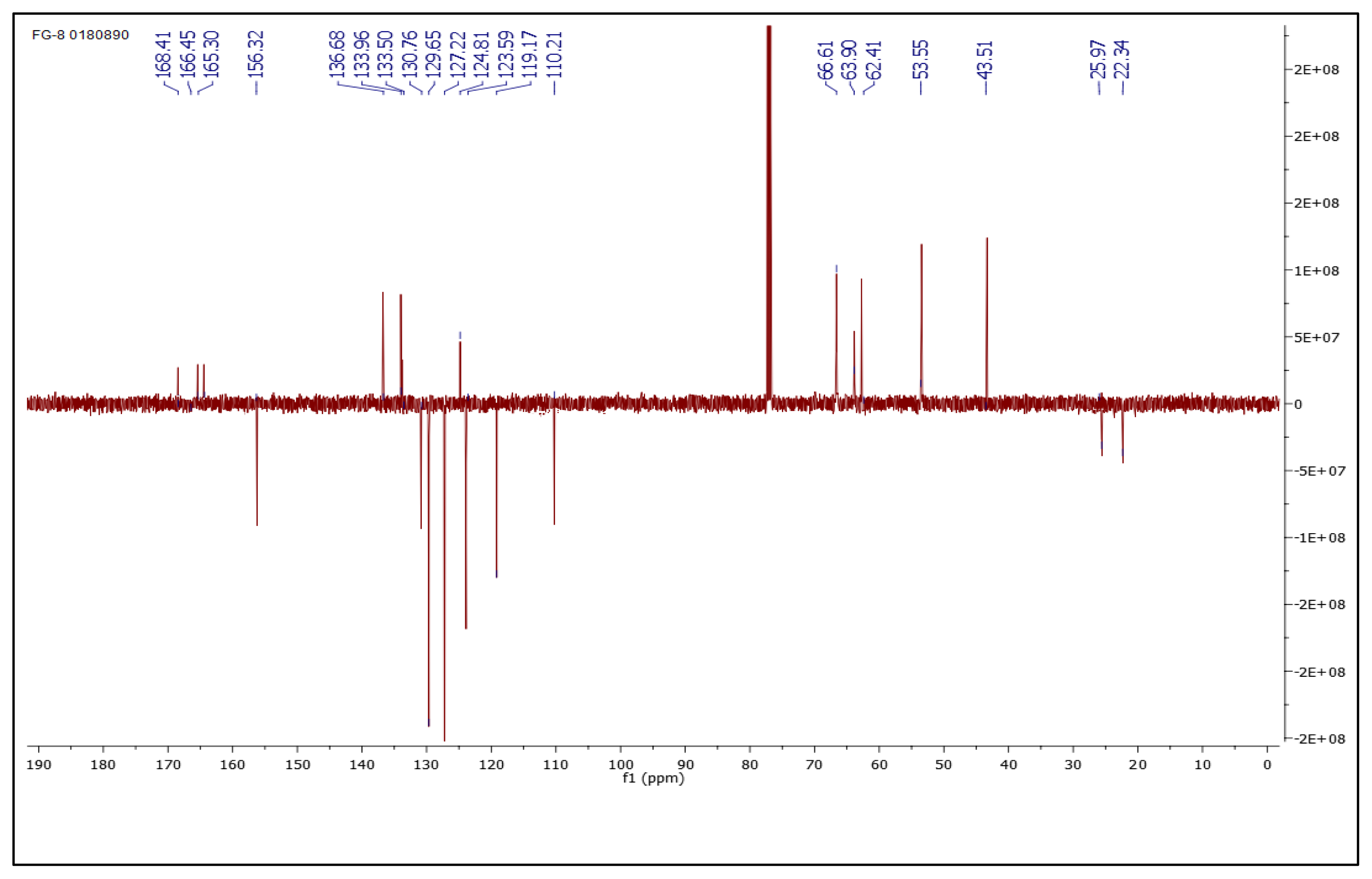

Supplement: Figure S34 — APT spectrum of Compound 8. [file turkjchem-46-1-86s31.tif]

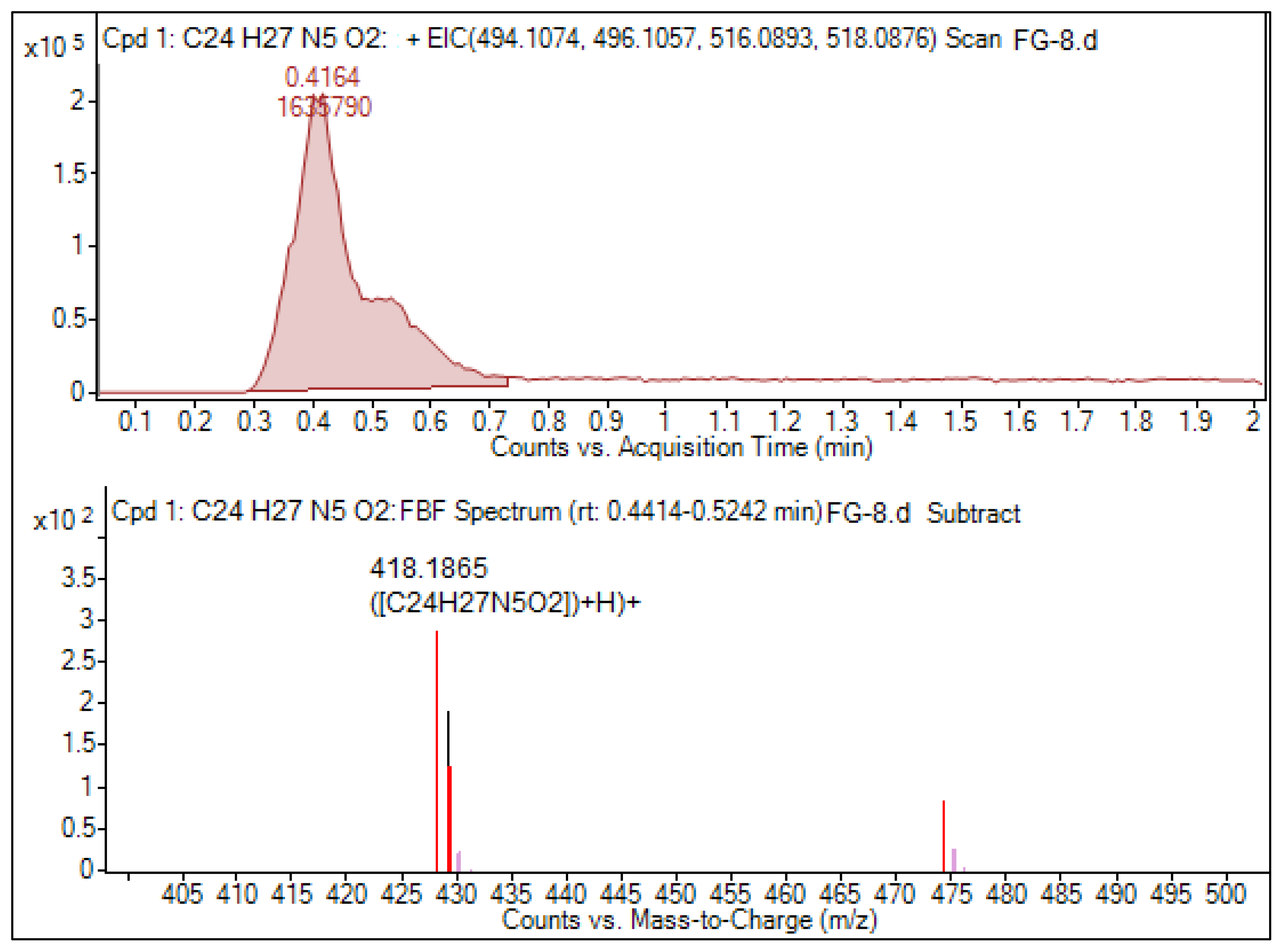

Supplement: Figure S35 — HRMS Spectrum of Compound 8. [file turkjchem-46-1-86s32.tif]

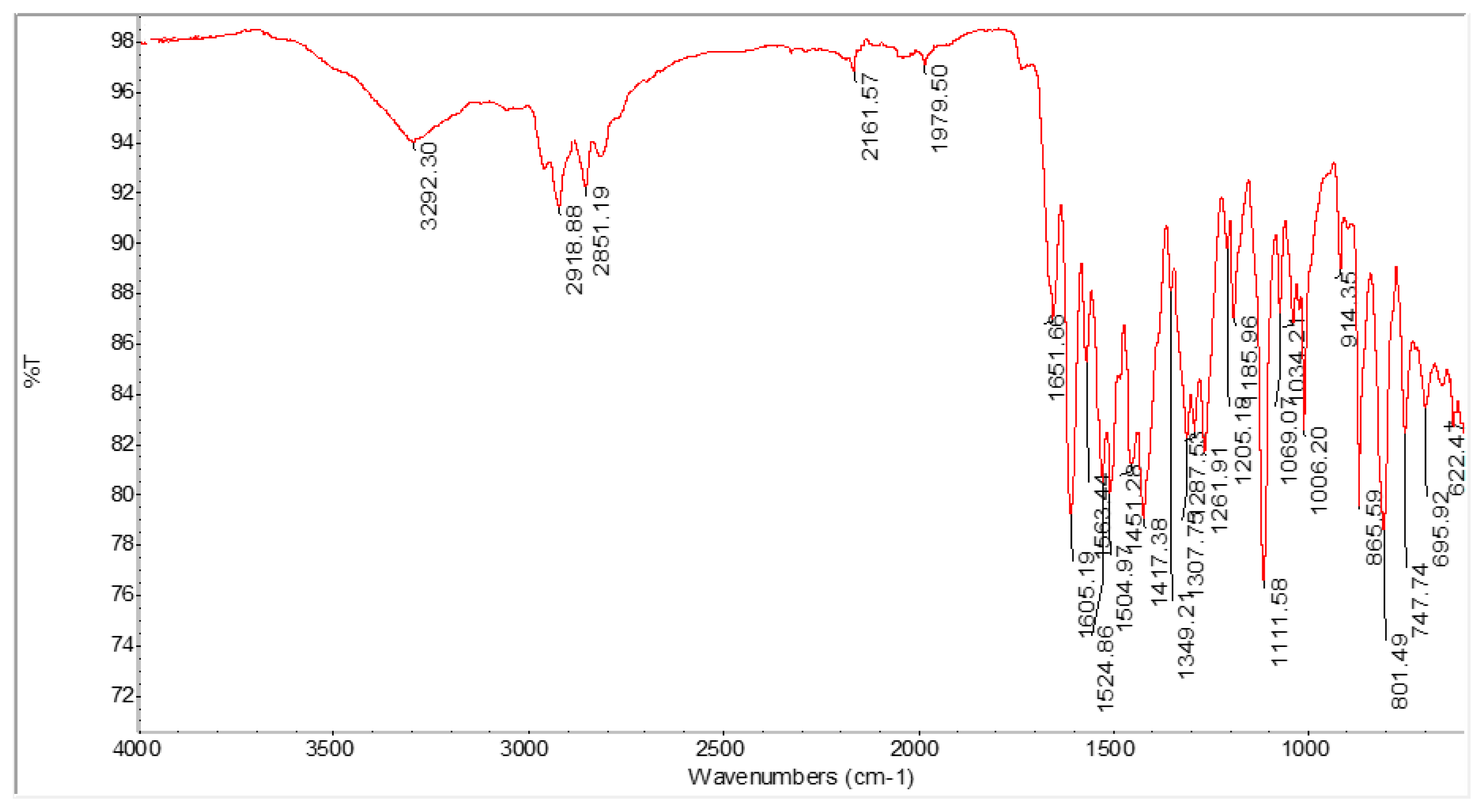

Supplement: Figure S36 — IR spectrum of Compound 9. [file turkjchem-46-1-86s33.tif]

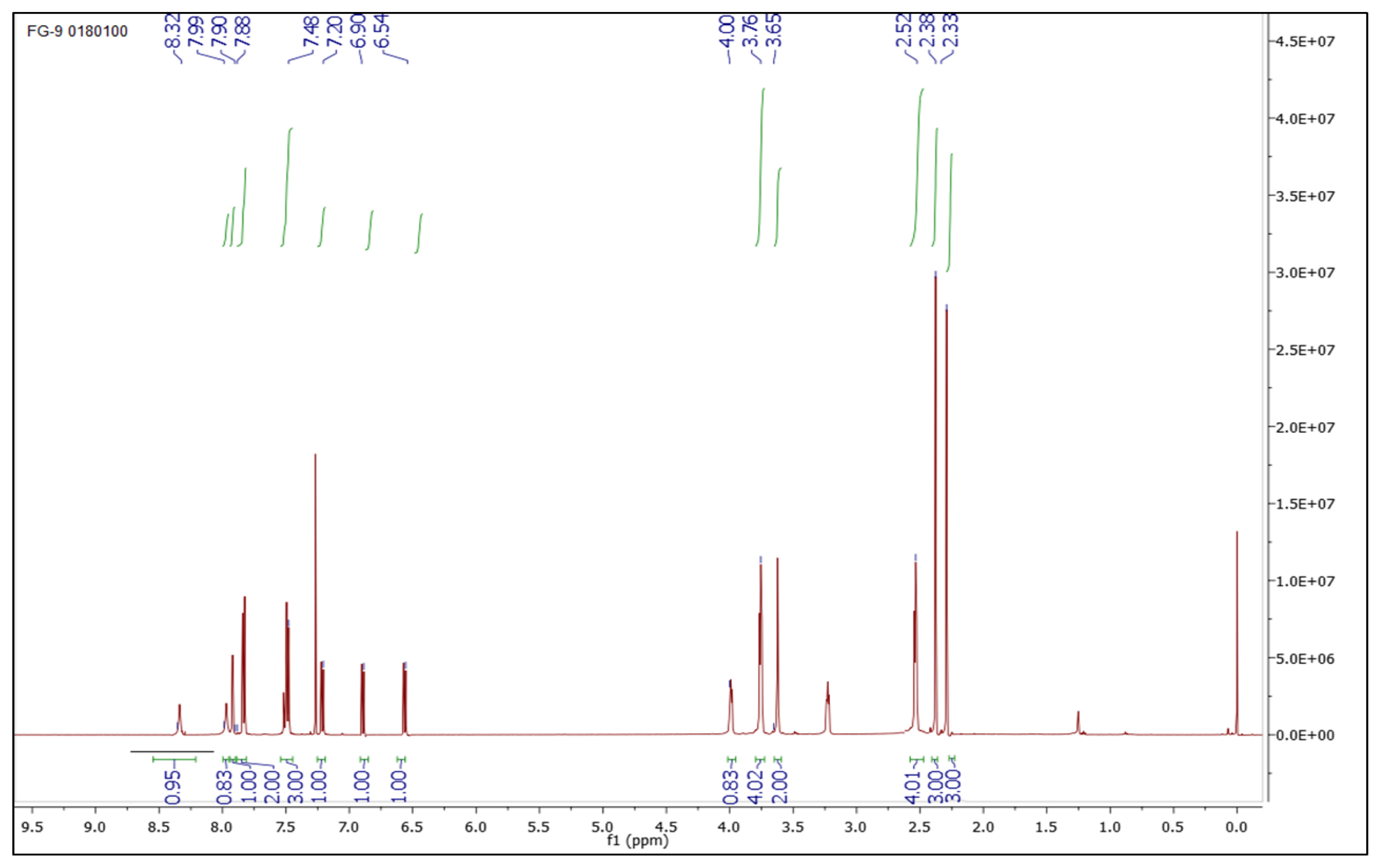

Supplement: Figure S37 — 1H-NMR spectrum of Compound 9. [file turkjchem-46-1-86s34.tif]

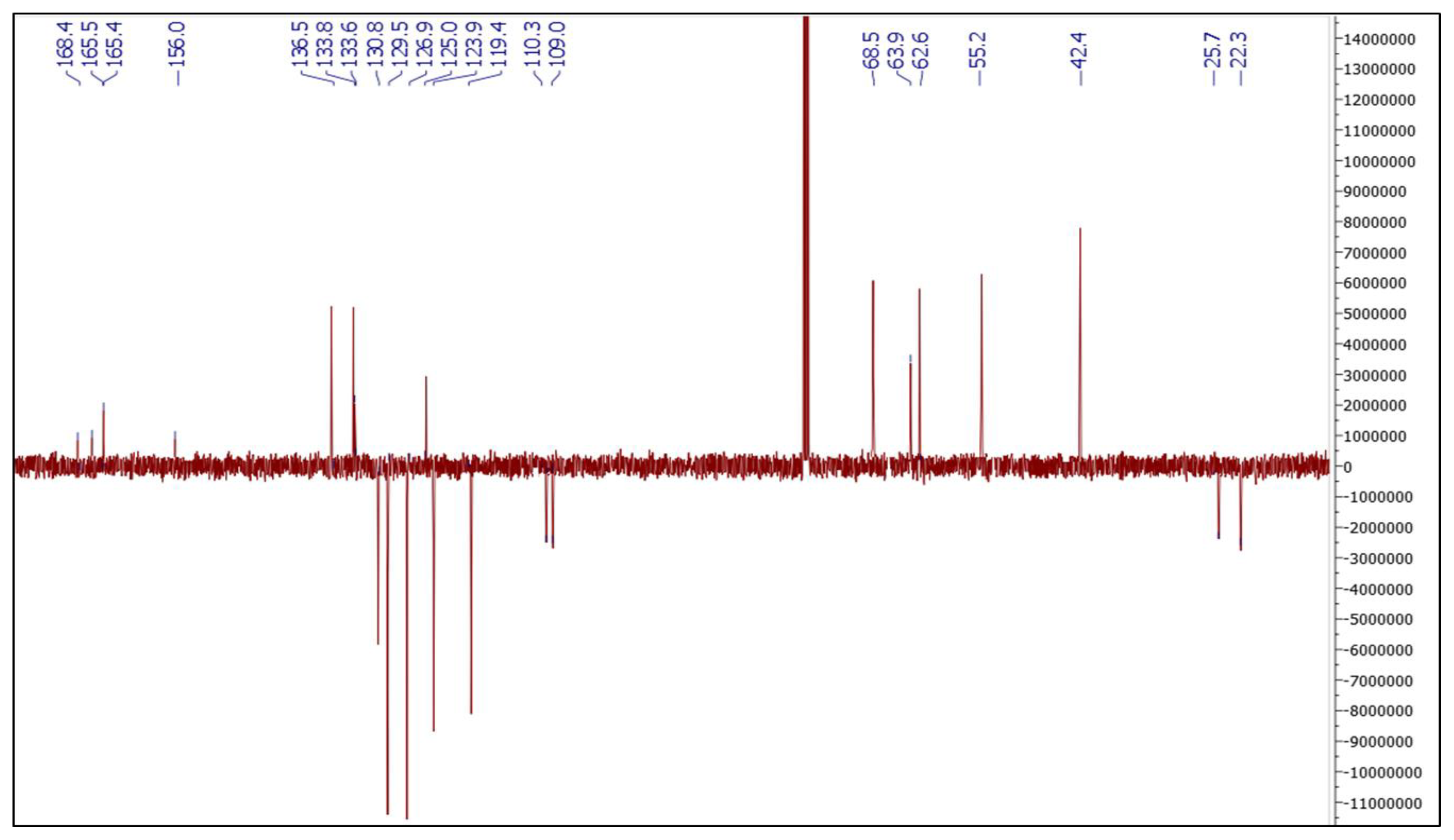

Supplement: Figure S38 — APT spectrum of Compound 9. [file turkjchem-46-1-86s35.tif]

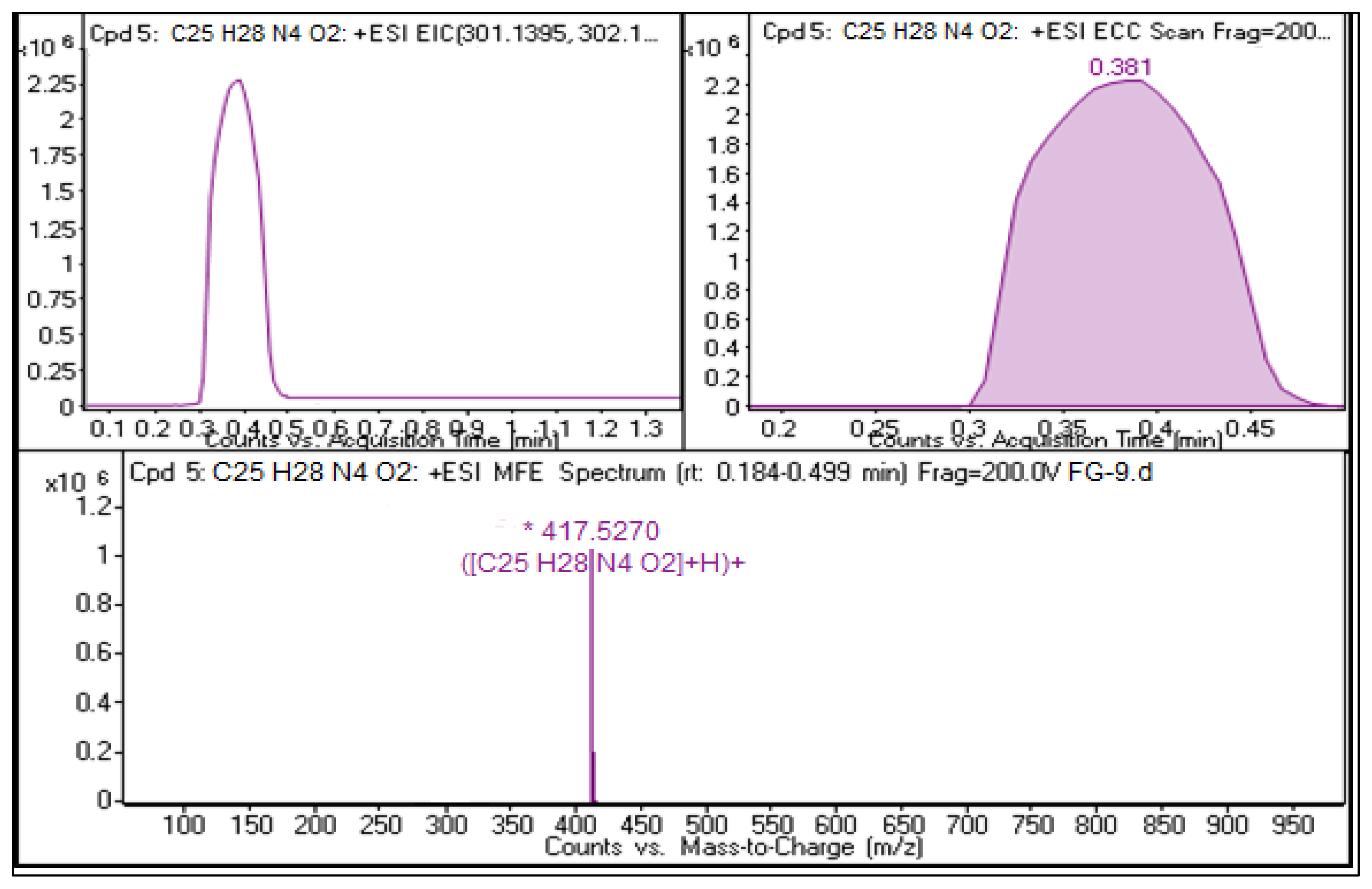

Supplement: Figure S39 — HRMS Spectrum of Compound 9. [file turkjchem-46-1-86s36.tif]

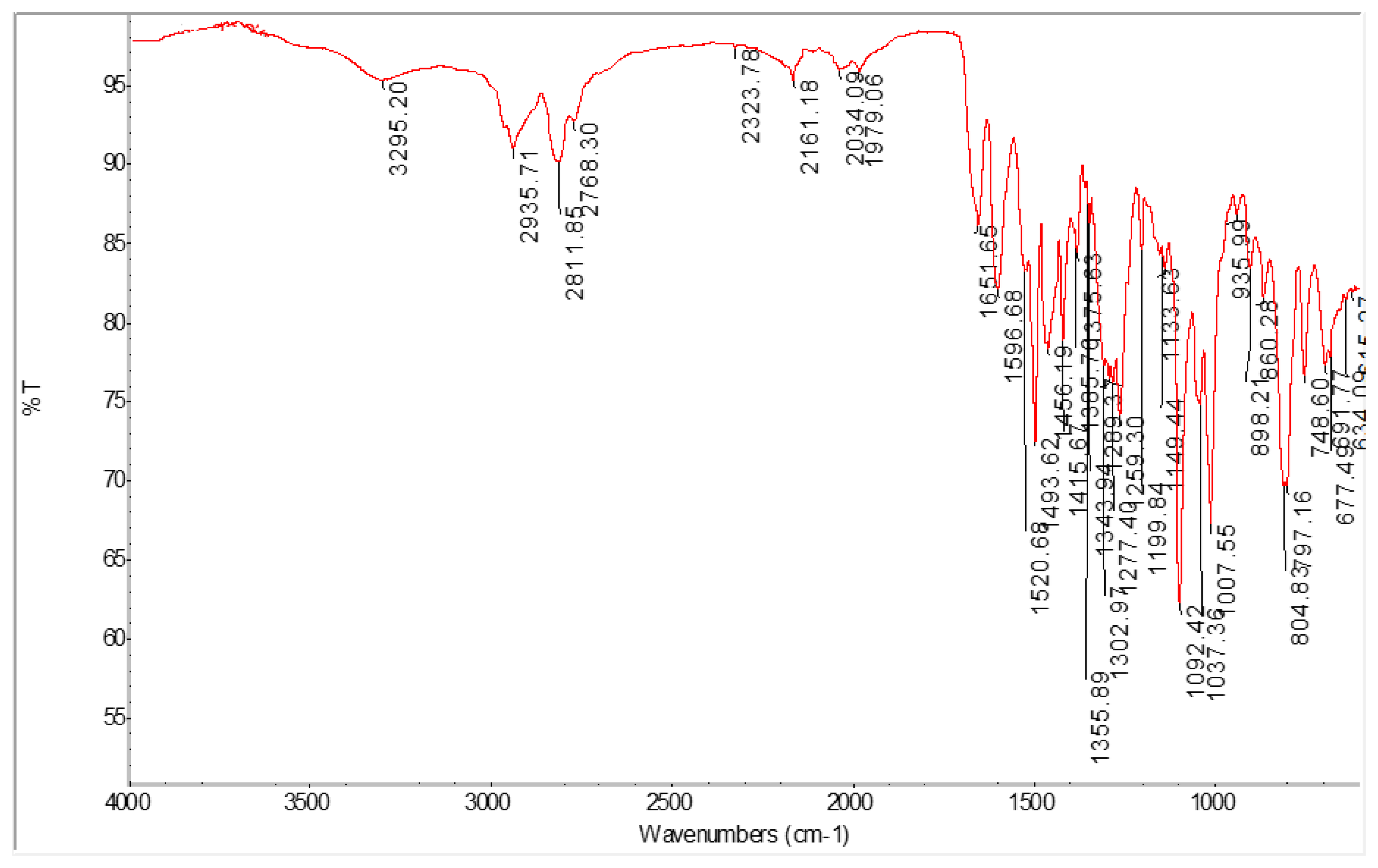

Supplement: Figure S40 — IR spectrum of Compound 10. [file turkjchem-46-1-86s37.tif]

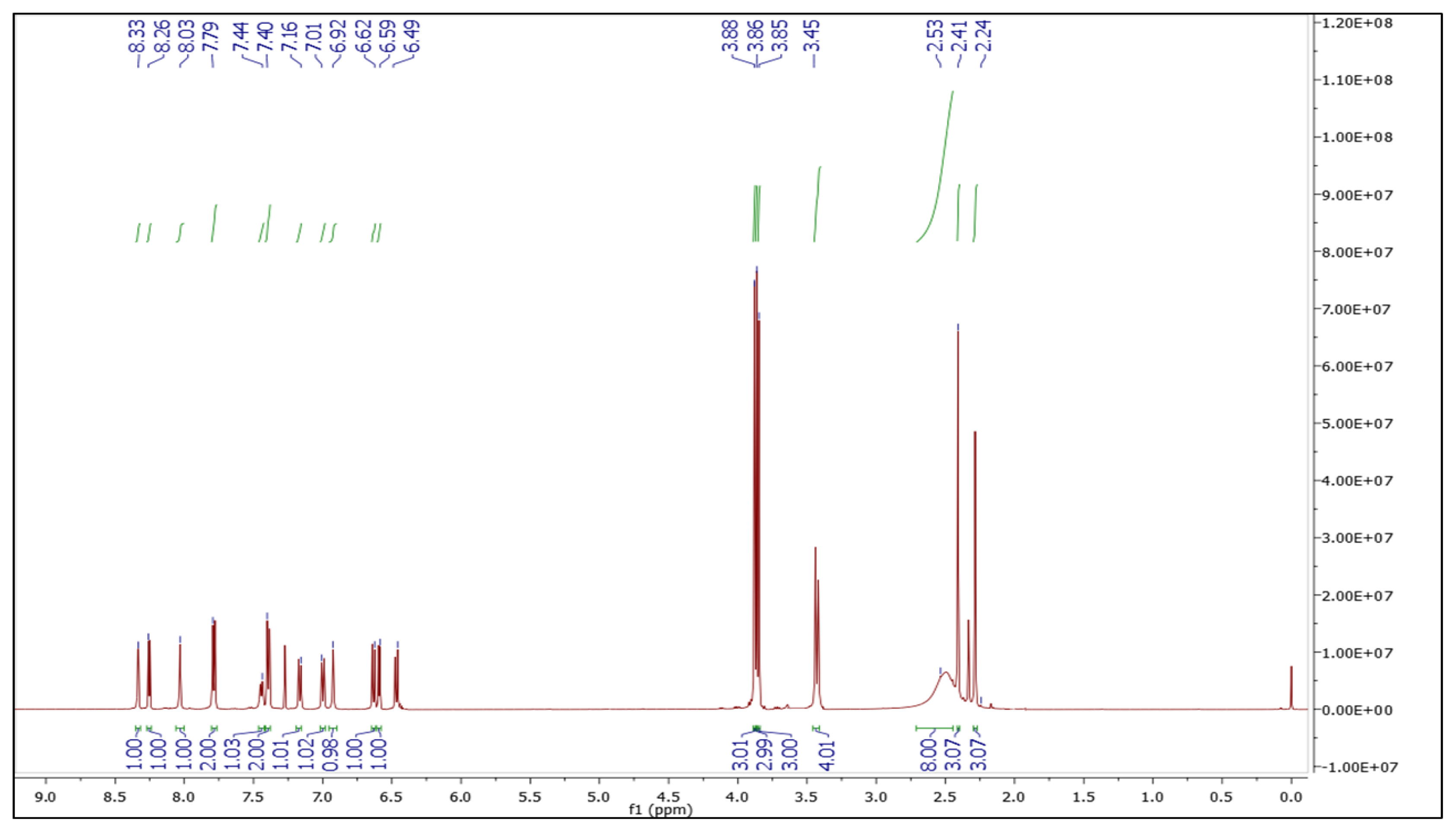

Supplement: Figure S41 — 1H-NMR spectrum of Compound 10. [file turkjchem-46-1-86s38.tif]

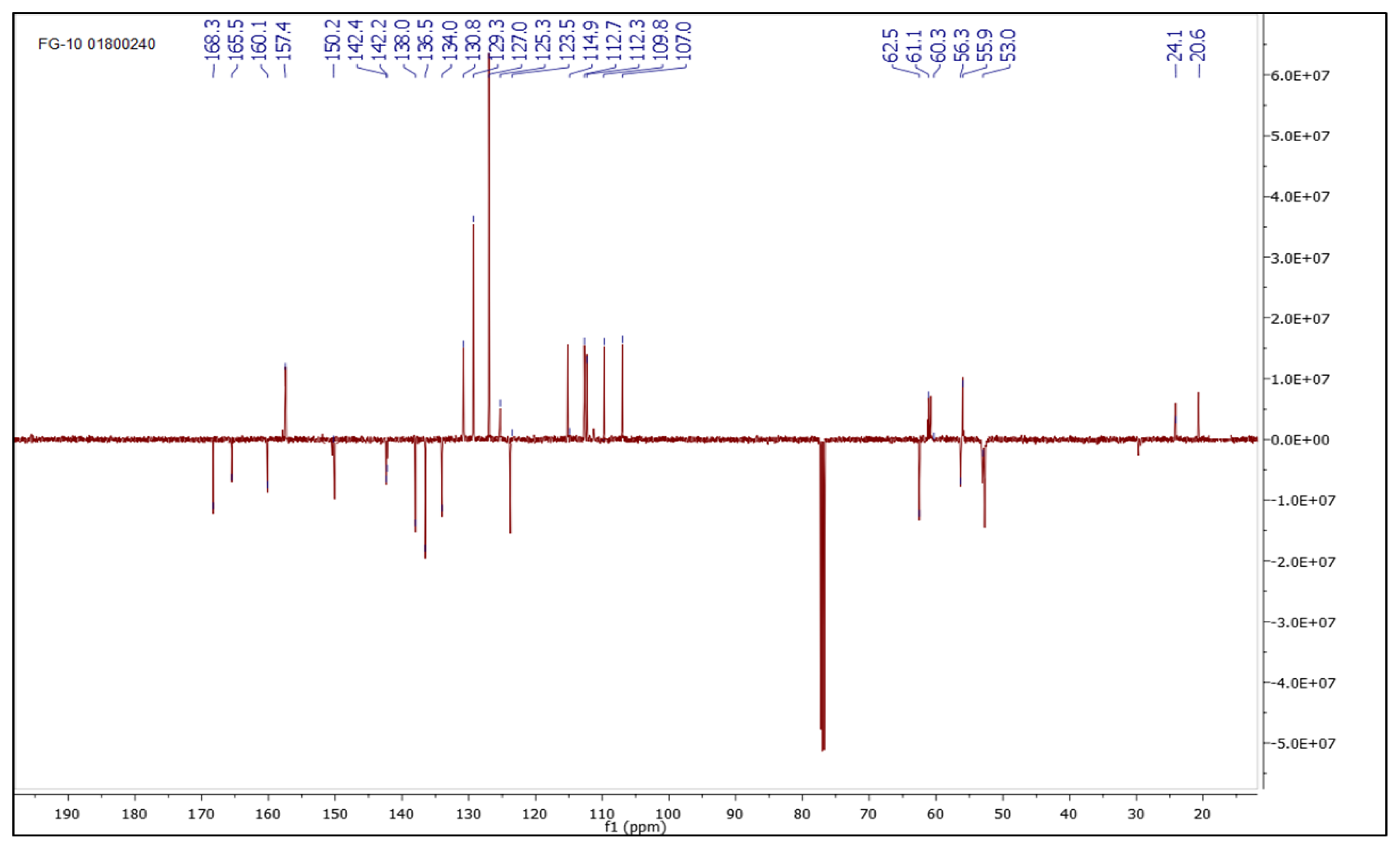

Supplement: Figure S42 — APT spectrum of Compound 10. [file turkjchem-46-1-86s39.tif]

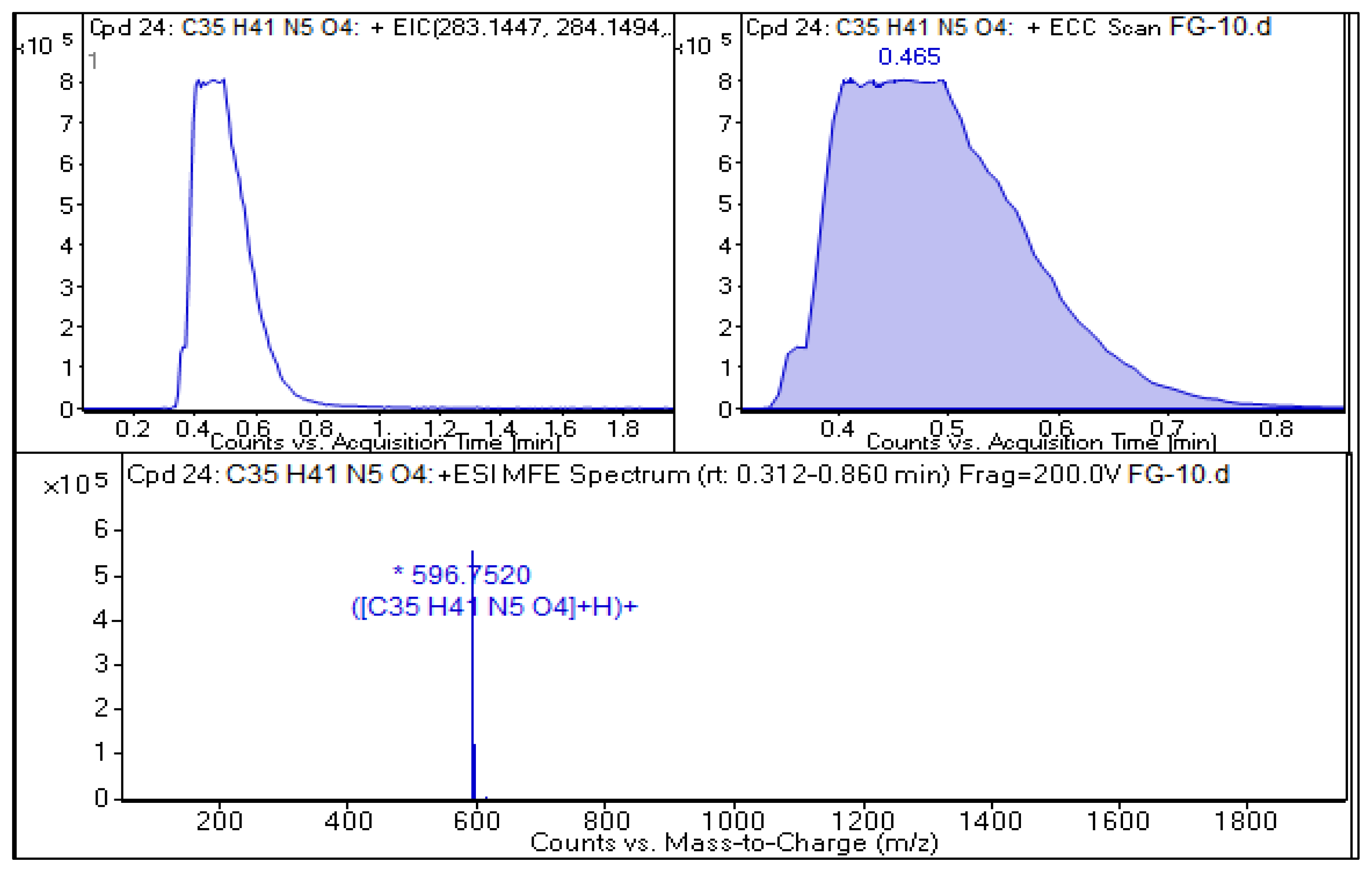

Supplement: Figure S43 — HRMS Spectrum of Compound 10. [file turkjchem-46-1-86s40.tif]

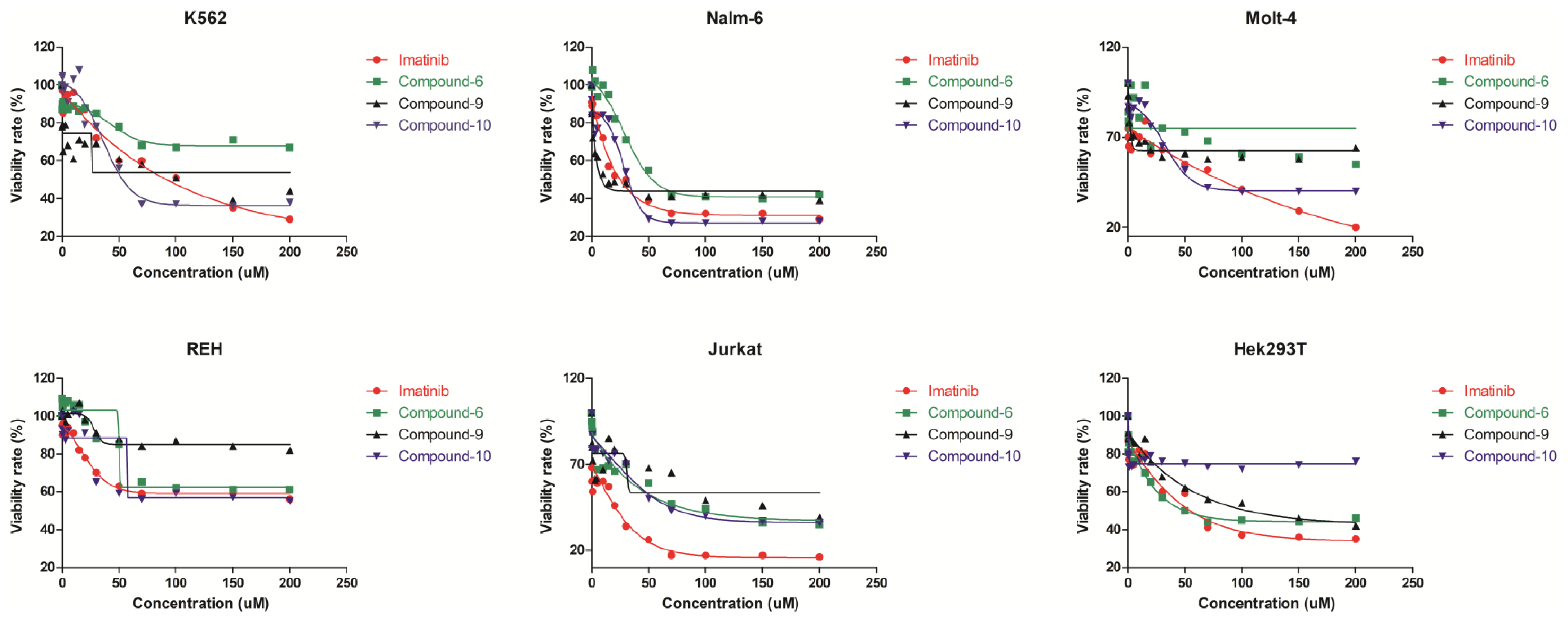

Supplement: Figure S44 — Comparision between imatinib and compound-6–9–10 in all cell-lines. [file turkjchem-46-1-86s41.tif]

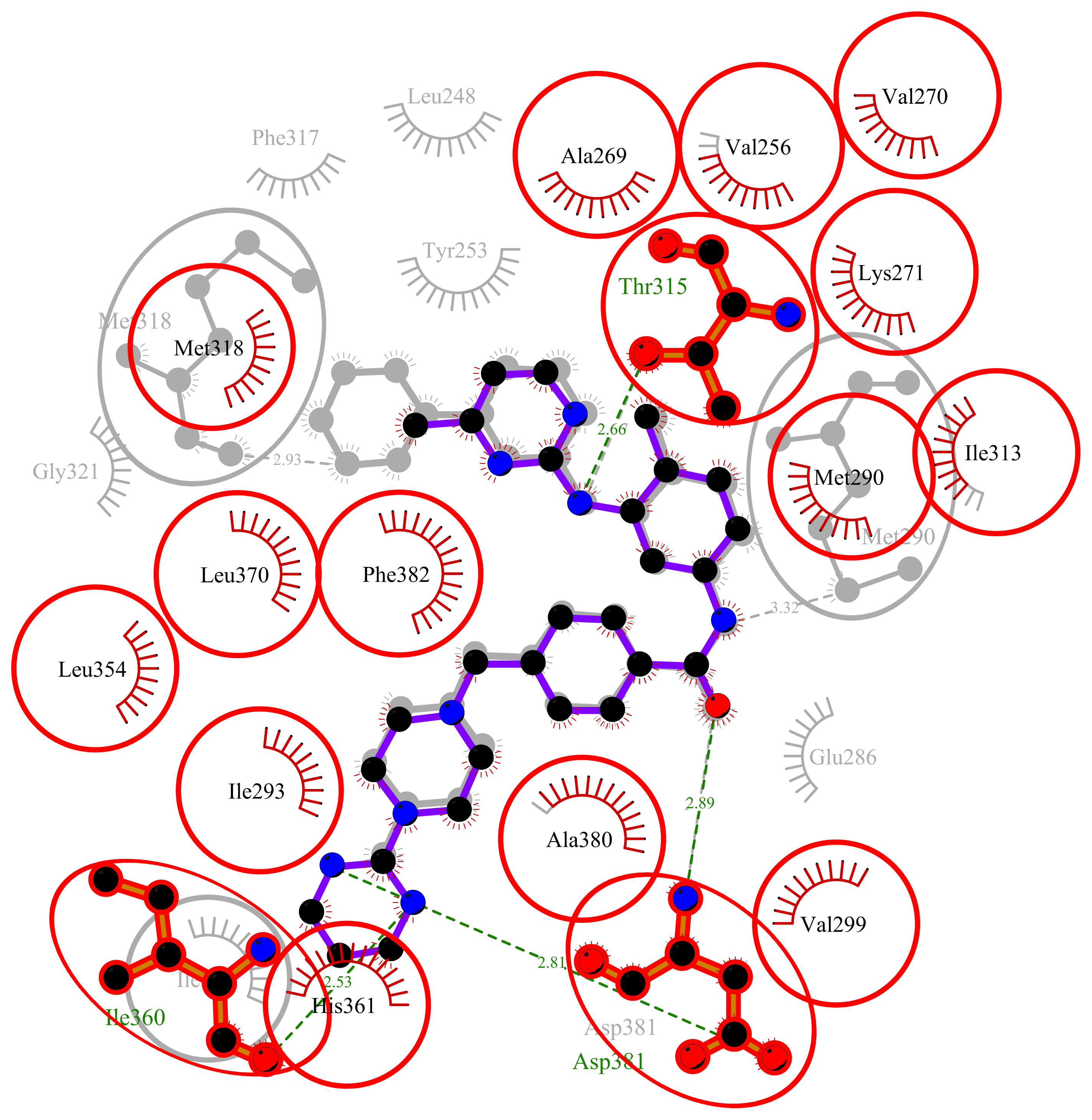

Supplement: Figure S45 — Comparison of ABL-imatinib (background) and ABL-Compound 5 (foreground) interactions. Imatinib and its contact residues are depicted in gray, while the common contact residues are marked with red circles. [file turkjchem-46-1-86s42.tif]

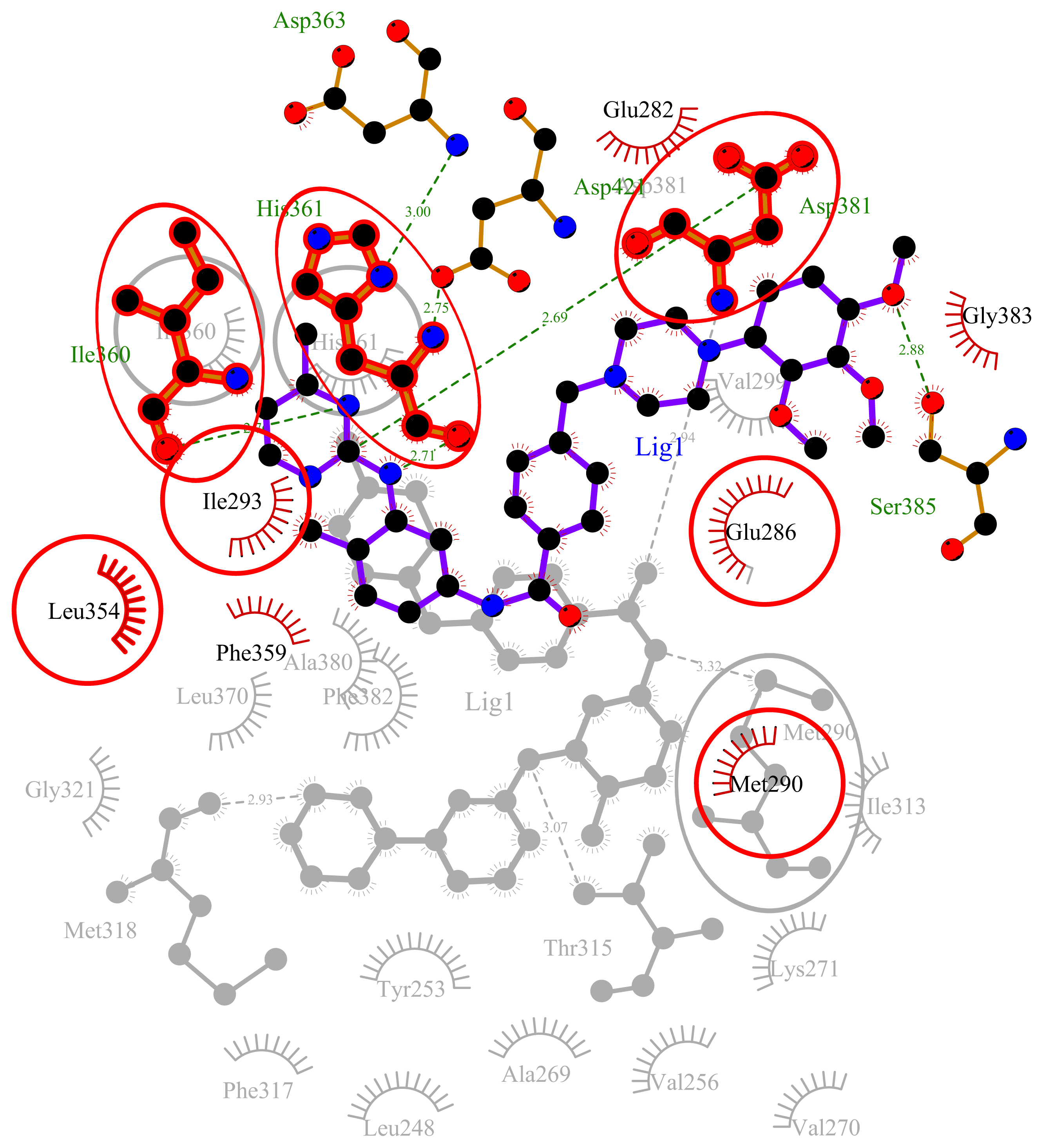

Supplement: Figure S46 — Comparison of ABL-imatinib (background) and ABL-Compound 6 (foreground) interactions. Imatinib and its contact residues are depicted in gray, while the common contact residues are marked with red circles. [file turkjchem-46-1-86s43.tif]

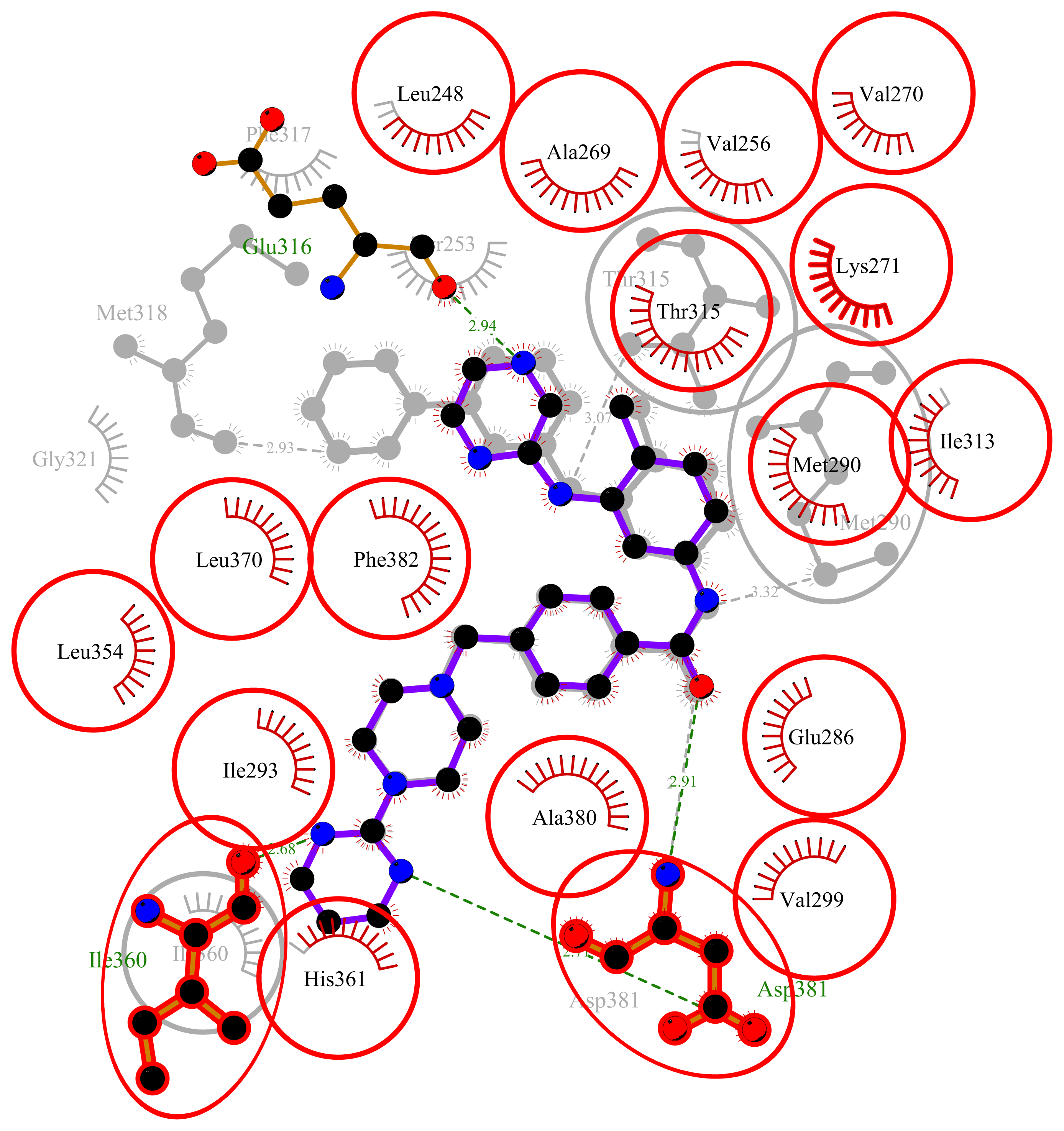

Supplement: Figure S47 — Comparison of ABL-imatinib (background) and ABL-Compound 7 (foreground) interactions. Imatinib and its contact residues are depicted in gray, while the common contact residues are marked with red circles. [file turkjchem-46-1-86s44.tif]

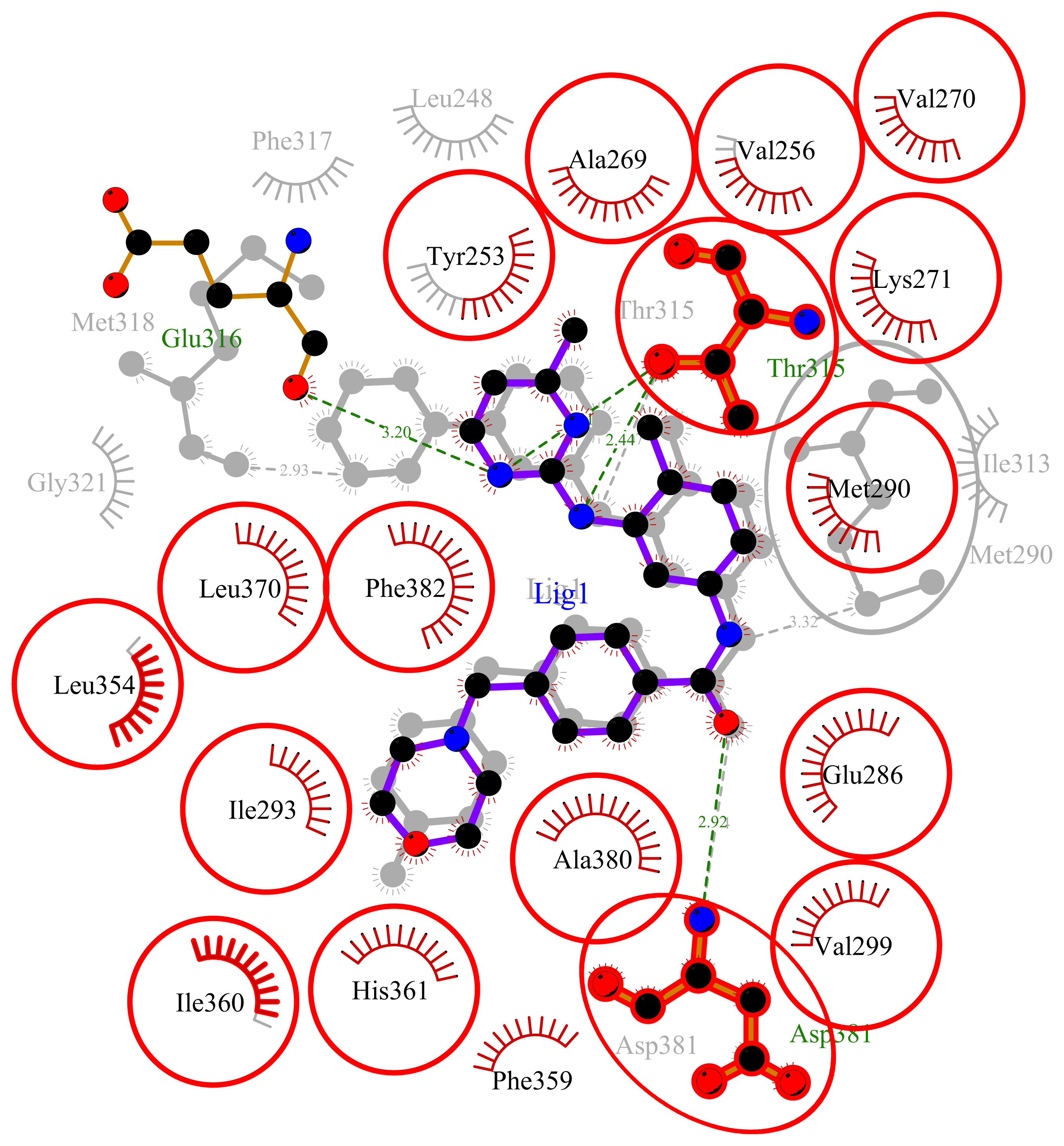

Supplement: Figure S48 — Comparison of ABL-imatinib (background) and ABL-Compound 8 (foreground) interactions. Imatinib and its contact residues are depicted in gray, while the common contact residues are marked with red circles. [file turkjchem-46-1-86s45.tif]

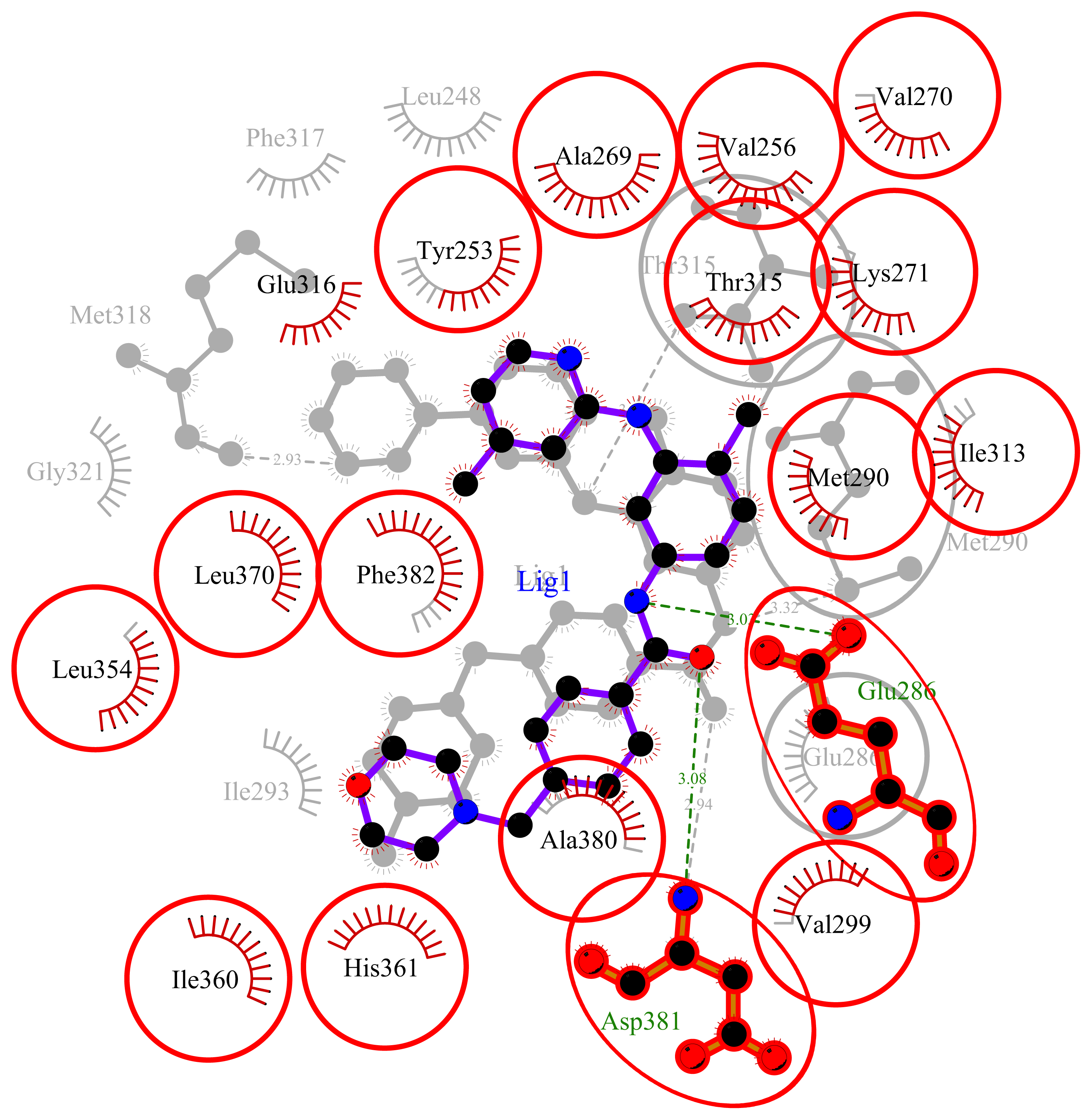

Supplement: Figure S49 — Comparison of ABL-imatinib (background) and ABL-Compound 9 (foreground) interactions. Imatinib and its contact residues are depicted in gray, while the common contact residues are marked with red circles. [file turkjchem-46-1-86s46.tif]
